# Supplementary material for: Indirect effects of the early phase of the COVID-19 pandemic on the coverage of essential maternal and newborn health services in a rural subdistrict in Bangladesh: results from a cross-sectional household survey
Source: BMJ Open. 2022 Feb 3;12(2):e056951. doi: 10.1136/bmjopen-2021-056951 (PMC8814430; doi:10.1136/bmjopen-2021-056951)

Supplementary Table 1: Summary of indicator selected for each target population with required sample size

Target population	Indicator	Baseline (pre-pandemic period)	Endline (Pandemic period)	Unadjusted sample size	Adjustedd Sample Size
Women who were third trimester pregnant during the COVID-19' pandemic' period and the selected pre-pandemic period in the preceding year	At least one ANC from MTP<br>At least one ANC from facility	60%	30%	113	126
Women who had a history of birth during the COVID-19' pandemic' period and the selected pre-pandemic period in the preceding year	Birth attended by an MTP<br>Birth in a health facility<br>At least one PNC from an MTP within 48 hours of birth<br>Newborns received essential newborn care (ENC)	50%	33%	160	178

Supplementary Table 2: Definition of selected indicator

Selected indicators	Definitions
Indicator 1	Proportion of women who were third trimester pregnant during the COVID-19' pandemic' period and the pre-pandemic period in the preceding year receiving at least one ANC from a medically trained provider (MTP)
Indicator 2	Proportion of women who were third trimester pregnant during the COVID-19' pandemic' period and the pre-pandemic period in the preceding year receiving at least one ANC from facility
Indicator 3	Proportion of women giving birth during COVID-19' pandemic' period and the corresponding period in the preceding year were attended by an MTP
Indicator 4	Proportion of women giving birth during COVID-19' pandemic' period and the corresponding period in the preceding year at a facility
Indicator 5	Proportion of women giving birth during the COVID-19' pandemic' period and the pre-pandemic period in the preceding year receiving PNC from an MTP within 48 hours of birth
Indicator 6	Proportion of newborns having born during the COVID-19' pandemic' period and the pre-pandemic period in the preceding year received drying
Indicator 7	Proportion of newborns having born during the COVID-19' pandemic' period and the pre-pandemic period in the preceding year had clean cord cutting
Indicator 8	Proportion of newborns having born during the COVID-19' pandemic' period and the pre-pandemic period in the preceding year received 7.1% chlorohexidine
Indicator 9	Proportion of newborns having born during the COVID-19' pandemic' period and the pre-pandemic period in the preceding year had delayed bathing
Indicator 10	Proportion of newborns having born during the COVID-19' pandemic' period and the pre-pandemic period in the preceding year had early initiation of breastfeeding

Supplementary Table 3: Definition of providers and facilities

Type of providers		Definition
Medically trained provider (MTP)	Doctors (General physicians)	This provider has five years of training on medicine, surgery, gynaecology, and obstetrics with one-year internship in medicine, surgery, gynaecology, and obstetrics wards. This provider is qualified to provide ANC, Basic emergency obstetric services (BEOC), PNC, menstrual regulation (MR), abortion care (AC), family planning services (FPS).
	Nurse	This provider completes 3-4 years of nursing course with 6 months dedicated to obstetrician training. This provider is qualified to provide ANC, BEOC, PNC, MR, AC, FPS.
	Midwife	This provider completes one year of midwifery training with 6 months dedicated to obstetrician training. This provider is qualified to provide ANC, BEOC, PNC, MR, AC, FPS.
	Family welfare visitor (FWV)	This provider completes 18 months of training on maternal and child health, family planning, and contraception. This provider is qualified to provide facility-based ANC, BEOC, PNC, MR, AC, FPS.
	Community Skilled Birth Attendant (CSBA)	This provider completes 6 months of training on BEOC and ENC. This provider is qualified to provide community-based ANC, BEOC, PNC, FPS.
Non-medically trained provider (non-MTP)	Family Welfare Assistant (FWA)	This provider completes 30 days of training on family planning. This provider is qualified to provide community-based FPS.
	Health assistant (HA)	This provider completes 3 months of training on limited preventive and curative care, immunization. This provider is qualified to provide community-based immunization of mother and children.
	Sub Assistant Community Medical Officer (SACMO)	This provider does not have any obstetrician training.
	Community Health Care Provider (CHCP)	This provider does not have any obstetrician training.
	Medical assistants (MA)	This provider completes 3 years of training on treatment of common disorders.

Supplementary Figure 1: Sampling frames and sample size during pre-pandemic and pandemic period

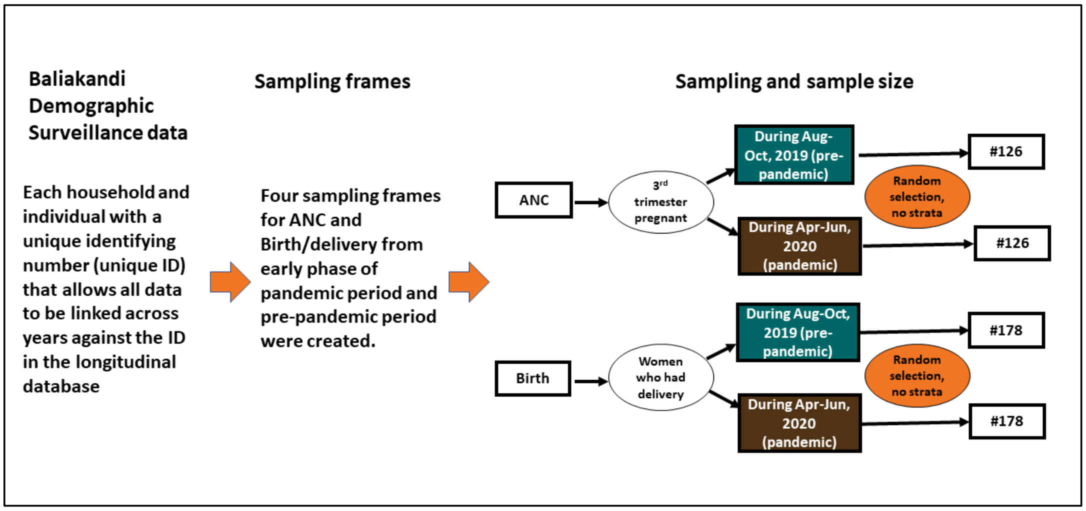

Supplementary Figure 2: Proportion of third trimester pregnant women who received ANC from a medically trained provider during pre-pandemic and pandemic period (N=226)

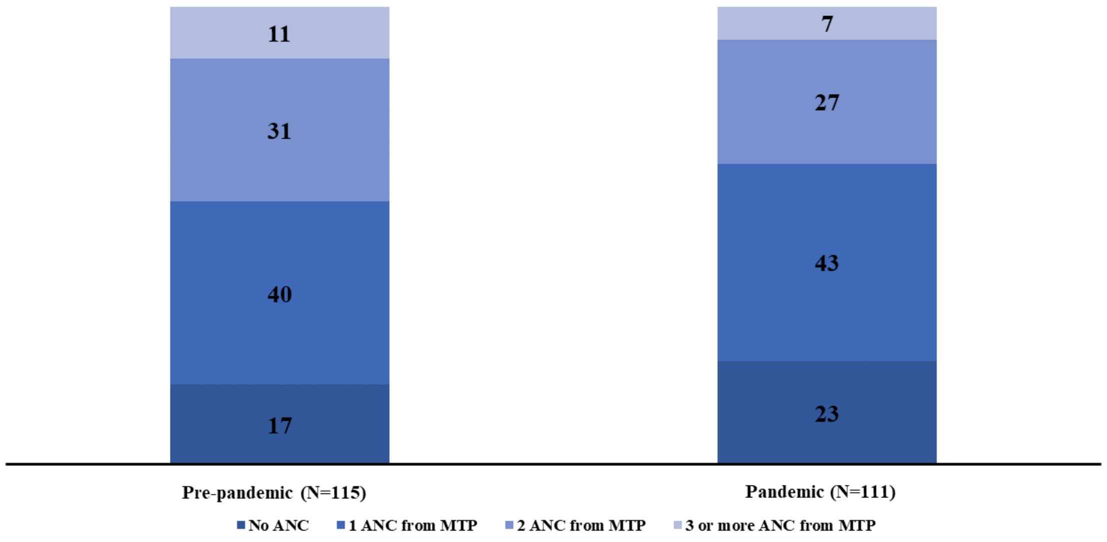

Supplementary Figure 3: Awareness and use of telemedicine services among the respondents (N=555)

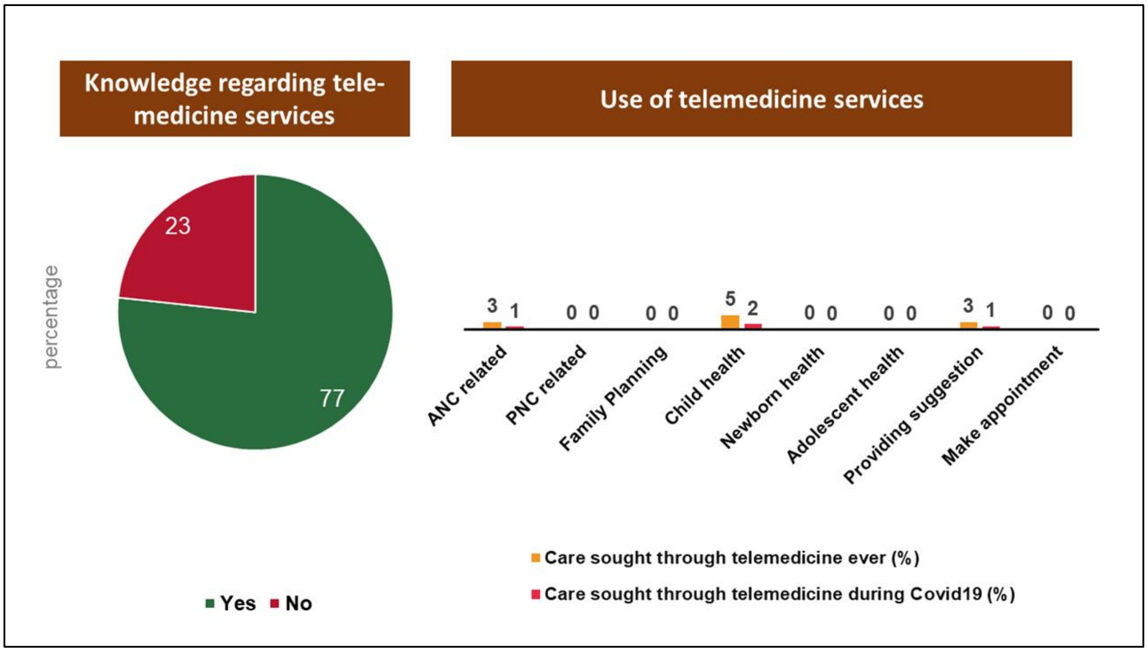

Supplement: Supplementary data [file bmjopen-2021-056951supp001.pdf]
