# Supplementary material for: Indirect effects of the early phase of the COVID-19 pandemic on the coverage of essential maternal and newborn health services in a rural subdistrict in Bangladesh: results from a cross-sectional household survey
Source: BMJ Open. 2022 Feb 3;12(2):e056951. doi: 10.1136/bmjopen-2021-056951 (PMC8814430; doi:10.1136/bmjopen-2021-056951)
Supplement: Author's manuscript [file bmjopen-2021-056951.draft_revisions.pdf]

BMJ Open

BMJ Open is committed to open peer review. As part of this commitment we make the peer review history of every article we publish publicly available.

When an article is published we post the peer reviewers' comments and the authors' responses online. We also post the versions of the paper that were used during peer review. These are the versions that the peer review comments apply to.

The versions of the paper that follow are the versions that were submitted during the peer review process. They are not the versions of record or the final published versions. They should not be cited or distributed as the published version of this manuscript.

BMJ Open is an open access journal and the full, final, typeset and author-corrected version of record of the manuscript is available on our site with no access controls, subscription charges or pay-per-view fees (http://bmjopen.bmj.com).

If you have any questions on BMJ Open's open peer review process please email
info.bmjopen@bmj.com

BMJ Open

Indirect effects of the lockdown measure due to the COVID-19 pandemic on the coverage of essential maternal and newborn health services in a rural sub-district in Bangladesh

Journal:	*BMJ Open*
Manuscript ID	bmjopen-2021-056951
Article Type:	Original research
Date Submitted by the Author:	31-Aug-2021
Complete List of Authors:	Mhajabin, Shema; International Centre for Diarrhoeal Disease Research Bangladesh, Maternal and Child Health<br>Hossain, Aniqa ; International Centre for Diarrhoeal Disease Research Bangladesh, biostatistics<br>Nusrat, Nowrin; International Centre for Diarrhoeal Disease Research Bangladesh<br>Jabeen, Sabrina; International Centre for Diarrhoeal Disease Research Bangladesh<br>Ameen, Shafiqul; International Centre for Diarrhoeal Disease Research Bangladesh<br>Banik, Goutom; International Centre for Diarrhoeal Disease Research Bangladesh, Maternal and Child Health Division ; International Centre for Diarrhoeal Disease Research Bangladesh,<br>Tahsina, Tazeen; International Centre for Diarrhoeal Disease Research Bangladesh, Maternal and Child Health Division<br>Ahmed , Anisuddin; ICDDRB<br>Sadeq-ur Rahman, Qazi; International Centre for Diarrhoeal Disease Research Bangladesh<br>Gurley, Emily; Johns Hopkins University<br>Bari, Sanwarul; International Centre for Diarrhoeal Disease Research Bangladesh<br>Chowdhury, Atique; ICDDRB<br>Arifeen, Shams E.; International Centre for Diarrhoeal Disease Research Bangladesh, Maternal and Child Health Division (MCHD)<br>Mehta, Rajesh; World Health Organization Regional Office for South-East Asia<br>Rahman, Ahmed ; International Centre for Diarrhoeal Disease Research Bangladesh; University of Edinburgh Division of Medical and Radiological Sciences
Keywords:	PUBLIC HEALTH, EPIDEMIOLOGY, COVID-19

I, the Submitting Author has the right to grant and does grant on behalf of all authors of the Work (as defined in the below author licence), an exclusive licence and/or a non-exclusive licence for contributions from authors who are: i) UK Crown employees; ii) where BMJ has agreed a CC-BY licence shall apply, and/or iii) in accordance with the terms applicable for US Federal Government officers or employees acting as part of their official duties; on a worldwide, perpetual, irrevocable, royalty-free basis to BMJ Publishing Group Ltd ("BMJ") its licensees and where the relevant Journal is co-owned by BMJ to the co-owners of the Journal, to publish the Work in this journal and any other BMJ products and to exploit all rights, as set out in our licence.

The Submitting Author accepts and understands that any supply made under these terms is made by BMJ to the Submitting Author unless you are acting as an employee on behalf of your employer or a postgraduate student of an affiliated institution which is paying any applicable article publishing charge ("APC") for Open Access articles. Where the Submitting Author wishes to make the Work available on an Open Access basis (and intends to pay the relevant APC), the terms of reuse of such Open Access shall be governed by a Creative Commons licence – details of these licences and which Creative Commons licence will apply to this Work are set out in our licence referred to above.

Other than as permitted in any relevant BMJ Author's Self Archiving Policies, I confirm this Work has not been accepted for publication elsewhere, is not being considered for publication elsewhere and does not duplicate material already published. I confirm all authors consent to publication of this Work and authorise the granting of this licence.

For peer review only

Indirect effects of the lockdown measure due to the COVID-19 pandemic on the coverage of essential maternal and newborn health services in a rural sub-district in Bangladesh

Shema Mhajabin[1], Aniqa Tasneem Hossain[1], Nowrin Nusrat[1], Sabrina Jabeen[1], Shafiqul Ameen[1], Goutom Banik[1], Tazeen Tahsina[1], Anisuddin Ahmed[1], Qazi Sadeq-ur Rahman[1], Emily S. Gurley[3], Sanwarul Bari[1], Md. Atique Iqbal Chowdhury[1], Shams El Arifeen[1≈], Rajesh Mehta[≈4], Ahmed Ehsanur Rahman[1&2≈*]

≈ Joint co-senior author

1- International Centre for Diarrhoeal Disease Research, Bangladesh (icddr,b)

2- University of Edinburgh

3- Johns Hopkins University

4- World Health Organization

*Corresponding Author: Dr Ahmed Ehsanur Rahman, icddr,b, Mohakhali, Dhaka-1212, Bangladesh, email: ehsanur@icddrb.org, Mobile Number: +8801727010050

Keywords: COVID-19 pandemic, Lockdown, rural area, essential services, maternal health, newborn health

Total word count, excluding title page, abstract, references, figures, and tables: 4300

For peer review only - http://bmjopen.bmj.com/site/about/guidelines.xhtml

ABSTRACT

Introduction: A shift of focus on the coronavirus disease 2019 (COVID-19) emergency responses from the essential health services may have resulted in the de-prioritization of such services, including maternal and newborn health (MNH). This paper presents the effect of the COVID-19 lockdown measures on the coverage of essential MNH services in a rural sub-district of Bangladesh.

Method: We conducted a cross-sectional household survey using random sampling to estimate the coverage of selected MNH services during the lockdown and pre-lockdown periods. The period of April 2020 to June 2020 was operationally defined as the lockdown period, and the period of August-October 2019 was defined as the pre-lockdown period. Two sample proportion tests were conducted to explore the difference in the coverage of essential MNH services between the lockdown and pre-lockdown periods. We reported any significant difference at $p<0.05$.

Findings: Among women who were third trimester pregnant during the lockdown period, 77% (95% CI: 70-85) received at least one antenatal care from a medically trained provider during the third trimester, compared to 83% (95% CI: 76-90) during the pre-lockdown period. Among women who gave birth during the lockdown period, 72% (95% CI: 66-79) were attended by a medically trained provider, compared to 63% (95% CI: 56-71) during the pre-lockdown period. Early initiation of breastfeeding was practiced among 38% (95% CI: 31-46) of the babies born during the lockdown period. It was 37% (95% CI: 29-44) during the pre-lockdown period.

Conclusion: Our study findings reported no significant changes in the overall coverage of MNH services before and during the lockdown in this rural area of Bangladesh. We inferred that the plausible reason could be that people access these services from private sector that was

For peer review only - http://bmjopen.bmj.com/site/about/guidelines.xhtml

not affected by the lockdown which does not comply with the pattern seen in the national HMIS.

Strengths and limitations of this study

- This paper provides a population level service coverage during the lockdown period which national level routine information system cannot capture.
- Additionally, this paper compares the service coverage of the essential MNH services before and during the lockdown to understand the effect of lockdown in rural area where usually service coverage is less compared to urban area.
- This study acknowledges the limitation of the cross-sectional design in inferring any causality and estimating the impact.
- This study might have issues with recall error since the participants were asked to report their care practice retrospectively. However, this study adapted the questionnaire based on the standard BDHS, BMMS, and MICS tools which are validated and globally accepted. To minimize the recall bias, this study considered a maximum recall of one-year and three months to estimating the coverage of essential MNH services. In contrast, DHS and MICS use a standard recall period of three years for estimating the coverage of relevant interventions.
- This study considered only 6 months of recall for assessing the coverage during the lockdown period. We recruited the data collectors from the local communities, organized face-to-face training and conducted household visits for interviewing the women, which add to the strength of the study and quality of data.

For peer review only - http://bmjopen.bmj.com/site/about/guidelines.xhtml

INTRODUCTION

The coronavirus disease 2019 (COVID-19), caused by the severe acute respiratory syndrome coronavirus 2 (SARS-CoV-2), was first reported in the port of Wuhan, China, in late December 2019. As of 2 June 2021, it has caused more than one hundred and seventy-one million confirmed cases across 219 countries and territories and taken more than three million lives, making it the deadliest pandemic since the Spanish flu in 1918 (1, 2). However, this is only the tip of the iceberg. Any pandemic negatively impacts the social, economic, and political landscape globally, regionally, and nationally (3-6). The unexpected health burden compels the policymakers to shift focus from routine healthcare and repurpose the existing resources to tackle the more ominous threats posed by the pandemic (7-9). It is, therefore, likely that the morbidity and mortality due to other causes may arise during a pandemic, especially in countries with limited resources (10, 11).

The risks of COVID-19 infection and its adverse clinical consequences are relatively low among children and the younger population, including women of reproductive ages (12, 13). However, they are still vulnerable to the indirect effects of the pandemic (14-17). Focusing on the emergency response may result in the de-prioritization of essential health services, including maternal and newborn health (MNH). The availability of essential MNH services may become severely compromised due to the repurposing of limited resources, overwhelming demand of other emergency services, healthcare workers' absence and deaths and reduced service hours or closure of health facilities (14, 18-20). Similarly, the access to and utilization of essential MNH services may be adversely affected by the hesitancy to seek care from health facilities due to the fear of contracting the virus and lack of reliable transportation due to the restrictive movement measures such as the lockdown (21, 22). A systematic review and meta-analysis of 40 studies reported that the global burden of maternal deaths, stillbirth and maternal depression have increased during the COVID-19 pandemic, with a high disparity between the

For peer review only - http://bmjopen.bmj.com/site/about/guidelines.xhtml

high-income and low- and middle-income country settings (23). Another study reported that the under-5 deaths increased by 45% per month and the maternal deaths have increased by 36% per month, across 118 countries due to the indirect effect the COVID-19 pandemic (14). According to United Nations report, COVID-19 may have contributed to an estimated 11,000 additional maternal deaths and 228,000 additional child deaths in South Asia in 2020 (15). Such adverse indirect effects of the COVID-19 pandemic may abrade the major gains in improving MNH achieved over the past few decades.

On March 08, 2020, Bangladesh declared the first confirmed case of COVID-19 and the first death was reported on 18 March 2020. Since then, Bangladesh has reported more than seven hundred and seventy thousand confirmed cases, including more than 11,000 deaths (24). The Government of Bangladesh imposed a national wide lockdown on 26 March 2020 for interrupting the viral transmission chain and flattening the epidemic curve (25). A sharp decline was observed in the utilization of essential MNH services during the lockdown period (March-May, 2020) in Bangladesh (26, 27). For example, the study reported that there was 41%, 52% and 56% decrease in April 2020 comparing to January 2020 for institutional normal deliveries in UHCs, DHs, and tertiary hospitals. However, this was based on the DHIS2 (routine health information system), which collects utilization data from public health facilities and some selected private facilities. Therefore, this might not reflect the change in the coverage of essential MNH services estimated with a true population-level denominator. This paper presents the effect of the lockdown measures due to the COVID-19 pandemic on the coverage of essential MNH services in Baliakandi, a rural sub-district in Bangladesh.

For peer review only - http://bmjopen.bmj.com/site/about/guidelines.xhtml

METHODS

Study design

We conducted a cross-sectional household survey to estimate the coverage of essential MNH services during the lockdown and pre-lockdown periods. Antenatal care (ANC) from a medically trained provider, birth attended by the medically trained provider, Essential Newborn Care (ENC), and Postnatal Care (PNC) from a medically trained provider within 72 hours of birth were identified as tracers for the essential MNH services. As the local administration had implemented pandemic measures the April-June 2020 period was operationally defined as the lockdown period. For identifying the pre-lockdown period (routine circumstances) we considered the following factors. China reported the first COVID-19 case in December 2019, and several global and national measures were taken between January and March 2020 and there may have been an impact on normal life and routine services. Before this, November 2019 – January 2020 period is the winter season in Bangladesh, and various cultural programs and family events are organized in this season, possibly leading to changes in the service use pattern. Hence, we considered August-October 2019 as the pre-lockdown period for comparison purposes.

Study setting

We conducted the study in a rural sub-district named Baliakandi, under the Rajbari district in Bangladesh. Since September 2017, icddr,b has been conducting health and demographic surveillance in this area (28). The surveillance covers 261 villages and a population of about 227,540 (in 2019). Through two monthly household visits, the surveillance data collectors collect information on births, deaths, marriages, divorces, and migrations. In addition to these vital events, they also collect information on pregnancy and birth outcomes.

For peer review only - http://bmjopen.bmj.com/site/about/guidelines.xhtml

Study participants and Outcome of interest

For assessing the effect of lockdown on the coverage of ANC, we randomly selected the women who gave birth in July 2020 (111 third trimester pregnant during the lockdown period) and those who gave birth in November 2019 (115 third trimester pregnant during the pre-lockdown period) as study participants. For estimating the coverage of birth attended by a medically trained provider, ENC and PNC, we randomly selected 163 women who gave birth in April-June 2020 (as the effect of lockdown period) and 166 women who gave birth in August-October 2019 (as the effect of the pre-lockdown period).

We calculated the sample size to detect a minimum effect size of 33 percentage point (relative) between the lockdown and the pre-lockdown periods at 80% power (two-sided) and 5% error probability. We used the coverage estimates (rural) reported in 2017-18 Bangladesh Demographic and Health Survey for the selected essential MNH services as proxies for the pre-lockdown status (29). The unadjusted sample size was adjusted for a non-response rate of 10% and the clustering effect with 1.10 design effect. Details regarding the sample size calculation for each of the essential MNH services is presented in (Supplementary table 1). We used the surveillance data to generate the sampling frame based on specific inclusion criteria. Then, we used simple random sampling to select the required number of participants from each category.

Data collection tools

We used an interviewer-administered structured questionnaire to interview the eligible respondents. The questions were primarily adopted from the Bangladesh Demographic and Health Survey (BDHS), Bangladesh Maternal Mortality Survey (BMMS), and Multiple Indicator Cluster Survey (MICS) (29-31). The questionnaire consisted of seven main sections: household information, socio-economic status, respondent's background, pregnancy, birth, and postnatal care, ENC, care-seeking during emergencies, telemedicine service, and COVID-19

For peer review only - http://bmjopen.bmj.com/site/about/guidelines.xhtml

related knowledge and experiences. We translated the questionnaire in Bangla and pre-tested it before finalization.

Training and data collection

We recruited fifteen data collectors and two field supervisors from the local communities who were familiar with the local context, culture, and dialect. The data collection team received 5-days of face-to-face training, which was followed by two days of field testing. The training was conducted by the study investigators and other master trainers with special expertise and experience in household surveys.

Data were collected between 7 November to 25 December 2020. The field supervisors revisited a random sample of 5% of households to ensure quality. In addition, the field supervisors conducted unscheduled visits to observe the interviews and monitor the quality of data collection. Weekly meetings were organized to solve any emerging issues related to data collection. All questionnaires were manually reviewed by the field supervisors and the field research managers before sending for data entry.

We maintained social distancing and followed the national infection prevention and control guidelines during training and data collection (32). All interviews were conducted outdoors. The data collection team received appropriate personal protective equipment such as masks, gloves, hand sanitizers, aprons, and face shields. Also, we distributed re-usable facemasks to the respondents to wear during the interviews.

Data analysis plan

We used the statistical software package STATA version 14.2 (StataCorp, College Station, TX) for data analysis (33). At first, descriptive statistics were used to report the background characteristics of the respondents. We used the principal component analysis to generate an asset score for each household (34). The asset scores were used to rank the households into

For peer review only - http://bmjopen.bmj.com/site/about/guidelines.xhtml

quintiles. We reported the coverage of essential MNH services for the lockdown and pre-lockdown periods separately with 95% confidence intervals (CI). The operational definitions of each of the indicators are presented in supplementary table 2, and the definition of providers are presented in supplementary table 3. Two sample proportion tests were conducted to explore the difference in the coverage of essential MNH services between the lockdown and pre-lockdown periods. We reported any significant difference at $p<0.05$.

Ethical approval and consent to participate

We obtained the ethical approval to conduct the study from the Institutional Review Board of icddr,b (PR-20085). Written and informed consent was sought from each study participant before the interview. In the case of respondents who cannot read or write, we obtained audio-recorded verbal informed consent.

RESULTS

We interviewed 115 women (non-response rate 9%) who were third-trimester pregnant during the pre-lockdown period and 111 women (non-response rate 12%) who were third-trimester pregnant during the lockdown period. We also interviewed 166 women (non-response rate 7%) who gave birth during the pre-lockdown period and 163 women (non-response rate 9%) who gave birth during the lockdown period. Table-1 describes the background characteristics of each group of respondents. Approximately one-third of the women were aged 20-24 years. Around half of them had incomplete secondary education (6-9 years of schooling), and one-fourth had complete secondary or higher education (10 or more years of schooling). Most of the respondents were Muslim. Approximately 40% of the women were primigravidae (first time pregnant). Less than 10% of them were engaged in any income-generating activities. No notable difference was observed in characteristics across the groups of respondents.

For peer review only - http://bmjopen.bmj.com/site/about/guidelines.xhtml

Table 1: Background characteristics of the respondents; presented in column percentage

Background characteristics	Women who were third trimester pregnant		Women who had a history of birth outcome	
	Pre-lockdown	Lockdown	Pre-lockdown	Lockdown
	N=115	N=111	N=166	N=163
	%	%	%	%
**Age**				
14 and below	0	1	0	0
15-19	23	24	14	22
20-24	31	36	33	37
25-29	21	25	26	22
30-34	19	13	22	12
35+	6	2	5	7
**Education**				
No education	2	1	5	3
Primary incomplete[1]	9	10	9	11
Primary complete[2]	7	9	12	11
Secondary incomplete[3]	56	48	52	52
Secondary complete or higher[4]	25	32	22	23
**Religion**				
Muslim	83	88	89	91
Hinduism/ Buddhism/ Christianity	17	12	11	9
**Parity**				
Primiparous	41	41	32	44
Multiparous	58	59	68	56
**Occupation**				
Housewife	96	95	95	90
Engaged in any income-generating activity	4	5	5	10
**Wealth quintile**				
Lowest	22	14	15	15
Second	23	19	24	24
Middle	25	23	25	23
Fourth	13	23	18	23
Highest	17	21	18	15
**Total**	100	100	100	100

[1]: 1-4 years of schooling
[2]: 5 years of schooling
[3]: 6-9 years of schooling
[4]: 10 or more years of schooling

For peer review only - http://bmjopen.bmj.com/site/about/guidelines.xhtml

Figure -1 summarises the coverage of essential MNH services during pre-lockdown and lockdown periods. Among women who were third-trimester pregnant during the lockdown period, 77% (95% CI: 70-85) received at least one ANC from a medically trained provider during the third trimester, which was 83% (95% CI: 76-90) during the pre-lockdown period. Similarly, among women who were third-trimester pregnant during the lockdown period, 81% (95% CI: 74-88) received at least one ANC from a health facility during the third trimester, which was 83% (95% CI: 76-90) during the pre-lockdown period. No notable difference was observed in coverage of ANC between the pre-lockdown and lockdown periods.

Among women who gave birth during the lockdown period, 72% (95% CI: 66-79) were attended by a medically trained provider. It was 63% (95% CI: 56-71) among those who gave birth during the pre-lockdown period. The coverage of facility-birth was 65% (95% CI: 58-72) during the lockdown period and 55% (95% CI: 47-62) during the pre-lockdown period. No notable difference was observed in coverage of births attended by a medically trained provider and facility-births between the pre-lockdown and lockdown periods. The coverage of at least one PNC from a medically trained provider was 58% (95% CI: 51-60) during the lockdown period and 52% (95% CI: 45-60) during the pre-lockdown period. Similarly, the coverage of at least one PNC from a health facility was 56% (95% CI: 48-63) during the lockdown period increased from 49% (CI: 42-57) during the pre-lockdown period.

Among babies born during the lockdown period, the coverage of clean cord cutting practice was 65% (95% CI: 58-72). It was 59% (95% CI: 52-67) among those who were born during the pre-lockdown period. 7.1% chlorhexidine was applied to 16% (95% CI: 10-22) of babies born during the lockdown period, which was 18% (95% CI: 12-24) during the pre-lockdown period. Early initiation of breastfeeding was practised among 38% (95% CI: 31-46) of the babies born during the lockdown period. It was 37% (95% CI: 29-44) during the pre-lockdown

For peer review only - http://bmjopen.bmj.com/site/about/guidelines.xhtml

period. No notable difference was observed in the coverage of ENC practices during the lockdown and the pre-lockdown periods.

Figure 1: Coverage of essential MNH services during the pre-lockdown and the lockdown periods, presented in percentage with 95% confidence intervals

Figure -2 illustrates the coverage of ANC received in the third trimester of pregnancy by months. Among the women who gave birth in July 2020 (i.e., third trimester pregnant during the lockdown period), 35% received at least one ANC from the medically trained provider, 36% from a health facility and 32% from a private health facility during the 7th month of pregnancy (April 2020). These estimates were significantly lower than that of the pre-lockdown period as among the women who gave birth in November 2020 (i.e., third trimester pregnant during the pre-lockdown period), 49% ($p<0.05$) received at least one ANC from the medically trained provider, 49% ($p<0.05$) from a health facility and 44% ($p<0.05$) from a private health facility during the 7th month of pregnancy (August 2019). No notable difference was observed in ANC coverage during the 8th and 9th month of pregnancy between the lockdown and pre-lockdown periods.

Figure 2: Coverage of ANC received in the third trimester during the pre-lockdown and the lockdown periods; presented in percentages by months of pregnancy

Among the women who were third-trimester pregnant during the lockdown period, 23% did not receive any ANC from a medically trained provider, 43% received one ANC from a medically trained provider, 27% received two or more ANC from a medically trained provider during that period (Supplementary figure 1). Among the women who were third-trimester pregnant during the pre-lockdown period, 17% did not receive any ANC from a medically trained provider, 40% received one ANC from a medically trained provider, 31% received two or more ANC from a medically trained provider during that period. We did not observe any

For peer review only - http://bmjopen.bmj.com/site/about/guidelines.xhtml

For peer review only

notable difference (statistically significant at $p<0.05$) in the above-mentioned distribution of ANC status between the lockdown and the pre-lockdown periods. The care-seeking for ANC using telemedicine services were very low and not different between lockdown and pre-lockdown period (Supplementary figure 2).

Figure-3 presents the type of health care provider from who the women received ANC during their third trimester of pregnancy. Among the women who were third-trimester pregnant during the lockdown period, 77% received ANC from a doctor, 11% from a nurse/midwife, 7% from an FWV and 0% from a CSBA. Among the women who were third-trimester pregnant during the pre-lockdown period, 80% received ANC from a doctor, 20% from a nurse/midwife, 11% from an FWV and 0% from a CSBA. No notable difference (statistically significant at $p<0.05$) was observed in the above-mentioned distribution of ANC providers between the lockdown and the pre-lockdown periods.

Figure 3: Type of provider from who ANC was received in the third trimester during the pre-lockdown and the lockdown periods; presented in percentages, multiple responses considered

Figure-4 presents the coverage of birth attended by a medically trained provider, birth in a health facility, birth in a private health facility and birth through C-sections. Among women who gave birth during the lockdown period, the coverage of birth attended by a medically trained provider was around 70% across all three months. Similarly, the coverage of facility-birth was more than 60% across all three months. Around 50% of birth occurred in a private health facility, and the rates were consistent across the three months. Approximately 50% of the birth were conducted through C-sections. The C-section rates were also consistent across three months during the lockdown period. We did not observe any notable difference in the coverage attended by a medically trained provider, birth in a health facility, birth in a private

For peer review only - http://bmjopen.bmj.com/site/about/guidelines.xhtml

health facility and birth through C-sections during the lockdown and pre-lockdown periods for any of the reporting months.

Figure 4: Coverage of births attended by a medically trained provider, birth in a health facility, birth in a private health facility and birth through C-sections during the pre-lockdown and lockdown periods; presented in percentages by calendar months

Figure-5 presents the type of health care providers attending the births which were conducted during the pre-lockdown and the lockdown period. Among the women who gave birth during the lockdown period, 53% were attended by a doctor, 71% by a nurse/midwife, 1% by an FWV and 0% by a CSBA. Among the women who gave birth during the pre-lockdown period, 49% were attended by a doctor, 62% from a nurse/midwife, 0% from an FWV and 2% from a CSBA. No notable difference (statistically significant at $p<0.05$) was observed in the distribution of providers attending births between the lockdown and the pre-lockdown periods.

Figure 5: Type of provider attending the births during the pre-lockdown and the lockdown periods; presented in percentages

Figure-6 shows coverage of PNC from a medically trained provider, PNC from a health facility, and PNC from a private health facility during the pre-lockdown and the lockdown periods. Among women who gave birth during the lockdown period, the coverage of PNC by a medically trained provider within 72 hours of birth was around 60% across all three months. Similarly, the coverage of PNC in a health facility facility-birth was around 55% across all three months. Approximately 45% received PNC in a private health facility, and the rates were consistent across the three months. We did not observe any notable difference in the coverage of PNC by a medically trained provider, PNC in a health facility, and PNC in a private health facility during the lockdown and pre-lockdown periods for any of the reporting months.

For peer review only - http://bmjopen.bmj.com/site/about/guidelines.xhtml

Figure 6: Coverage of PNC by a medically trained provider, PNC in a health facility, PNC in a private health facility among births occurred during the pre-lockdown and lockdown periods; presented in percentages by calendar months

Figure-7 presents the coverage of different component of ENC among the newborns during the pre-lockdown and the lockdown periods. Among babies born at the beginning of the lockdown period (April 2020), the coverage of early initiation of breastfeeding was 24%, which was much lower than that of the remaining months (May-June 2020) during the lockdown period and the pre-lockdown period. No notable difference was observed in the coverage of other components of ENC between the lockdown and the pre-lockdown periods by calendar months.

Figure 7: Coverage of different components of ENC during the pre-lockdown and lockdown periods; presented in percentages by calendar months

Patient and public involvement statement

The selected participants, women were asked to respond to the questionnaire during the household visits voluntarily. Their responses were noted during the data collection and were analysed anonymously at an aggregated level.

Throughout this study, patient involvement was not required. Patients were not invited to comment on the study design or were not consulted to interpret the results.

DISCUSSION

The health care-seeking experience for ANC, birth, PNC, and ENC during COVID-19 lockdown in rural Bangladesh was not found affected in this study. We found that the coverage of selective essential MNH services during the lockdown period was not different from that of the pre-lockdown period in this rural sub-district in Bangladesh. However, national level data from the routine health system of Bangladesh showed a substantial decline in the utilization of

For peer review only - http://bmjopen.bmj.com/site/about/guidelines.xhtml

essential MNH services (26). The possible explanations for such contradicting findings are summarized under two broad themes, i.e., measurement issues in the routine health information system and health systems and community responses, which are discussed below.

Health systems responses:

Several health systems initiatives taken by the Government of Bangladesh might have contributed to overcoming the initial shock of the pandemic and prevent a substantial decline in the coverage of key MNH services. In March 2020, the Government developed the Bangladesh Preparedness and Response Plan for COVID-19, which also emphasized the importance of maintaining essential health and nutrition services during the pandemic (37). After the initial focus on COVID-19-specific preparation and responses, several initiatives were taken to promote the availability and readiness of essential MNH services. In May 2202, the Ministry of Health, with technical inputs from the development partners and professional societies, developed the "National Guideline for providing essential Maternal, Newborn and Child Health Services in the context of COVID-19". The prime objective of this guideline was to inform the health care providers and facility- and district-level managers of the importance of providing essential MNCH services in the context of COVID-19 (38). It also provided specific guidance on continuing the essential services safely by maintaining physical distancing, using personal protective equipment, and promoting infection prevention and control practices. The Ministry of Health organized a series of online sessions to sensitize the facility- and district-level managers. Afterwards, face-to-face training was organized for the frontline health care workers responsible for providing MNH services. Then national-level policymakers organized virtual meetings with facility-, district- and regional-level managers to review the MNH utilization data in respective health facilities and discuss strategies for overcoming the bottlenecks, barriers, and operational challenges. Special initiatives were also taken for establishing functional triage, isolation unit for suspected COVID-19 patients, and

For peer review only - http://bmjopen.bmj.com/site/about/guidelines.xhtml

red zone for confirmed COVID-19 patients at referral facilities, which promoted a safer environment at the facility for receiving routine services and might have contributed to restoring the public confidence for availing essential MNH services. In June 2010, the Government of Bangladesh recruited and deployed 2000 doctors and 5000 nurses for COVID-19 response (39). The influx of additional human resources helped re-deploying the MNH services to their original duties and minimize the impact of COVID-19 on essential services. These national efforts might have positively influenced minimizing the disruption in the availability and provision of essential services in our study settings. According to the national routine health information system, there was little difference in the utilization of essential MNH services from public health facilities in the Baliakandi sub-district during the lockdown period (26). This is particularly applicable for the Upazila Health Complex, which is the primary referral point for all health centres in the study site. We also did not observe any notable difference in the utilization of MNH services from the district hospital during that period, which is the primary referral point for all the Upazila Health Complexes in that district (26). It indicates that most of the public health referral facilities continued providing routine services despite prioritizing the COVID-19 specific services. Since the referral facilities have more human resources than health centres, which have only one to two dedicated providers, it was easier for them to adopt an alternate approach for continuing essential MNH services.

Private sector coverage:

Bangladesh has a pluralistic health system constituted by the public sector, for-profit formal private sector and informal care providers (40, 41). Although the Government of Bangladesh has a large network of health infrastructure throughout the country, the majority of MNH services are provided by the poorly regulated for-profit private sector and unregulated informal care providers. According to 2017-18 BDHS, more than two-thirds of the ANC and facility-births took place in private health facilities (29). In the past decade, the coverage of births in

For peer review only - http://bmjopen.bmj.com/site/about/guidelines.xhtml

private health facilities has increased by four folds (29, 42). A similar trend was also observed for other essential MNH services such as ANC, PNC, and ENC. Although almost all the public health facilities report to DHIS2, very few private hospitals and private practitioners report to DHIS2. This disconnects between the larger market share of the private sector and disproportionately minor contribution in the DHIS2-based reporting system may explain our study findings. We did not observe any notable change in the coverage of essential MNH services during the lockdown period. In contrast, the DHIS2-based reporting system was indicating a substantial decline in service utilization nationally. In our study site, around 55% of births took place in the health facilities during the pre-lockdown period, of which about 80% were in private health facilities. The condition did not change during the lockdown period as 65% of births took place in health facilities, of which more than 80% were in private health facilities. We observed similar patterns for ANC and PNC services. In the rural sub-district where we conducted the study, there are three for-profit private health facilities that provide MNH services (28). In addition, there are 16 for-profit private facilities and two non-profit NGO clinics in the district headquarter, and many of them offer MNH services. Therefore, the prioritization of emergency services related to COVID-19 exclusively through the public health facilities may explain the initial decline in utilization of essential MNH services in those facilities, but the overwhelming majority in the market share the private sector and the continuation of the routine services may have contributed to sustaining the coverage essential MNH services in the early stage of the pandemic.

Ineffective lockdown:

The Government of Bangladesh imposed a nationwide restriction of movements in late March 2020 to interrupt the pathways of COVID-19 transmission (25, 43, 44). However, the Government declared it as 'general leave' instead of lockdown, which undermined the importance of adhering to the recommended measures among the mass population. At the

For peer review only - http://bmjopen.bmj.com/site/about/guidelines.xhtml

beginning of the lockdown, all the government and non-government offices, factories and academic institutes were asked to shut down. A large proportion of city dwellers travelled back to their village homes, maintaining very little social distancing and other infection prevention and control measures (43). After a couple of weeks, some government offices, and factories, including all readymade garment factories, were allowed to resume their operations, forcing many people to return to their workplace. Hence, the restrictions regarding inter-district and within-district movements gradually weaned off, and the public transportation options slowly resumed back with some infection prevention and control regulations (25). Moreover, the lockdown measures were less coercively imposed in rural settings as the transportation services were reasonably available, allowing people to move within the sub-district and to the district headquarter when required. In our study, less than 5% of the respondents mentioned lack of transportation as a barrier to care-seeking, and less than 10% reported spending extra money for the vehicle rent while receiving essential MNH services during the lockdown period. Based on a national survey, around two-thirds of the pregnant women in rural settings can reach a private health facility or provider within five-kilometre of travel distance (29). It implies that people could avail the essential MNH services from nearby private health facilities when the services were temporarily disrupted in public health facilities in the early stage of the pandemic.

Community coping strategy and resilience:

Bangladesh is one of the most vulnerable countries to climate variability and fluctuation (45, 46). A quarter of the land in Bangladesh is barely above sea level and is susceptible to the effects of sea water rise and increased salinity due to climate change. Bangladesh also experiences other forms of natural disasters like flood and cyclones quite frequently (47). The people in Bangladesh have learnt to cope with the emergencies and shows resilience by focusing on rebuilding after the initial shock, mostly through local-level initiatives (48, 49). Awareness regarding the importance of receiving MNH services from appropriate places and

For peer review only - http://bmjopen.bmj.com/site/about/guidelines.xhtml

providers has increased substantially and consistently over the past couple of decades, even in the rural context. Hence the awareness and the resilience nature of local communities might have contributed to finding ingenious ways and coping strategies to avail the essential MNH service during the pandemic overcoming the challenges imposed by the lockdown.

Measurement issues:

As mentioned earlier, there are limitations in estimating the coverage of health services through the routine health information system in Bangladesh. The Government of Bangladesh has made a substantial investment in strengthening the routine health information system by introducing DHIS2 in 2009 (40). All the public health facilities and few private health facilities report their health service use data to DHIS2. Although MNH is a priority in the DHIS2-based reporting system in Bangladesh, the system's reach is not enough to accurately capture population-level denominators such as live births, which are essential for measuring the coverage of MNH services. In 2019, DHIS2 reported a total of 931,723 live births in Bangladesh (26). Based on the crude birth rate reported in 2017-18 BDHS, the size of the annual birth cohort is more than 3 million in Bangladesh (29). Similarly, DHIS2 reported that around 655,542 women received 4 ANC from the reporting facilities in 2019 (26). However, an estimated 1,410,000 (47% of 3 million births) women received four or more ANC based on the national survey estimate (29). Moreover, there are issues with the accuracy and quality of reporting in the routine health information systems, including DHIS2, particularly in low- and middle-income country settings (50-53). Due to these gaps in accurately capturing the denominators and numerators through DHIS2-based reporting system, the decline observed in the utilization of essential

For peer review only

For peer review only - http://bmjopen.bmj.com/site/about/guidelines.xhtml

MNH services in public health facilities in the early stage of the pandemic may not reflect the true change in the coverage of relevant services.

Secondly, there were gaps and delays in the documentation and reporting of routine services in the initial phase of the pandemic, particularly during the lockdown period due to the reshaping of the health care providers' responsibilities and health systems responses. The Government of Bangladesh prepared the National Preparedness and Response Plan for the pandemic and repurposed the existing infrastructure, human resource, and logistics to prevent and treat COVID-19 (37). Initially, triage was introduced in all public health facilities and some hospitals were dedicated to treating COVID-19. Additionally, a COVID-19 dashboard has been developed and is updated daily (24). This portal displays data on the number of laboratories testing for COVID-19 and the number of health facilities treating COVID-19 patient. The dashboard also displays information on the number of tests performed, number of cases identified, number of people in quarantine and isolation, and number of deaths daily. It also provides an overview of the stock availability of COVID-19 related logistics of all health facilities. The frontline health care workers, facility and district level managers and national policymakers prioritized capturing and reporting of COVID-19 related data to monitor the relevant implementation and response status. Hence, the introduction and emphasis on COVID-19 related services, documentation and reporting might have overwhelmed the health care providers and the health systems resulting in the initial gaps and delays of reporting other routine services, including MNH. For example, we accessed DHIS2 in June 2020 and observed a sharp decline in the utilization of essential MNH services from public health facilities in March-May 2020 (26). We accessed DHIS2 in September 2020 and found that the service utilization numbers were updated for the same time March-May 2020, resulting in a much smaller decline than the pre-COVID-19 period. Therefore, the true impact on the coverage of

For peer review only - http://bmjopen.bmj.com/site/about/guidelines.xhtml

essential MNH services might be less than what was originally observed based on DHIS2 utilization data in the early phase of the pandemic.

Conclusion

Our study findings reported no significant changes in the overall coverage of MNH services in rural area of Bangladesh. Moreover, the changes were not even significant for monthly comparison during the lockdown and pre-lockdown periods. It highlights the importance of expanding and reinforcing the routine health information system to cover the information from the private health facilities and community services for comprehensive monitoring of essential services, particularly during the emergency response. The results also reveal the strength of local level coping strategy and coping strategy to overcome the immediate shock.

For peer review only

For peer review only - http://bmjopen.bmj.com/site/about/guidelines.xhtml

REFERENCE

1. Worldometers. COVID-19 CORONAVIRUS PANDEMIC 2021 [Available from: https://www.worldometers.info/coronavirus/.
2. (WHO) WHO. WHO Coronavirus (COVID-19) Dashboard 2021 [Available from: https://covid19.who.int/.
3. Davies SE. National security and pandemics. UN Chronicle. 2013;50(2):20-4.
4. Drake TL, Chalabi Z, Coker R. Cost-effectiveness analysis of pandemic influenza preparedness: what's missing? Bulletin of the World Health Organization. 2012;90:940-1.
5. Tisdell CA. Economic, social and political issues raised by the COVID-19 pandemic. Economic analysis and policy. 2020;68:17-28.
6. Qiu W, Rutherford S, Mao A, Chu C. The pandemic and its impacts. Health, culture and society. 2017;9:1-11.
7. Chowdhury A. Repurposing innovations: Learning from Bangladesh's rapid response to the pandemic'. 2020.
8. Ahmed T, Rahman AE, Amole TG, Galadanci H, Matjila M, Soma-Pillay P, et al. The effect of COVID-19 on maternal newborn and child health (MNCH) services in Bangladesh, Nigeria and South Africa: call for a contextualised pandemic response in LMICs. International journal for equity in health. 2021;20(1):1-6.
9. Her M. Repurposing and reshaping of hospitals during the COVID-19 outbreak in South Korea. One Health. 2020;10:100137.
10. Wilhelm JA, Helleringer S. Utilization of non-Ebola health care services during Ebola outbreaks: a systematic review and meta-analysis. J Glob Health. 2019;9(1).
11. Jones SA, Gopalakrishnan S, Ameh CA, White S, van den Broek NR. 'Women and babies are dying but not of Ebola': the effect of the Ebola virus epidemic on the availability, uptake and outcomes of maternal and newborn health services in Sierra Leone. BMJ global health. 2016;1(3):e000065.
12. Sinha IP, Harwood R, Semple MG, Hawcutt DB, Thursfield R, Narayan O, et al. COVID-19 infection in children. The Lancet Respiratory Medicine. 2020.
13. zazhi ZlxbxzzZl. The epidemiological characteristics of an outbreak of 2019 novel coronavirus diseases (COVID-19) in China. Novel, Coronavirus Pneumonia Emergency Response Epidemiology. 2020;41(2):145.

For peer review only - http://bmjopen.bmj.com/site/about/guidelines.xhtml

14. Roberton T, Carter ED, Chou VB, Stegmuller AR, Jackson BD, Tam Y, et al. Early estimates of the indirect effects of the COVID-19 pandemic on maternal and child mortality in low-income and middle-income countries: a modelling study. The Lancet Global Health. 2020;8(7):e901-e8.
15. UNICEF. Direct and indirect effects of the COVID-19 pandemic and response in South Asia. UNICEF; 2021 March.
16. Busch-Hallen J, Walters D, Rowe S, Chowdhury A, Arabi M. Impact of COVID-19 on maternal and child health. The Lancet Global Health. 2020;8(10):e1257.
17. Ilerioluwa Oke G, Ebuka Elebesunu E, Ihekweazu V. Impact of COVID-19 Pandemic on Maternal and Child Health. Modern Care Journal. 2020;17(4).
18. Elston JW, Cartwright C, Ndumbi P, Wright J. The health impact of the 2014-15 Ebola outbreak. Public health. 2017;143:60-70.
19. Semaan A, Audet C, Huysmans E, Afolabi B, Assarag B, Banke-Thomas A, et al. Voices from the frontline: findings from a thematic analysis of a rapid online global survey of maternal and newborn health professionals facing the COVID-19 pandemic. BMJ Glob Health. 2020;5(6).
20. Hassanian-Moghaddam H, Zamani N, Kolahi A-A. COVID-19 pandemic, healthcare providers' contamination and death: an international view. Critical Care. 2020;24(1):208.
21. Pant S, Koirala S, Subedi M. Access to maternal health services during COVID-19. Europasian Journal of Medical Sciences. 2020;2(2):48-52.
22. Temesgen K, Wakgari N, Debelo BT, Tafa B, Alemu G, Wondimu F, et al. Maternal health care services utilization amidstCOVID-19 pandemic in West Shoa zone, central Ethiopia. PloS one. 2021;16(3):e0249214.
23. Chmielewska B, Barratt I, Townsend R, Kalafat E, van der Meulen J, Gurol-Urganci I, et al. Effects of the COVID-19 pandemic on maternal and perinatal outcomes: a systematic review and meta-analysis. The Lancet Global Health. 2021.
24. Director Genereal of Health Services B. Coronavirus COVID-19 Dashboard, 2020 2020 [Available from: http://dashboard.dghs.gov.bd/webportal/pages/covid19.php.
25. Shammi M, Bodrud-Doza M, Islam ARMT, Rahman MM. Strategic assessment of COVID-19 pandemic in Bangladesh: comparative lockdown scenario analysis, public perception, and management for sustainability. Environment, Development and Sustainability. 2020:1-44.
26. Director Genereal of Health Services B. DHIS2 Central Database (Upazila level and Above) 2021 [Available from: https://dghs.gov.bd/index.php/en/component/content/article?id=456.
27. Wangmo S, Sarkar S, Islam T, Rahman MH, Landry M. Maintaining essential health services during the pandemic in Bangladesh: the role of primary health care supported by routine health information system. WHO South-East Asia Journal of Public Health. 2021;10(3):93.
28. CHAMPS. CHAMPS in Bangladesh 2021 [cited 2021. Available from: https://champshealth.org/sites-bangladesh/.
29. National Institute of Population Research and Training (NIPORT), ICF International. Bangladesh Demographic and Health Survey 2017-18: Key indicators. Dhaka, Bangladesh, and Rockville, Maryland, USA; 2019.
30. National Institute of Population Research and Training (NIPORT), International Centre for Diarrhoeal Disease Research Bangladesh (icddr b, MEASURE Evaluation. Bangladesh Maternal Mortality and Health Care Survey 2016: Preliminary Report. Dhaka, Bangladesh and Chapel Hill, NC, USA; 2017.
31. Unicef. MULTIPLE INDICATOR CLUSTER SURVEY 2016. NBS, UNICEF; 2018.

For peer review only - http://bmjopen.bmj.com/site/about/guidelines.xhtml

32. Directorate General of Health Services (DGHS) MoHaFWM, Bangladesh. NATIONAL GUIDELINE ON INFECTION PREVENTION AND CONTROL IN HEALTHCARE SETTINGS WITH ADDITIONAL MEASURES FOR COVID-19. Directorate General of Health Services (DGHS), Ministry of Health and Family Welfare (MOHFW), Bangladesh; 2020 29 August, 2020.
33. StataCorp. STATA version 14.2 2021 [updated 2021; cited 2021. Available from: https://www.stata.com/.
34. Wold S, Esbensen K, Geladi P. Principal component analysis. Chemometrics and intelligent laboratory systems. 1987;2(1-3):37-52.
35. World Health Organization. Statement on the second meeting of the International Health Regulations (2005) Emergency Committee regarding the outbreak of novel coronavirus (2019-nCoV) 2020 [Available from: https://www.who.int/news-room/detail/30-01-2020-statement-on-the-second-meeting-of-the-international-health-regulations-(2005)-emergency-committee-regarding-the-outbreak-of-novel-coronavirus-(2019-ncov).
36. World Health Organization. Coronavirus disease (COVID-19) Pandemic 2020 [Available from: https://www.who.int/emergencies/diseases/novel-coronavirus-2019.
37. Ministry of Health and Family Welfare (MoH&FW). National Preparedness and Response Plan for COVID-19 Bangladesh. Directorate General of Health Services, Health Service Division, Ministry of Health and Family Welfare, Bangladesh; 2020.
38. MNCAH D. National Guideline for providing essential Maternal, Newborn and Child Health Services in the context of COVID-19. May 2020.
39. bdnews24.com. Bangladesh moves to recruit 2,000 doctors, 5,000 nurses amid virus outbreak. May 2020.
40. Khan MAH, Azad AK, de Oliveira Cruz V. Bangladesh's digital health journey: reflections on a decade of quiet revolution. WHO South-East Asia journal of public health. 2019;8(2):71-6.
41. Organization WH. Bangladesh health system review: Manila: WHO Regional Office for the Western Pacific; 2015.
42. National Institute of Population Research and Training (NIPORT), Mitra and associates, Macro International. Bangladesh Demographic and Health Survey 2007. Dhaka, Bangladesh, and Rockville, Maryland, USA; 2007 March, 2009.
43. Anwar S, Nasrullah M, Hosen MJ. COVID-19 and Bangladesh: Challenges and how to address them. Frontiers in public health. 2020;8.
44. Shammi M, Bodrud-Doza M, Islam ARMT, Rahman MM. COVID-19 pandemic, socioeconomic crisis and human stress in resource-limited settings: A case from Bangladesh. Heliyon. 2020;6(5):e04063.
45. Naser MM, Swapan MSH, Ahsan R, Afroz T, Ahmed S. Climate change, migration and human rights in Bangladesh: Perspectives on governance. Asia Pacific Viewpoint. 2019;60(2):175-90.
46. Shahid S. Probable impacts of climate change on public health in Bangladesh. Asia Pacific Journal of Public Health. 2010;22(3):310-9.
47. Rahman R, Salehin M. Flood risks and reduction approaches in Bangladesh. Disaster risk reduction approaches in Bangladesh: Springer; 2013. p. 65-90.
48. Parvin GA, Shimi AC, Shaw R, Biswas C. Flood in a changing climate: The impact on livelihood and how the rural poor cope in Bangladesh. Climate. 2016;4(4):60.
49. Younus MAF, Kabir MA. Climate change vulnerability assessment and adaptation of Bangladesh: Mechanisms, notions and solutions. Sustainability. 2018;10(11):4286.
50. Day LT, Rahman QS-u, Rahman AE, Salim N, Ashish K, Ruysen H, et al. Assessment of the validity of the measurement of newborn and maternal health-care coverage

For peer review only - http://bmjopen.bmj.com/site/about/guidelines.xhtml

in hospitals (EN-BIRTH): an observational study. The Lancet Global Health. 2021;9(3):e267-e79.
51. Tahsina T, Hossain AT, Ruysen H, Rahman AE, Day LT, Peven K, et al. Immediate newborn care and breastfeeding: EN-BIRTH multi-country validation study. BMC pregnancy and childbirth. 2021;21(1):1-17.
52. Day LT, Gore-Langton GR, Rahman AE, Basnet O, Shabani J, Tahsina T, et al. Labour and delivery ward register data availability, quality, and utility-Every Newborn-birth indicators research tracking in hospitals (EN-BIRTH) study baseline analysis in three countries. BMC health services research. 2020;20(1):1-14.
53. Bhattacharya AA, Umar N, Audu A, Felix H, Allen E, Schellenberg JR, et al. Quality of routine facility data for monitoring priority maternal and newborn indicators in DHIS2: A case study from Gombe State, Nigeria. PloS one. 2019;14(1):e0211265.

For peer review only

For peer review only - http://bmjopen.bmj.com/site/about/guidelines.xhtml

AUTHORS' CONTRIBUTION

SM and AER are the co-principal investigator of this study, and they conceptualised the study. SM have developed the first draft of the manuscript and incorporated the feedback of the authors. SM, ATH, NN, and AER have conducted the data analysis and interpretated the findings. All the authors have contributed significantly to design the study, develop the data collection materials, and prepare the final draft of the manuscript. They have reviewed and provided feedback to the manuscript. AER, SEA, and RM have led the process as the senior authors.

FUNDING STATEMENT

The study was supported by the World Health Organization (WHO) under the grant of BGT-0031/2020.

COMPETING INTERESTS STATEMENT

There is no competing or conflicting interest.

ACKNOWLEDGEMENT

The authors would like to acknowledge the kind support of the Baliakandi demographic surveillance site which is supported by the CHAMPS project by Gates Foundation by Emory University throughout the data collection. icddr,b is also grateful to the Governments of Bangladesh, Canada, Sweden, and the UK for providing unrestricted/institutional support.

For peer review only - http://bmjopen.bmj.com/site/about/guidelines.xhtml

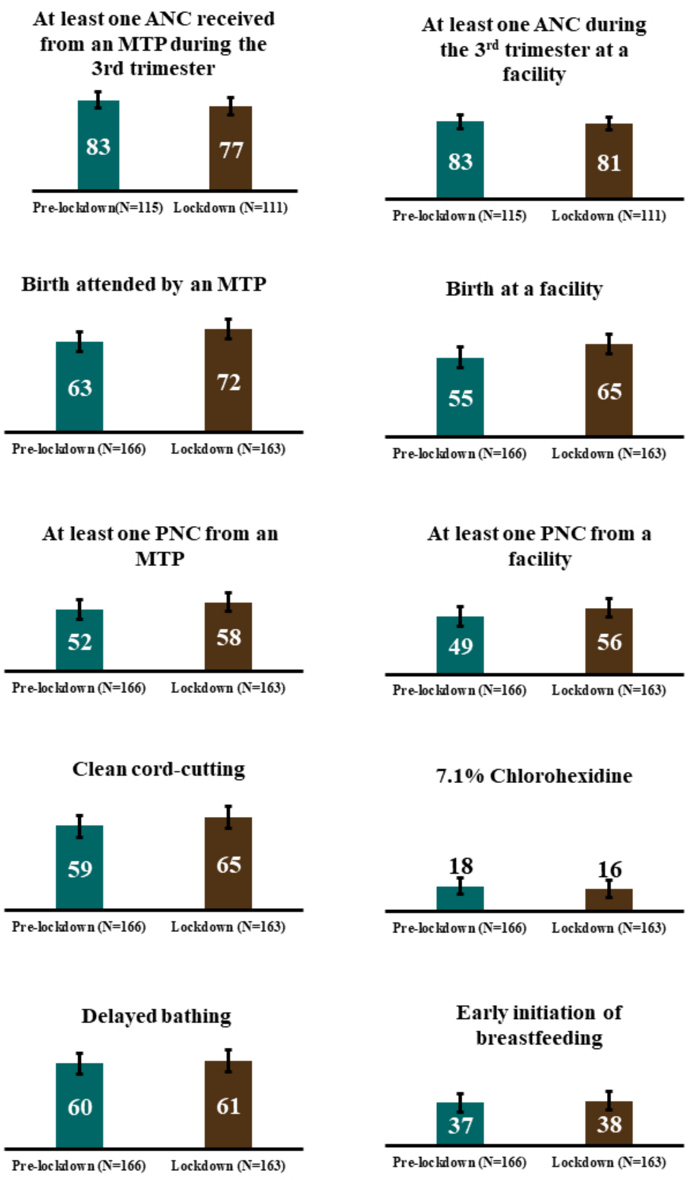

Coverage of essential MNH services during the pre-lockdown and the lockdown periods, presented in percentage with 95% confidence intervals

481x695mm (38 x 38 DPI)

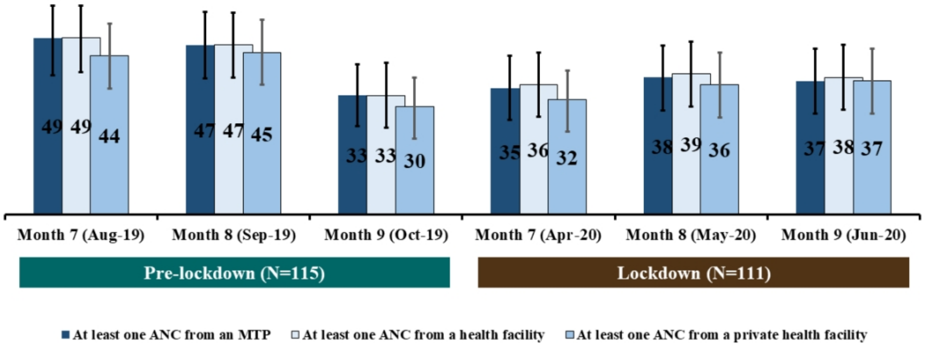

Coverage of ANC received in the third trimester during the pre-lockdown and the lockdown periods; presented in percentages by months of pregnancy

855x481mm (38 x 38 DPI)

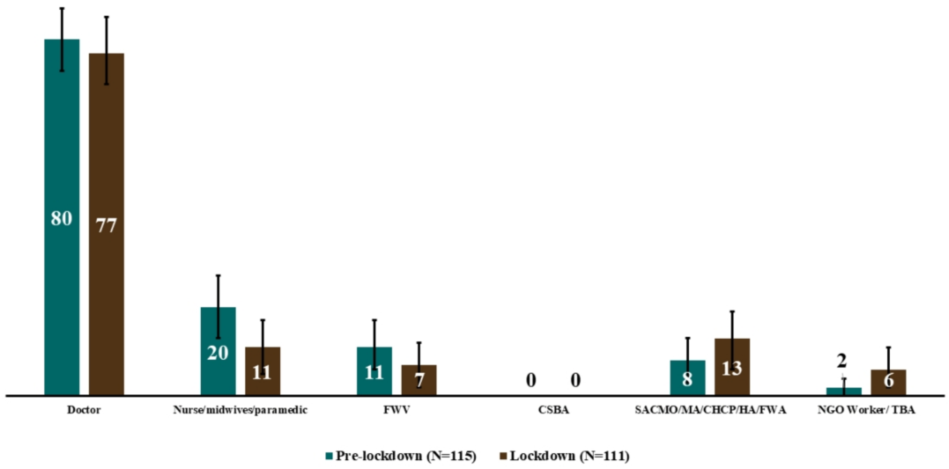

Type of provider from who ANC was received in the third trimester during the pre-lockdown and the lockdown periods; presented in percentages, multiple responses considered

855x481mm (38 x 38 DPI)

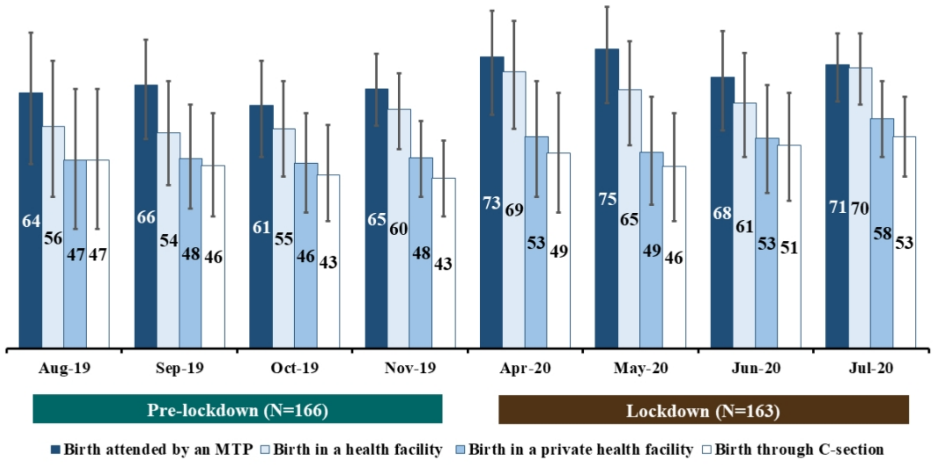

Coverage of births attended by a medically trained provider, birth in a health facility, birth in a private health facility and birth through C-sections during the pre-lockdown and lockdown periods; presented in percentages by calendar months

855x481mm (38 x 38 DPI)

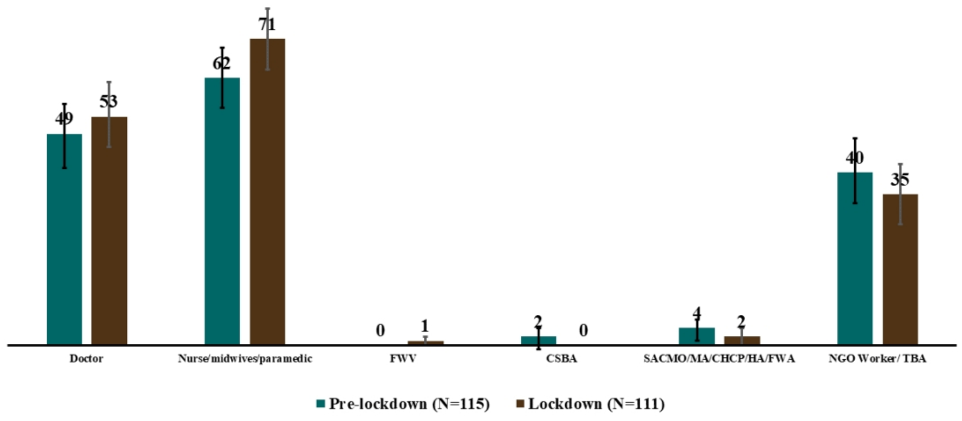

Type of provider attending the births during the pre-lockdown and the lockdown periods; presented in percentages

855x481mm (38 x 38 DPI)

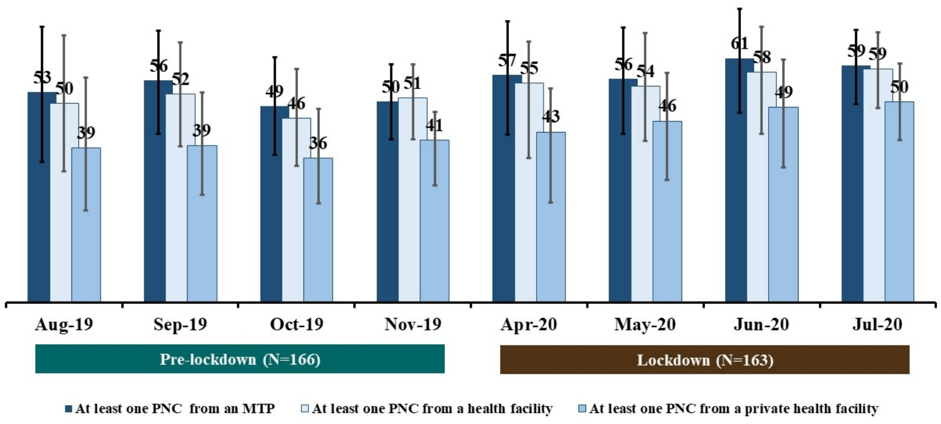

Coverage of PNC by a medically trained provider, PNC in a health facility, PNC in a private health facility among births occurred during the pre-lockdown and lockdown periods; presented in percentages by calendar months

338x190mm (96 x 96 DPI)

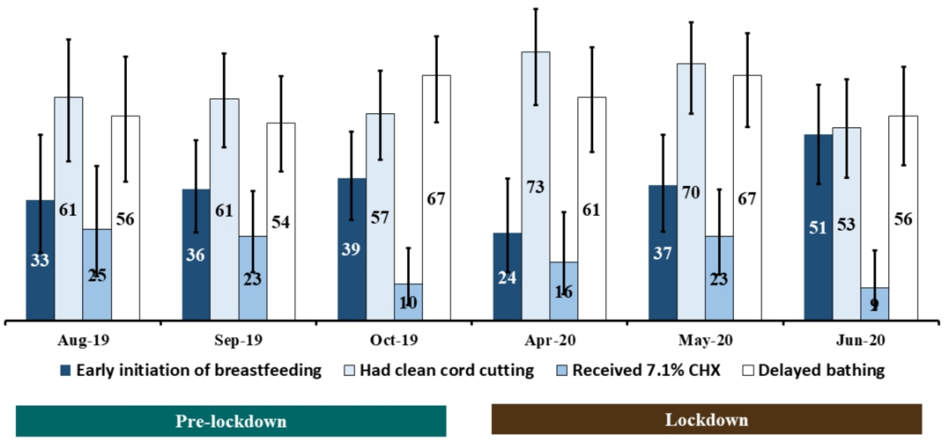

Coverage of different components of ENC during the pre-lockdown and lockdown periods; presented in percentages by calendar months

855x481mm (38 x 38 DPI)

Supplementary Table 1: Summary of indicator selected for each target population with required sample size

**Target population**	**Indicator**	**Baseline (pre-lockdown period)**	**Endline (Lockdown period)**	**Required Sample Size**
Women who were third trimester pregnant during the COVID-19' lockdown' period and the selected pre-lockdown period in the preceding year	At least one ANC from MTP<br>At least one ANC from facility	60%	30%	126
Women who had a history of birth during the COVID-19' lockdown' period and the selected pre-lockdown period in the preceding year	Birth attended by an MTP<br>Birth in a health facility<br>At least one PNC from an MTP within 48 hours of birth<br>Newborns received essential newborn care (ENC)	50%	33%	178

For peer review only

Supplementary Table 2: Definition of selected indicator

Selected indicators	Definitions
Indicator 1	Proportion of women who were third trimester pregnant during the COVID-19' lockdown' period and the pre-lockdown period in the preceding year receiving at least one ANC from MTP
Indicator 2	Proportion of women who were third trimester pregnant during the COVID-19' lockdown' period and the pre-lockdown period in the preceding year receiving at least one ANC from facility
Indicator 3	Proportion of women giving birth during COVID-19' lockdown' period and the corresponding period in the preceding year were attended by an MTP
Indicator 4	Proportion of women giving birth during COVID-19' lockdown' period and the corresponding period in the preceding year at a facility
Indicator 5	Proportion of newborn giving birth during the COVID-19' lockdown' period and the pre-lockdown period in the preceding year receiving PNC from an MTP within 48 hours of birth
Indicator 6	Proportion of newborns having born during the COVID-19' lockdown' period and the pre-lockdown period in the preceding year received drying
Indicator 7	Proportion of newborns having born during the COVID-19' lockdown' period and the pre-lockdown period in the preceding year had clean cord cutting
Indicator 8	Proportion of newborns having born during the COVID-19' lockdown' period and the pre-lockdown period in the preceding year received 7.1% chlorohexidine
Indicator 9	Proportion of newborns having born during the COVID-19' lockdown' period and the pre-lockdown period in the preceding year had delayed bathing
Indicator 10	Proportion of newborns having born during the COVID-19' lockdown' period and the pre-lockdown period in the preceding year had early initiation of breastfeeding

For peer review only

Supplementary Table 3: Definition of providers and facilities

Type of providers		Definition
Medically trained provider (MTP)	Doctors (General physicians)	This provider has five years of training on medicine, surgery, gynaecology, and obstetrics with one-year internship in medicine, surgery, gynaecology, and obstetrics wards. This provider is qualified to provide ANC, Basic emergency obstetric services (BEOC), PNC, menstrual regulation (MR), abortion care (AC), family planning services (FPS).
	Nurse	This provider completes 3-4 years of nursing course with 6 months dedicated to obstetrician training. This provider is qualified to provide ANC, BEOC, PNC, MR, AC, FPS.
	Midwife	This provider completes one year of midwifery training with 6 months dedicated to obstetrician training. This provider is qualified to provide ANC, BEOC, PNC, MR, AC, FPS.
	Family welfare visitor (FWV)	This provider completes 18 months of training on maternal and child health, family planning, and contraception. This provider is qualified to provide facility-based ANC, BEOC, PNC, MR, AC, FPS.
	Community Skilled Birth Attendant (CSBA)	This provider completes 6 months of training on BEOC and ENC. This provider is qualified to provide community-based ANC, BEOC, PNC, FPS.
Non-medically trained provider (non-MTP)	Family Welfare Assistant (FWA)	This provider completes 30 days of training on family planning. This provider is qualified to provide community-based FPS.
	Health assistant (HA)	This provider completes 3 months of training on limited preventive and curative care, immunization. This provider is qualified to provide community-based immunization of mother and children.
	Sub Assistant Community Medical Officer (SACMO)	This provider does not have any obstetrician training.
	Community Health Care Provider (CHCP)	This provider does not have any obstetrician training.
	Medical assistants (MA)	This provider completes 3 years of training on treatment of common disorders.

For peer review only

Supplementary Figure 1: Proportion of third trimester pregnant women who received ANC from MTP during pre-lockdown and lockdown period (N=226)

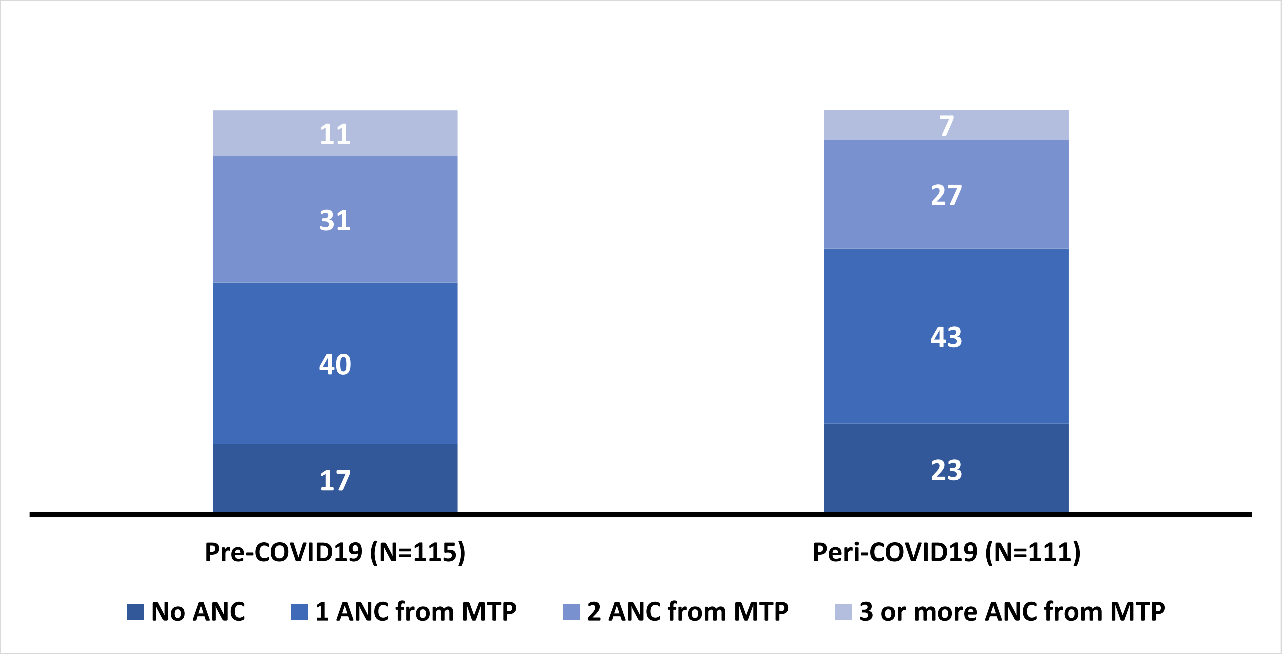

For peer review only

Supplementary Figure 2: Awareness and use of telemedicine services among the respondents (N=555)

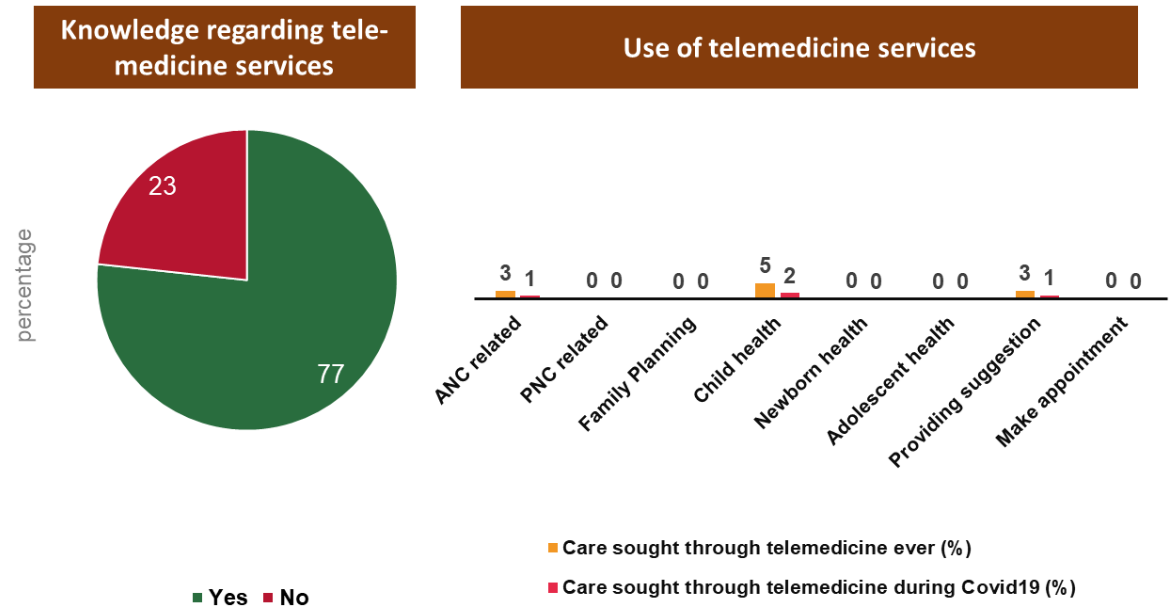

eview only

BMJ Open

Indirect effects of the early phase of the COVID-19 pandemic on the coverage of essential maternal and newborn health services in a rural sub-district in Bangladesh: results from a cross-sectional household survey

Journal:	*BMJ Open*
Manuscript ID	bmjopen-2021-056951.R1
Article Type:	Original research
Date Submitted by the Author:	10-Nov-2021
Complete List of Authors:	Mhajabin, Shema; International Centre for Diarrhoeal Disease Research Bangladesh, Maternal and Child Health<br>Hossain, Aniqa ; International Centre for Diarrhoeal Disease Research Bangladesh, biostatistics<br>Nusrat, Nowrin; International Centre for Diarrhoeal Disease Research Bangladesh<br>Jabeen, Sabrina; International Centre for Diarrhoeal Disease Research Bangladesh<br>Ameen, Shafiqul; International Centre for Diarrhoeal Disease Research Bangladesh<br>Banik, Goutom; International Centre for Diarrhoeal Disease Research Bangladesh, Maternal and Child Health Division ; International Centre for Diarrhoeal Disease Research Bangladesh,<br>Tahsina, Tazeen; International Centre for Diarrhoeal Disease Research Bangladesh, Maternal and Child Health Division<br>Ahmed , Anisuddin; ICDDRB<br>Sadeq-ur Rahman, Qazi; International Centre for Diarrhoeal Disease Research Bangladesh<br>Gurley, Emily; Johns Hopkins University<br>Bari, Sanwarul; International Centre for Diarrhoeal Disease Research Bangladesh<br>Chowdhury, Atique; ICDDRB<br>Arifeen, Shams E.; International Centre for Diarrhoeal Disease Research Bangladesh, Maternal and Child Health Division (MCHD)<br>Mehta, Rajesh; World Health Organization Regional Office for South-East Asia<br>Rahman, Ahmed ; International Centre for Diarrhoeal Disease Research Bangladesh; University of Edinburgh Division of Medical and Radiological Sciences
**Primary Subject Heading**:	Public health
Secondary Subject Heading:	Epidemiology, Evidence based practice, Global health
Keywords:	PUBLIC HEALTH, EPIDEMIOLOGY, COVID-19

I, the Submitting Author has the right to grant and does grant on behalf of all authors of the Work (as defined in the below author licence), an exclusive licence and/or a non-exclusive licence for contributions from authors who are: i) UK Crown employees; ii) where BMJ has agreed a CC-BY licence shall apply, and/or iii) in accordance with the terms applicable for US Federal Government officers or employees acting as part of their official duties; on a worldwide, perpetual, irrevocable, royalty-free basis to BMJ Publishing Group Ltd ("BMJ") its licensees and where the relevant Journal is co-owned by BMJ to the co-owners of the Journal, to publish the Work in this journal and any other BMJ products and to exploit all rights, as set out in our licence.

The Submitting Author accepts and understands that any supply made under these terms is made by BMJ to the Submitting Author unless you are acting as an employee on behalf of your employer or a postgraduate student of an affiliated institution which is paying any applicable article publishing charge ("APC") for Open Access articles. Where the Submitting Author wishes to make the Work available on an Open Access basis (and intends to pay the relevant APC), the terms of reuse of such Open Access shall be governed by a Creative Commons licence – details of these licences and which Creative Commons licence will apply to this Work are set out in our licence referred to above.

Other than as permitted in any relevant BMJ Author's Self Archiving Policies, I confirm this Work has not been accepted for publication elsewhere, is not being considered for publication elsewhere and does not duplicate material already published. I confirm all authors consent to publication of this Work and authorise the granting of this licence.

For peer review only

Indirect effects of the early phase of the COVID-19 pandemic on the coverage of essential maternal and newborn health services in a rural sub-district in Bangladesh: results from a cross-sectional household survey

Shema Mhajabin[1], Aniqa Tasneem Hossain[1], Nowrin Nusrat[1], Sabrina Jabeen[1], Shafiqul Ameen[1], Goutom Banik[1], Tazeen Tahsina[1], Anisuddin Ahmed[1], Qazi Sadeq-ur Rahman[1], Emily S. Gurley[3], Sanwarul Bari[1], Md. Atique Iqbal Chowdhury[1], Shams El Arifeen[1≈], Rajesh Mehta[≈4], Ahmed Ehsanur Rahman[1&2≈*]

[≈] Joint co-senior author

[1]- International Centre for Diarrhoeal Disease Research, Bangladesh (icddr,b)

[2]- University of Edinburgh

[3]- Johns Hopkins University

[4]- World Health Organization

*Corresponding Author: Dr Ahmed Ehsanur Rahman, icddr,b, Mohakhali, Dhaka-1212, Bangladesh, email: ehsanur@icddrb.org, Mobile Number: +8801727010050

Keywords: COVID-19 pandemic, Lockdown, rural area, essential services, maternal health, newborn health

Total word count, excluding title page, abstract, references, figures, and tables: 4300

For peer review only

ABSTRACT

Objective: This paper presents the effect of the early phase of COVID-19 on the coverage of essential maternal and newborn health (MNH) services in a rural sub-district of Bangladesh.

Design: Cross-sectional household survey with random sampling

Setting: Baliakandi sub-district, Rajbari district, Bangladesh

Participants: Data were collected from women who were third-trimster pregnant during early phase of the pandemic (111) and pre-pandemic period (115) to measure antenatal care (ANC) service coverage. To measure birth, postnatal care (PNC), and essential newborn service coverage (ENC), data were collected from women who had a history of delivery during early phase of pandemic (163) and pre-pandemic period (166).

Exposure: Early phase of pandemic included a strict national lockdown between April-June 2020, and pre-pandemic was defined as August-October 2019.

Outcome of interest: Changes in the coverage of selected MNH services (ANC, birth, PNC, ENC) during the early phase of COVID-19 pandemic comparing to the pre pandemic period, estimated by two-sample proportion tests

Findings: Among women who were third trimester pregnant during the early phase of pandemic period, 77% (95% CI: 70-85) received at least one ANC from a medically trained provider (MTP) during the third trimester, compared to 83% (95% CI: 76-90) during the pre-pandemic period. Among women who gave birth during the early phase of pandemic period, 72% (95% CI: 66-79) were attended by an MTP, compared to 63% (95% CI: 56-71) during the pre-pandemic period. Early initiation of breastfeeding was practiced among 38% (95% CI: 31-46) of the babies born during the early phase of the pandemic period. It was 37% (95% CI: 29-44) during the pre-pandemic period. After assessing the service coverage of the selected MNH services during the early phase of pandemic and pre-pandemic, no significant differences were

For peer review only - http://bmjopen.bmj.com/site/about/guidelines.xhtml

observed (i.e. p-value for at least one ANC from MTP equals to 0.33, p-value for birth attended by a MTP equals to 0.08, and p-value for early initiation of breastfeeding equals to 0.81). Only the coverage of at least one ANC from MTP significantly differed among the women who were seven months pregnant during early phase of pandemic and pre-pandemic (p-value<0.05).

Conclusion: The early phase of the pandemic which includes lockdown did not affect the selected MNH service coverage in the study area as people accessed their required services from available private health sectors.

Strengths and limitations of this study

- This paper provides a population level service coverage during the early phase of pandemic which national level routine information system cannot capture.
- Additionally, this paper compares the service coverage of the essential MNH services before and during the pandemic to understand the effect of pandemic in rural area where usually service coverage is less compared to urban area.
- This study acknowledges the limitation of the cross-sectional design in inferring any causality and estimating the impact.
- This study adapted the questionnaire based on the standard BDHS, BMMS, and MICS tools which are validated and globally accepted.
- This study considered only 6 months of recall for assessing the coverage during the early phase of pandemic period.

For peer review only - http://bmjopen.bmj.com/site/about/guidelines.xhtml

INTRODUCTION

The coronavirus disease 2019 (COVID-19), caused by the severe acute respiratory syndrome coronavirus 2 (SARS-CoV-2), has caused more than two hundred and forty-two million confirmed cases across 219 countries and territories and taken around five million lives as of October 2021, making it the deadliest pandemic since the Spanish flu in 1918 [1 2]. However, this is only the tip of the iceberg. Any pandemic negatively impacts the social, economic, and political landscape globally, regionally, and nationally [3-6]. The unexpected health burden compels the policymakers to shift focus from routine healthcare and repurpose the existing resources to tackle the more ominous threats posed by the pandemic [7-9]. It is, therefore, likely that the morbidity and mortality due to other causes may arise during a pandemic, especially in countries with limited resources [10 11].

The risks of COVID-19 infection and its adverse clinical consequences are relatively low among children and the younger population [12 13]. A multi-country cohort study found that pregnant individuals with COVID-19 diagnosis had higher rates of adverse outcomes, including maternal mortality, preeclampsia, and preterm birth compared with pregnant individuals without COVID-19 diagnosis [14]. Mothers and newborns are also vulnerable to the indirect effects of the pandemic [15-18]. Focusing on the emergency response may result in the de-prioritization of essential health services, including maternal and newborn health (MNH). The availability of essential MNH services may become severely compromised due to the repurposing of limited resources, overwhelming demand of other emergency services, healthcare workers' absence and deaths and reduced service hours or closure of health facilities [15 19-21]. Similarly, the access to and utilization of essential MNH services may be adversely affected by the hesitancy to seek care from health facilities due to the fear of contracting the virus and lack of reliable transportation due to the restrictive movement measures such as the lockdown [22 23]. A systematic review and meta-analysis of 40 studies reported that the global

For peer review only - http://bmjopen.bmj.com/site/about/guidelines.xhtml

burden of maternal deaths, stillbirth and maternal depression have increased during the COVID-19 pandemic, with a high disparity between the high-income and low- and middle-income country settings [24]. Another study reported that the under-5 deaths increased by 45% per month and the maternal deaths have increased by 36% per month, across 118 countries due to the indirect effect the COVID-19 pandemic using the survey data [15]. According to United Nations report, COVID-19 may have contributed to an estimated 11,000 additional maternal deaths and 228,000 additional child deaths in South Asia in 2020 using routine health information data such as District health information system (DHIS2) [16]. Such adverse indirect effects of the COVID-19 pandemic may abrade the major gains in improving MNH achieved over the past few decades.

On March 08, 2020, Bangladesh declared the first confirmed case of COVID-19 and the first death was reported on 18 March 2020. Since then, Bangladesh has reported more than seven hundred and seventy thousand confirmed cases, including more than 11,000 deaths [25]. The Government of Bangladesh declared "general leave" for a week on 26 March 2020 for interrupting the viral transmission chain and flattening the epidemic curve comparable to lockdown [26]. Later the "general leave" was extended for two months. In the beginning, all business except medical services were closed, and all kind of movement except medical emergencies were restricted. The lockdown measures were attenuated in the second month of lockdown. A sharp decline was observed in the utilization of essential MNH services during the lockdown period (March-May, 2020) in Bangladesh using the DHIS2 [27 28]. For example, the study reported that there was 41%, 52% and 56% decrease in April 2020 comparing to January 2020 for institutional normal deliveries in Upazilla Health Complex (UHCs), District Hospital (DHs), and tertiary hospitals. However, this was based on the DHIS2, routine health information system, which collects utilization data from public health facilities and some selected private facilities. Therefore, this might not reflect the change in the coverage of

For peer review only - http://bmjopen.bmj.com/site/about/guidelines.xhtml

essential MNH services estimated with a true population-level denominator. This paper presents the effect of the early phase of the pandemic on the coverage of essential MNH services in Baliakandi, a rural sub-district in Bangladesh. This paper will further observe the differences in selected MNH indicators by month of early phase of pandemic.

METHODS

Study design

We conducted a cross-sectional household survey to estimate the coverage of essential MNH services during the early phase of pandemic and pre-pandemic periods. Antenatal care (ANC) from a medically trained provider (MTP), birth attended by a MTP, Essential Newborn Care (ENC), and Postnatal Care (PNC) from a MTP within 72 hours of birth were identified as tracers for the essential MNH services. As the local administration had implemented pandemic measures the April-June 2020 period was operationally defined as the early phase of pandemic period. For identifying the pre-pandemic period (routine circumstances) we considered the following factors. China reported the first COVID-19 case in December 2019, and several global and national measures were taken between January and March 2020 and there may have been an impact on normal life and routine services. Before this, November 2019 – January 2020 period is the winter season in Bangladesh, and various cultural programs and family events are organized in this season, possibly leading to changes in the service use pattern. Hence, we considered August-October 2019 as the pre-pandemic period for comparison purposes.

Study setting

We conducted the study in a rural sub-district named Baliakandi, under the Rajbari district in Bangladesh. Since September 2017, International Center of Diarrhoeal Disease Research, Bangladesh (icddr,b) has been conducting health and demographic surveillance in this area [29]. The surveillance covers 261 villages and a population of about 227,540 (in 2019). Through two

For peer review only - http://bmjopen.bmj.com/site/about/guidelines.xhtml

monthly household visits, the surveillance data collectors collect information on births, deaths, marriages, divorces, and migrations. In addition to these vital events, they also collect information on pregnancy and birth outcomes.

Study participants and Outcome of interest

For assessing the effect of early phase of pandemic on the coverage of ANC, we randomly selected 126 women who were third trimester pregnant during April-June 2020 and gave birth in July 2020 and another 126 women who were third trimester pregnant during August-October 2019 and gave birth in November 2019 as study participants (Supplementary Figure 1). For estimating the coverage of birth attended by a MTP, ENC and PNC, we randomly selected 178 women who gave birth in April-June 2020 (as the effect of pandemic) and 178 women who gave birth in August-October 2019 (as the effect of the pre-pandemic).

We calculated the sample size to detect a minimum effect size of 33 percentage point (relative) between the early phase of pandemic and the pre-pandemic periods at 80% power (two-sided) and 5% error probability. We used the coverage estimates (rural) reported in 2017-18 Bangladesh Demographic and Health Survey (BDHS) for the selected essential MNH services as proxies for the pre-pandemic status [30]. The coverage estimates (rural) for the selected MNH services for pandemic period was collected using DHIS2 data. The unadjusted sample size was adjusted for a non-response rate of 10% and the clustering effect with 1.10 design effect. Details regarding the sample size calculation for each of the essential MNH services is presented in (Supplementary table 1). We used the surveillance data to generate the sampling frame based on specific inclusion criteria. Then, we used simple random sampling to select the required number of participants from each category (Supplementary figure 1).

Data collection tools

For peer review only - http://bmjopen.bmj.com/site/about/guidelines.xhtml

We used an interviewer-administered structured questionnaire to interview the eligible respondents. The questions were primarily adopted from the BDHS, Bangladesh Maternal Mortality Survey (BMMS), and Multiple Indicator Cluster Survey (MICS) [30-32]. The questionnaire consisted of seven main sections: household information, socio-economic status, respondent's background, pregnancy, birth, and postnatal care, ENC, care-seeking during emergencies, telemedicine service, and COVID-19 related knowledge and experiences. We translated the questionnaire in Bangla and pre-tested it before finalization.

For

Training and data collection

We recruited fifteen data collectors and two field supervisors from the local communities who were familiar with the local context, culture, and dialect. The data collection team received 5-days of face-to-face training, which was followed by two days of field testing. The training was conducted by the study investigators and other master trainers with special expertise and experience in household surveys.

Data were collected between 7 November to 25 December 2020. The field supervisors revisited a random sample of 5% of households to ensure quality. In addition, the field supervisors conducted unscheduled visits to observe the interviews and monitor the quality of data collection. Weekly meetings were organized to solve any emerging issues related to data collection. All questionnaires were manually reviewed by the field supervisors and the field research managers before sending for data entry.

We maintained social distancing and followed the national infection prevention and control guidelines during training and data collection [33]. All interviews were conducted outdoors. The data collection team received appropriate personal protective equipment such as masks, gloves, hand sanitizers, aprons, and face shields. Also, we distributed re-usable facemasks to the respondents to wear during the interviews.

For peer review only - http://bmjopen.bmj.com/site/about/guidelines.xhtml

Data analysis plan

We used the statistical software package STATA version 14.2 (StataCorp, College Station, TX) for data analysis [34]. At first, descriptive statistics were used to report the background characteristics of the respondents. We used the principal component analysis to generate an asset score for each household [35]. The asset scores were used to rank the households into quintiles. We reported the coverage of selected essential MNH services for the early phase of the pandemic and pre-pandemic periods separately with 95% confidence intervals (CI). Additionally, the service coverage of selected MNH indicators were presented by months of early phase of pandemic and pre-pandemic with 95% CI. The operational definitions of each of the indicators are presented in supplementary table 2, and the definition of providers are presented in supplementary table 3. Two sample proportion tests were conducted to explore the difference in the coverage of selected essential MNH services between the early phase of the pandemic and pre-pandemic periods. We reported any significant difference at $p<0.05$.

Ethical approval and consent to participate

We obtained the ethical approval to conduct the study from the Institutional Review Board of icddr,b (PR-20085). Written and informed consent was sought from each study participant before the interview. In the case of respondents who cannot read or write, we obtained audio-recorded verbal informed consent.

Patient and public involvement

There was no patient or public involvement in the study.

RESULTS

We interviewed 115 women (non-response rate 9%) who were third-trimester pregnant during the pre-pandemic period and 111 women (non-response rate 12%) who were third-trimester pregnant during the early phase of pandemic period. We also interviewed 166 women (non-

For peer review only - http://bmjopen.bmj.com/site/about/guidelines.xhtml

response rate 7%) who gave birth during the pre-pandemic period and 163 women (non-response rate 9%) who gave birth during the early phase of pandemic period. Table-1 describes the background characteristics of each group of respondents. Approximately one-third of the women were aged 20-24 years. Around half of them had incomplete secondary education (6-9 years of schooling), and one-fourth had complete secondary or higher education (10 or more years of schooling). Most of the respondents were Muslim. Approximately 40% of the women were primigravidae (first time pregnant). Less than 10% of them were engaged in any income-generating activities. No notable difference was observed in characteristics across the groups of respondents.

Table 1: Background characteristics of the respondents; presented in column percentage

Background characteristics	Women who were third trimester pregnant		Women who had a history of birth outcome	
	Pre-pandemic	Early phase of pandemic	Pre-pandemic	Early phase of pandemic
	N=115	N=111	N=166	N=163
	%	%	%	%
**Age**				
14 and below	0	1	0	0
15-19	23	23	14	22
20-24	31	36	33	37
25-29	21	25	26	22
30-34	19	13	22	12
35+	6	2	5	7
**Education**				
No education	3	1	5	3
Primary incomplete[1]	9	10	9	11
Primary complete[2]	7	9	12	11
Secondary incomplete[3]	56	48	52	52
Secondary complete or higher[4]	25	32	22	23
**Religion**				
Muslim	83	88	89	91
Hinduism/ Buddhism/ Christianity	17	12	11	9
**Parity**				
Primiparous	41	41	32	44

For peer review only - http://bmjopen.bmj.com/site/about/guidelines.xhtml

Multiparous	59	59	68	56
**Occupation**				
Housewife	96	95	95	90
Engaged in any income-generating activity	4	5	5	10
**Wealth quintile**				
Lowest	22	14	15	15
Second	23	19	24	24
Middle	25	23	25	23
Fourth	13	23	18	23
Highest	17	21	18	15
**Total**	100	100	100	100

[1]: 1-4 years of schooling
[2]: 5 years of schooling
[3]: 6-9 years of schooling
[4]: 10 or more years of schooling

For peer review only

Figure -1 summarises the coverage of essential MNH services during pre-pandemic and early phase of pandemic periods. Among women who were third-trimester pregnant during the early phase of pandemic period, 77% (95% CI: 70-85) received at least one ANC from a MTP during the third trimester, which was 83% (95% CI: 76-90) during the pre-pandemic period. Similarly, among women who were third-trimester pregnant during the early phase of pandemic period, 81% (95% CI: 74-88) received at least one ANC from a health facility during the third trimester, which was 83% (95% CI: 76-90) during the pre-pandemic period. No notable difference was observed in coverage of ANC (p-value equals 0.33 for at least one ANC from a MTP and p-value equals to 0.77 for at least one ANC from a facility) between the pre-pandemic and early phase of pandemic periods.

Among women who gave birth during the pandemic period, 72% (95% CI: 66-79) were attended by a MTP. It was 63% (95% CI: 56-71) among those who gave birth during the pre-pandemic period. The coverage of facility-birth was 65% (95% CI: 58-72) during the pandemic period and 55% (95% CI: 47-62) during the pre-pandemic period. No notable difference was observed in coverage of births attended by a MTP and facility-births between the pre-pandemic

For peer review only - http://bmjopen.bmj.com/site/about/guidelines.xhtml

and pandemic periods. The coverage of at least one PNC from a MTP was 58% (95% CI: 51-60) during the pandemic period and 52% (95% CI: 45-60) during the pre-pandemic period.

Among babies born during the pandemic period, the coverage of clean cord cutting practice was 65% (95% CI: 58-72). It was 59% (95% CI: 52-67) among those who were born during the pre-pandemic period. 7.1% chlorhexidine was applied to 16% (95% CI: 10-22) of babies born during the pandemic period, which was 18% (95% CI: 12-24) during the pre-pandemic period. Early initiation of breastfeeding was practised among 38% (95% CI: 31-46) of the babies born during the pandemic period. It was 37% (95% CI: 29-44) during the pre-pandemic period. No notable difference was observed in the coverage of ENC practices during the pandemic and the pre-pandemic periods.

Figure 1: Coverage of essential MNH services during the pre-pandemic and the pandemic periods, presented in percentage with 95% confidence intervals

Figure -2 illustrates the coverage of ANC received in the third trimester of pregnancy by months. Among the women who were third trimester pregnant during the pandemic period, 35% received at least one ANC from the MTP, 36% from a health facility and 32% from a private health facility during the 7th month of pregnancy (April 2020). These estimates were significantly lower than that of the pre-pandemic period among the women who were third trimester pregnant during the pre-pandemic period, 49% ($p<0.05$) received at least one ANC from the MTP (August 2019). No notable difference was observed in ANC coverage during the 8th and 9th month of pregnancy between the pandemic and pre-pandemic periods.

Figure 2: Coverage of ANC received in the third trimester during the pre-pandemic and the pandemic periods; presented in percentages by months of pregnancy

Among the women who were third-trimester pregnant during the pandemic period, 23% did not receive any ANC from a MTP and 43% received one ANC from a MTP during that period

For peer review only - http://bmjopen.bmj.com/site/about/guidelines.xhtml

(Supplementary figure 2). Among the women who were third-trimester pregnant during the pre-pandemic period, only 17% did not receive any ANC from a MTP and 40% received one ANC from a MTP during that period. We did not observe any notable difference (statistically significant at $p<0.05$) in the above-mentioned distribution of ANC status between the pandemic and the pre-pandemic periods. The care-seeking for ANC using telemedicine services were very low and not different between pandemic and pre-pandemic period (Supplementary figure 3).

Table 2 presents the type of health care provider from who the women received ANC during their third trimester of pregnancy. Among the women who were third-trimester pregnant during the pandemic period, 77% received ANC from a doctor whereas 80% received ANC from a doctor during the pre-pandemic period. No notable difference (statistically significant at $p<0.05$) was observed in the above-mentioned distribution of ANC providers between the pandemic and the pre-pandemic periods.

Table 2: Type of provider from who ANC was received in the third trimester during the pre-pandemic and early phase of pandemic periods; presented in percentages, multiple responses considered

Type of providers	Pre-pandemic(95% CI)	Early phase of pandemic (95% CI)
Doctor	80 (73-87)	77 (70-78)
Nurse/midwives/paramedic	20 (13-27)	11 (5-17)
FWV	11 (6-17)	7 (2-12)
CSBA	0	0
SACMO/MA/CHCP/HA/FWA	8 (3-13)	13 (6-19)
NGO Worker/ TBA	2 (0-4)	6 (2-11)

Figure-3 presents the coverage of birth attended by a MTP, birth in a health facility, birth in a private health facility, and birth through C-sections. Among women who gave birth during the pandemic period, the coverage of birth attended by a MTP was around 70% across all three months. Similarly, the coverage of facility-birth was more than 60% across all three months.

For peer review only - http://bmjopen.bmj.com/site/about/guidelines.xhtml

Around 50% of birth occurred in a private health facility, and the rates were consistent across the three months. We did not observe any notable difference in the coverage attended by a MTP, birth in a health facility, birth in a private health facility and birth through C-sections during the pandemic and pre-pandemic periods for any of the reporting months.

Figure 3: Coverage of births attended by a medically trained provider, birth in a health facility, birth in a private health facility and birth through C-sections during the pre-pandemic and pandemic periods; presented in percentages by calendar months

Table 3 presents the type of health care providers attending the births which were conducted during the pre-pandemic and the pandemic period. Among the women who gave birth during the pandemic period, 53% were attended by a doctor and 71% by a nurse/midwife. Among the women who gave birth during the pre-pandemic period, 49% were attended by a doctor and 62% from a nurse/midwife. No notable difference (statistically significant at $p<0.05$) was observed in the distribution of providers attending births between the pandemic and the pre-pandemic periods.

Table 3: Type of provider attending the births during the pre-pandemic and early phase of pandemic periods; presented in percentages

Type of providers	Pre-pandemic (95% CI)	Early phase of pandemic (95% CI)
Doctor	49 (41-56)	53 (46-61)
Nurse/midwives/paramedic	62 (55-69)	71 (64-78)
FWV	0	1 (0-2)
CSBA	2 (-1,4)	0
SACMO/MA/CHCP/HA/FWA	4 (1-6))	2 (0-4)
NGO Worker/ TBA	40 (33-48)	35 (28-41)

Figure-4 shows the coverage of PNC from a MTP, PNC from a health facility, and PNC from a private health facility during the pre-pandemic and the pandemic periods. Among women who gave birth during the pandemic period, the coverage of PNC by a MTP within 72 hours of

For peer review only - http://bmjopen.bmj.com/site/about/guidelines.xhtml

birth was around 60% across all three months. Similarly, the coverage of PNC in a health facility facility-birth was around 55% across all three months. We did not observe any notable difference in the coverage of PNC by a MTP, PNC in a health facility, and PNC in a private health facility during the pandemic and pre-pandemic periods for any of the reporting months.

Figure 4: Coverage of PNC by a medically trained provider, PNC in a health facility, PNC in a private health facility among births occurred during the pre-pandemic and pandemic periods; presented in percentages by calendar months

Figure-5 presents the coverage of different component of ENC among the newborns during the pre-pandemic and the pandemic periods. Among babies born at the early phase of pandemic (April 2020), the coverage of early initiation of breastfeeding was 24%, which was much lower than that of the remaining months (May-June 2020) during the pandemic period and the pre-pandemic period. No notable difference was observed in the coverage of other components of ENC between the pandemic and the pre-pandemic periods by calendar months.

Figure 5: Coverage of different components of ENC during the pre-pandemic and pandemic periods; presented in percentages by calendar months

DISCUSSION

The health care-seeking experience for ANC, birth, PNC, and ENC during early phase of COVID-19 pandemic in rural Bangladesh was not found affected in this study. We found that the coverage of selective essential MNH services during the early phase of pandemic was not different from that of the pre-pandemic period in this rural sub-district in Bangladesh. However, national level data from the routine health system of Bangladesh (DHIS2) showed a substantial decline in the utilization of essential MNH services [27]. The possible explanations for such contradicting findings are summarized under two broad themes, i.e., how the service utilization was restored (health system response and community coping strategy and resilience)

For peer review only - http://bmjopen.bmj.com/site/about/guidelines.xhtml

and why we did not see any difference in the service utilization (private sector coverage, enforcement of lockdown measures, and measurement issues in the routine health information system) are discussed below.

Health systems responses:

Several health systems initiatives taken by the Government of Bangladesh might have contributed to overcoming the initial shock of the pandemic and prevent a substantial decline in the coverage of key MNH services. In March 2020, the Government developed the Bangladesh Preparedness and Response Plan for COVID-19, which also emphasized the importance of maintaining essential health and nutrition services during the pandemic [36]. After the initial focus on COVID-19-specific preparation and responses, several initiatives were taken to promote the availability and readiness of essential MNH services. In May 2020, the Ministry of Health, with technical inputs from the development partners and professional societies, developed the "National Guideline for providing essential Maternal, Newborn and Child Health Services in the context of COVID-19". The prime objective of this guideline was to inform the health care providers and facility- and district-level managers of the importance of providing essential MNCH services in the context of COVID-19 [37]. It also provided specific guidance on continuing the essential services safely by maintaining physical distancing, using personal protective equipment, and promoting infection prevention and control practices. The Ministry of Health organized a series of online sessions to sensitize the facility- and district-level managers. Afterwards, face-to-face training was organized for the frontline health care workers responsible for providing MNH services. Then national-level policymakers organized virtual meetings with facility-, district- and regional-level managers to review the MNH utilization data in respective health facilities and discuss strategies for overcoming the bottlenecks, barriers, and operational challenges. Special initiatives were also taken for establishing functional triage, isolation unit for suspected COVID-19 patients, and red zone for

For peer review only - http://bmjopen.bmj.com/site/about/guidelines.xhtml

confirmed COVID-19 patients at referral facilities, which promoted a safer environment at the facility for receiving routine services and might have contributed to restoring the public confidence for availing essential MNH services. In June 2020, the Government of Bangladesh recruited and deployed 2000 doctors and 5000 nurses for COVID-19 response [38]. The influx of additional human resources helped re-deploying the MNH services to their original duties and minimize the impact of COVID-19 on essential services. These national efforts might have positively influenced minimizing the disruption in the availability and provision of essential services in our study settings. According to the national routine health information system, there was little difference in the utilization of essential MNH services from public health facilities in the Baliakandi sub-district during the early phase of pandemic period [27]. This is particularly applicable for the Upazila Health Complex, which is the primary referral point for all health centres in the study site. We also did not observe any notable difference in the utilization of MNH services from the district hospital during that period, which is the primary referral point for all the Upazila Health Complexes in that district [27]. It indicates that most of the public health referral facilities continued providing routine services despite prioritizing the COVID-19 specific services. Since the referral facilities have more human resources than health centres, which have only one to two dedicated providers, it was easier for them to adopt an alternate approach for continuing essential MNH services.

For peer review only

Community coping strategy and resilience:

Bangladesh is one of the most vulnerable countries to climate variability and fluctuation [44 45]. A quarter of the land in Bangladesh is barely above sea level and is susceptible to the effects of sea water rise and increased salinity due to climate change. Bangladesh also experiences other forms of natural disasters like flood and cyclones quite frequently [46]. The people in Bangladesh have learnt to cope with the emergencies and shows resilience by focusing on rebuilding after the initial shock, mostly through local-level initiatives [47 48]. Awareness

For peer review only - http://bmjopen.bmj.com/site/about/guidelines.xhtml

regarding the importance of receiving MNH services from appropriate places and providers has increased substantially and consistently over the past couple of decades, even in the rural context. Hence the awareness and the resilience nature of local communities might have contributed to finding ingenious ways and coping strategies to avail the essential MNH service during the pandemic overcoming the challenges imposed by the lockdown.

Private sector coverage:

Bangladesh has a pluralistic health system constituted by the public sector, for-profit formal private sector and informal care providers [39 40]. Although the Government of Bangladesh has a large network of health infrastructure throughout the country, the majority of MNH services are provided by the poorly regulated for-profit private sector and unregulated informal care providers. According to 2017-18 BDHS, more than two-thirds of the ANC and facility-births took place in private health facilities [30]. In the past decade, the coverage of births in private health facilities has increased by four folds [30 41]. A similar trend was also observed for other essential MNH services such as ANC, PNC, and ENC. Although almost all the public health facilities report to DHIS2, very few private hospitals and private practitioners report to DHIS2. This disconnects between the larger market share of the private sector and disproportionately minor contribution in the DHIS2-based reporting system may explain our study findings. We did not observe any notable change in the coverage of essential MNH services during the early phase of pandemic period. In contrast, the DHIS2-based reporting system was indicating a substantial decline in service utilization nationally. In our study site, around 55% of births took place in the health facilities during the pre-pandemic period, of which about 80% were in private health facilities. The condition did not change during the pandemic period as 65% of births took place in health facilities, of which more than 80% were in private health facilities. We observed similar patterns for ANC and PNC services. In the rural sub-district where we conducted the study, there are three for-profit private health facilities that provide MNH

For peer review only - http://bmjopen.bmj.com/site/about/guidelines.xhtml

services [29]. In addition, there are 16 for-profit private facilities and two non-profit NGO clinics in the district headquarter, and many of them offer MNH services. Therefore, the prioritization of emergency services related to COVID-19 exclusively through the public health facilities may explain the initial decline in utilization of essential MNH services in those facilities, but the overwhelming majority in the market share the private sector and the continuation of the routine services may have contributed to sustaining the coverage essential MNH services in the early stage of the pandemic.

Enforcement of lockdown measures:

The Government of Bangladesh imposed a nationwide restriction of movements in late March 2020 to interrupt the pathways of COVID-19 transmission [26 42 43]. However, the Government declared it as 'general leave' instead of lockdown, which undermined the importance of adhering to the recommended measures among the mass population. At the beginning of the lockdown, all the government and non-government offices, factories and academic institutes were asked to shut down. A large proportion of city dwellers travelled back to their village homes, maintaining very little social distancing and other infection prevention and control measures [42]. After a couple of weeks, some government offices, and factories, including all readymade garment factories, were allowed to resume their operations, forcing many people to return to their workplace. Hence, the restrictions regarding inter-district and within-district movements gradually weaned off, and the public transportation options slowly resumed back with some infection prevention and control regulations [26]. Moreover, the lockdown measures were less coercively imposed in rural settings as the transportation services were reasonably available, allowing people to move within the sub-district and to the district headquarter when required. In our study, less than 5% of the respondents mentioned lack of transportation as a barrier to care-seeking, and less than 10% reported spending extra money for the vehicle rent while receiving essential MNH services during the lockdown period. Based on a national

For peer review only - http://bmjopen.bmj.com/site/about/guidelines.xhtml

survey, around two-thirds of the pregnant women in rural settings can reach a private health facility or provider within five-kilometre of travel distance [30]. It implies that people could avail the essential MNH services from nearby private health facilities when the services were temporarily disrupted in public health facilities in the early stage of the pandemic.

Measurement issues:

As mentioned earlier, there are limitations in estimating the coverage of health services through the routine health information system in Bangladesh. The Government of Bangladesh has made a substantial investment in strengthening the routine health information system by introducing DHIS2 in 2009 [39]. All the public health facilities and few private health facilities report their health service use data to DHIS2. Although MNH is a priority in the DHIS2-based reporting system in Bangladesh, the system's reach is not enough to accurately capture population-level denominators such as live births, which are essential for measuring the coverage of MNH services. In 2019, DHIS2 reported a total of 931,723 live births in Bangladesh [27]. Based on the crude birth rate reported in 2017-18 BDHS, the size of the annual birth cohort is more than 3 million in Bangladesh [30]. Similarly, DHIS2 reported that around 655,542 women received 4 ANC from the reporting facilities in 2019 [27]. However, an estimated 1,410,000 (47% of 3 million births) women received four or more ANC based on the national survey estimate [30]. Moreover, there are issues with the accuracy and quality of reporting in the routine health information systems, including DHIS2, particularly in low- and middle-income country settings [49-52]. Due to these gaps in accurately capturing the denominators and numerators through DHIS2-based reporting system, the decline observed in the utilization of essential MNH services in public health facilities in the early stage of the pandemic may not reflect the true change in the coverage of relevant services.

Secondly, there were gaps and delays in the documentation and reporting of routine services in the initial phase of the pandemic, particularly during the lockdown period due to the reshaping

For peer review only - http://bmjopen.bmj.com/site/about/guidelines.xhtml

of the health care providers' responsibilities and health systems responses. The Government of Bangladesh prepared the National Preparedness and Response Plan for the pandemic and repurposed the existing infrastructure, human resource, and logistics to prevent and treat COVID-19 [36]. Initially, triage was introduced in all public health facilities and some hospitals were dedicated to treating COVID-19. Additionally, a COVID-19 dashboard has been developed and is updated daily [25]. This portal displays data on the number of laboratories testing for COVID-19 and the number of health facilities treating COVID-19 patient. The dashboard also displays information on the number of tests performed, number of cases identified, number of people in quarantine and isolation, and number of deaths daily. It also provides an overview of the stock availability of COVID-19 related logistics of all health facilities. The frontline health care workers, facility and district level managers and national policymakers prioritized capturing and reporting of COVID-19 related data to monitor the relevant implementation and response status. Hence, the introduction and emphasis on COVID-19 related services, documentation and reporting might have overwhelmed the health care providers and the health systems resulting in the initial gaps and delays of reporting other routine services, including MNH. For example, we accessed DHIS2 in June 2020 and observed a sharp decline in the utilization of essential MNH services from public health facilities in March-May 2020 [27]. We accessed DHIS2 in September 2020 and found that the service utilization numbers were updated for the same time March-May 2020, resulting in a much smaller decline than the pre-COVID-19 period. Therefore, the true impact on the coverage of essential MNH services might be less than what was originally observed based on DHIS2 utilization data in the early phase of the pandemic.

Conclusion

Our study findings reported no significant changes in the overall coverage of MNH services in rural area of Bangladesh. Moreover, the changes were not even significant for monthly

For peer review only - http://bmjopen.bmj.com/site/about/guidelines.xhtml

comparison during the early phase of pandemic and pre-pandemic periods. It highlights the importance of expanding and reinforcing the routine health information system to cover the information from the private health facilities and community services for comprehensive monitoring of essential services, particularly during the emergency response. The results also reveal the strength of local level coping strategy and coping strategy to overcome the immediate shock.

For peer review only

For peer review only - http://bmjopen.bmj.com/site/about/guidelines.xhtml

REFERENCE

1. Worldometers. COVID-19 CORONAVIRUS PANDEMIC 2021 [Available from: https://www.worldometers.info/coronavirus/ accessed April 28 2021.
2. (WHO) WHO. WHO Coronavirus (COVID-19) Dashboard 2021 [Available from: https://covid19.who.int/ accessed April 28 2021.
3. Davies SE. National security and pandemics. *UN Chronicle* 2013;50(2):20-24.
4. Drake TL, Chalabi Z, Coker R. Cost-effectiveness analysis of pandemic influenza preparedness: what's missing? *Bulletin of the World Health Organization* 2012;90:940-41.
5. Tisdell CA. Economic, social and political issues raised by the COVID-19 pandemic. *Economic analysis and policy* 2020;68:17-28.
6. Qiu W, Rutherford S, Mao A, et al. The pandemic and its impacts. *Health, culture and society* 2017;9:1-11.
7. Chowdhury A. Repurposing innovations: Learning from Bangladesh's rapid response to the pandemic', 2020.
8. Ahmed T, Rahman AE, Amole TG, et al. The effect of COVID-19 on maternal newborn and child health (MNCH) services in Bangladesh, Nigeria and South Africa: call for a contextualised pandemic response in LMICs. *International journal for equity in health* 2021;20(1):1-6.
9. Her M. Repurposing and reshaping of hospitals during the COVID-19 outbreak in South Korea. *One Health* 2020;10:100137.
10. Wilhelm JA, Helleringer S. Utilization of non-Ebola health care services during Ebola outbreaks: a systematic review and meta-analysis. *J Glob Health* 2019;9(1)
11. Jones SA, Gopalakrishnan S, Ameh CA, et al. 'Women and babies are dying but not of Ebola': the effect of the Ebola virus epidemic on the availability, uptake and outcomes of maternal and newborn health services in Sierra Leone. *BMJ global health* 2016;1(3):e000065.
12. Sinha IP, Harwood R, Semple MG, et al. COVID-19 infection in children. *The Lancet Respiratory Medicine* 2020
13. zazhi ZlxbxzzZl. The epidemiological characteristics of an outbreak of 2019 novel coronavirus diseases (COVID-19) in China. *Novel, Coronavirus Pneumonia Emergency Response Epidemiology* 2020;41(2):145.
14. Villar J, Ariff S, Gunier RB, et al. Maternal and Neonatal Morbidity and Mortality Among Pregnant Women With and Without COVID-19 Infection: The INTERCOVID Multinational Cohort Study. *JAMA Pediatrics* 2021;175(8):817-26. doi: 10.1001/jamapediatrics.2021.1050
15. Roberton T, Carter ED, Chou VB, et al. Early estimates of the indirect effects of the COVID-19 pandemic on maternal and child mortality in low-income and middle-income countries: a modelling study. *The Lancet Global Health* 2020;8(7):e901-e08.
16. UNICEF. Direct and indirect effects of the COVID-19 pandemic and response in South Asia: UNICEF, 2021.
17. Busch-Hallen J, Walters D, Rowe S, et al. Impact of COVID-19 on maternal and child health. *The Lancet Global Health* 2020;8(10):e1257.
18. Ilerioluwa Oke G, Ebuka Elebesunu E, Ihekweazu V. Impact of COVID-19 Pandemic on Maternal and Child Health. *Modern Care Journal* 2020;17(4)
19. Elston JW, Cartwright C, Ndumbi P, et al. The health impact of the 2014-15 Ebola outbreak. *Public health* 2017;143:60-70. doi: 10.1016/j.puhe.2016.10.020 [published Online First: 2017/02/06]
20. Semaan A, Audet C, Huysmans E, et al. Voices from the frontline: findings from a thematic analysis of a rapid online global survey of maternal and newborn health professionals facing the COVID-19 pandemic. *BMJ Glob Health* 2020;5(6) doi: 10.1136/bmjgh-2020-002967 [published Online First: 2020/06/27]
21. Hassanian-Moghaddam H, Zamani N, Kolahi A-A. COVID-19 pandemic, healthcare providers' contamination and death: an international view. *Critical Care* 2020;24(1):208. doi: 10.1186/s13054-020-02938-y

For peer review only - http://bmjopen.bmj.com/site/about/guidelines.xhtml

22. Pant S, Koirala S, Subedi M. Access to maternal health services during COVID-19. *Europasian Journal of Medical Sciences* 2020;2(2):48-52.
23. Temesgen K, Wakgari N, Debelo BT, et al. Maternal health care services utilization amidstCOVID-19 pandemic in West Shoa zone, central Ethiopia. *PloS one* 2021;16(3):e0249214.
24. Chmielewska B, Barratt I, Townsend R, et al. Effects of the COVID-19 pandemic on maternal and perinatal outcomes: a systematic review and meta-analysis. *The Lancet Global Health* 2021
25. Director Genereal of Health Services B. Coronavirus COVID-19 Dashboard, 2020 2020 [Available from: http://dashboard.dghs.gov.bd/webportal/pages/covid19.php accessed April 28 2021.
26. Shammi M, Bodrud-Doza M, Islam ARMT, et al. Strategic assessment of COVID-19 pandemic in Bangladesh: comparative lockdown scenario analysis, public perception, and management for sustainability. *Environment, Development and Sustainability* 2020:1-44.
27. Director Genereal of Health Services B. DHIS2 Central Database (Upazila level and Above) 2021 [Available from: https://dghs.gov.bd/index.php/en/component/content/article?id=456 accessed April 28 2021.
28. Wangmo S, Sarkar S, Islam T, et al. Maintaining essential health services during the pandemic in Bangladesh: the role of primary health care supported by routine health information system. *WHO South-East Asia Journal of Public Health* 2021;10(3):93.
29. CHAMPS. CHAMPS in Bangladesh 2021 [cited 2021. Available from: https://champshealth.org/sites-bangladesh/ accessed 01 June 2021.
30. National Institute of Population Research and Training (NIPORT), ICF International. Bangladesh Demographic and Health Survey 2017-18: Key indicators. Dhaka, Bangladesh, and Rockville, Maryland, USA, 2019.
31. National Institute of Population Research and Training (NIPORT), International Centre for Diarrhoeal Disease Research Bangladesh (icddr b, MEASURE Evaluation. Bangladesh Maternal Mortality and Health Care Survey 2016: Preliminary Report. Dhaka, Bangladesh and Chapel Hill, NC, USA, 2017.
32. Unicef. MULTIPLE INDICATOR CLUSTER SURVEY 2016: NBS, UNICEF, 2018.
33. Directorate General of Health Services (DGHS) MoHaFWM, Bangladesh. NATIONAL GUIDELINE ON INFECTION PREVENTION AND CONTROL IN HEALTHCARE SETTINGS WITH ADDITIONAL MEASURES FOR COVID-19: Directorate General of Health Services (DGHS), Ministry of Health and Family Welfare (MOHFW), Bangladesh, 2020.
34. StataCorp. STATA version 14.2 2021 [updated 2021; cited 2021. Available from: https://www.stata.com/ accessed 01 June, 2021 2021.
35. Wold S, Esbensen K, Geladi P. Principal component analysis. *Chemometrics and intelligent laboratory systems* 1987;2(1-3):37-52.
36. Ministry of Health and Family Welfare (MoH&FW). National Preparedness and Response Plan for COVID-19 Bangladesh: Directorate General of Health Services, Health Service Division, Ministry of Health and Family Welfare, Bangladesh, 2020.
37. MNCAH D. National Guideline for providing essential Maternal, Newborn and Child Health Services in the context of COVID-19. May 2020
38. bdnews24.com. Bangladesh moves to recruit 2,000 doctors, 5,000 nurses amid virus outbreak. May 2020
39. Khan MAH, Azad AK, de Oliveira Cruz V. Bangladesh's digital health journey: reflections on a decade of quiet revolution. *WHO South-East Asia journal of public health* 2019;8(2):71-76.
40. Organization WH. Bangladesh health system review: Manila: WHO Regional Office for the Western Pacific 2015.
41. National Institute of Population Research and Training (NIPORT), Mitra and associates, Macro International. Bangladesh Demographic and Health Survey 2007. Dhaka, Bangladesh, and Rockville, Maryland, USA, 2007.
42. Anwar S, Nasrullah M, Hosen MJ. COVID-19 and Bangladesh: Challenges and how to address them. *Frontiers in public health* 2020;8

For peer review only - http://bmjopen.bmj.com/site/about/guidelines.xhtml

43. Shammi M, Bodrud-Doza M, Islam ARMT, et al. COVID-19 pandemic, socioeconomic crisis and human stress in resource-limited settings: A case from Bangladesh. *Heliyon* 2020;6(5):e04063.
44. Naser MM, Swapan MSH, Ahsan R, et al. Climate change, migration and human rights in Bangladesh: Perspectives on governance. *Asia Pacific Viewpoint* 2019;60(2):175-90.
45. Shahid S. Probable impacts of climate change on public health in Bangladesh. *Asia Pacific Journal of Public Health* 2010;22(3):310-19.
46. Rahman R, Salehin M. Flood risks and reduction approaches in Bangladesh. Disaster risk reduction approaches in Bangladesh: Springer 2013:65-90.
47. Parvin GA, Shimi AC, Shaw R, et al. Flood in a changing climate: The impact on livelihood and how the rural poor cope in Bangladesh. *Climate* 2016;4(4):60.
48. Younus MAF, Kabir MA. Climate change vulnerability assessment and adaptation of Bangladesh: Mechanisms, notions and solutions. *Sustainability* 2018;10(11):4286.
49. Day LT, Rahman QS-u, Rahman AE, et al. Assessment of the validity of the measurement of newborn and maternal health-care coverage in hospitals (EN-BIRTH): an observational study. *The Lancet Global Health* 2021;9(3):e267-e79.
50. Tahsina T, Hossain AT, Ruysen H, et al. Immediate newborn care and breastfeeding: EN-BIRTH multi-country validation study. *BMC pregnancy and childbirth* 2021;21(1):1-17.
51. Day LT, Gore-Langton GR, Rahman AE, et al. Labour and delivery ward register data availability, quality, and utility-Every Newborn-birth indicators research tracking in hospitals (EN-BIRTH) study baseline analysis in three countries. *BMC health services research* 2020;20(1):1-14.
52. Bhattacharya AA, Umar N, Audu A, et al. Quality of routine facility data for monitoring priority maternal and newborn indicators in DHIS2: A case study from Gombe State, Nigeria. *PloS one* 2019;14(1):e0211265.

For peer review only

For peer review only - http://bmjopen.bmj.com/site/about/guidelines.xhtml

AUTHORS' CONTRIBUTION

SM and AER are the co-principal investigator of this study, and they conceptualised the study. SM took the lead of developing the first draft of the manuscript. SJ, SA, GB, TT, AA, ESG, SB, and MAIC provided their critical feedbacks on each draft of the manuscript. SM, ATH, NN, QSR, and AER have conducted the data analysis and interpretated the findings. All the authors have contributed significantly to design the study conducted and to prepare the final draft of the manuscript. All authors have read and approved the submission. AER, SEA, and RM have led the process as the senior authors.

Data availability statement

The data will be available upon request as per the organizations' policy.

FUNDING STATEMENT

The study was supported by the World Health Organization (WHO) under the grant of BGT-0031/2020.

COMPETING INTERESTS STATEMENT

There is no competing or conflicting interest.

ACKNOWLEDGEMENT

The authors would like to acknowledge the kind support of the Baliakandi demographic surveillance site which is supported by the CHAMPS project by Gates Foundation by Emory University throughout the data collection. icddr,b is also grateful to the Governments of Bangladesh, Canada, Sweden, and the UK for providing unrestricted/institutional support.

For peer review only - http://bmjopen.bmj.com/site/about/guidelines.xhtml

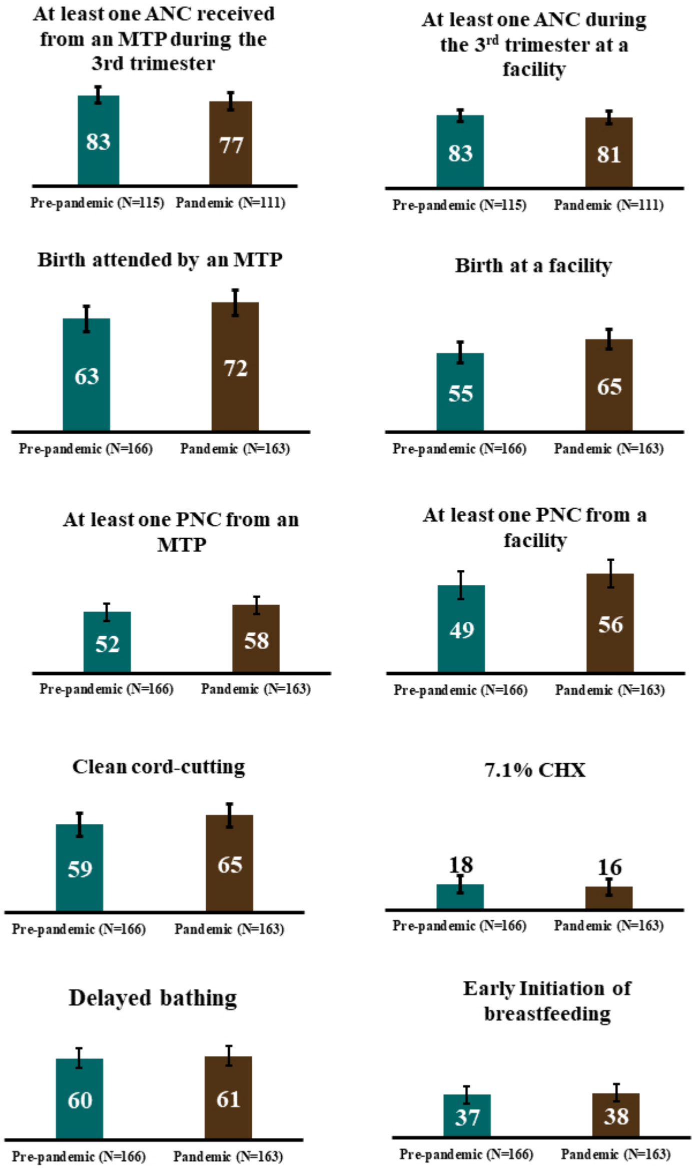

Coverage of essential MNH services during the pre-pandemic and the pandemic periods, presented in percentage with 95% confidence intervals

116x168mm (157 x 157 DPI)

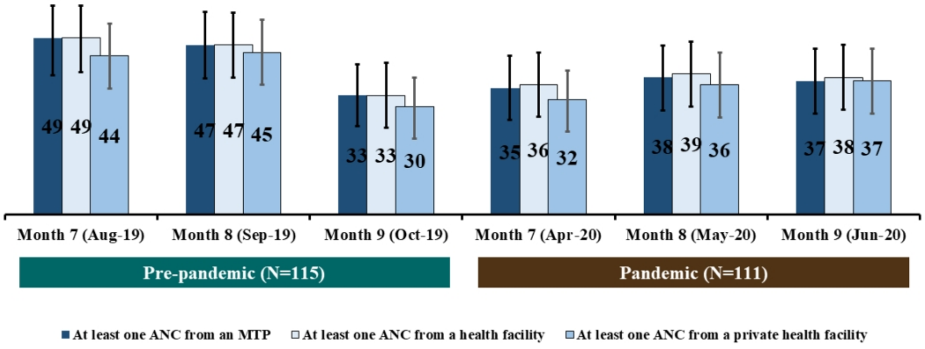

Coverage of ANC received in the third trimester during the pre-pandemic and the pandemic periods; presented in percentages by months of pregnancy

207x116mm (157 x 157 DPI)

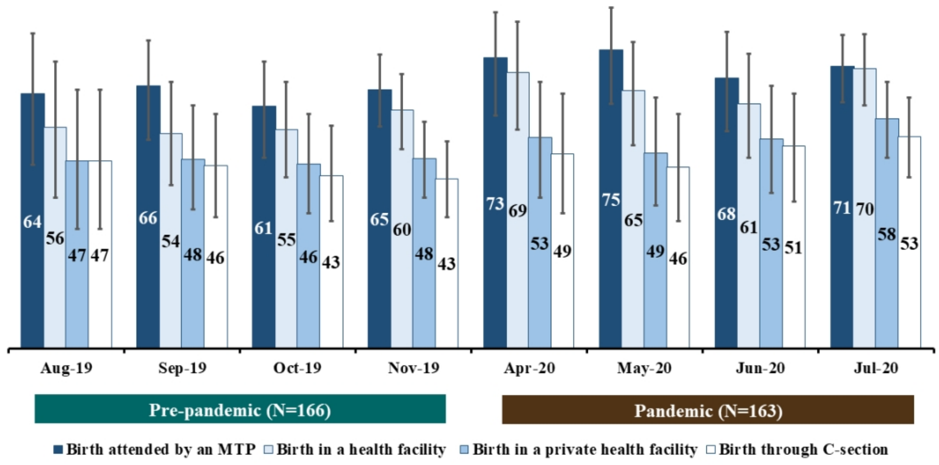

Coverage of births attended by a medically trained provider, birth in a health facility, birth in a private health facility and birth through C-sections during the pre-pandemic and pandemic periods; presented in percentages by calendar months

207x116mm (157 x 157 DPI)

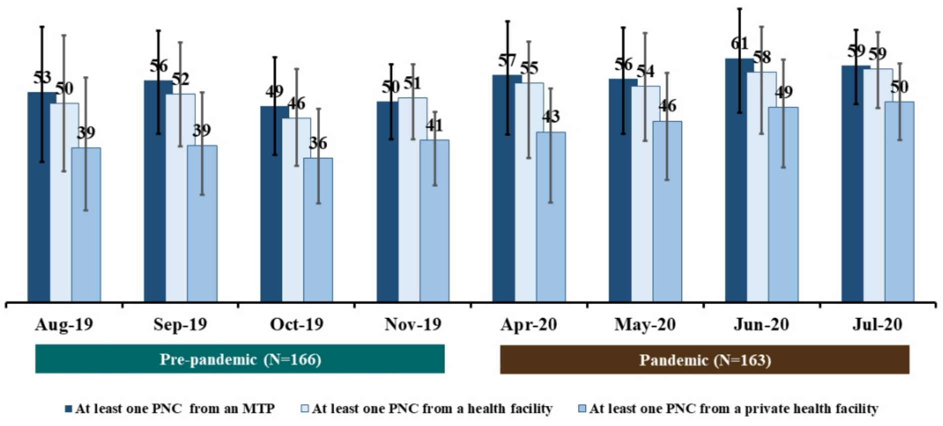

Coverage of PNC by a medically trained provider, PNC in a health facility, PNC in a private health facility among births occurred during the pre-pandemic and pandemic periods; presented in percentages by calendar months

207x116mm (157 x 157 DPI)

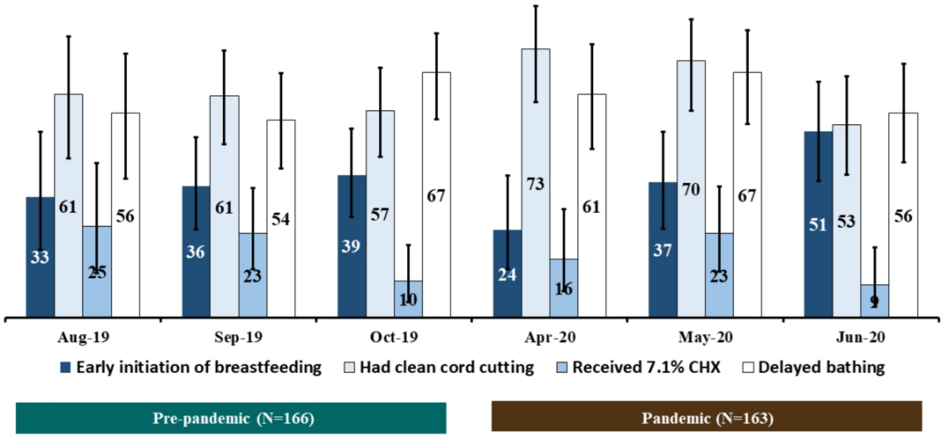

Coverage of different components of ENC during the pre-pandemic and pandemic periods; presented in percentages by calendar months

207x116mm (157 x 157 DPI)

Supplementary Table 1: Summary of indicator selected for each target population with required sample size

**Target population**	**Indicator**	**Baseline (pre-lockdown period)**	**Endline (Lockdown period)**	**Required Sample Size**
Women who were third trimester pregnant during the COVID-19' lockdown' period and the selected pre-lockdown period in the preceding year	At least one ANC from MTP<br>At least one ANC from facility	60%	30%	126
Women who had a history of birth during the COVID-19' lockdown' period and the selected pre-lockdown period in the preceding year	Birth attended by an MTP<br>Birth in a health facility<br>At least one PNC from an MTP within 48 hours of birth<br>Newborns received essential newborn care (ENC)	50%	33%	178

For peer review only

Supplementary Table 2: Definition of selected indicator

Selected indicators	Definitions
Indicator 1	Proportion of women who were third trimester pregnant during the COVID-19' lockdown' period and the pre-lockdown period in the preceding year receiving at least one ANC from MTP
Indicator 2	Proportion of women who were third trimester pregnant during the COVID-19' lockdown' period and the pre-lockdown period in the preceding year receiving at least one ANC from facility
Indicator 3	Proportion of women giving birth during COVID-19' lockdown' period and the corresponding period in the preceding year were attended by an MTP
Indicator 4	Proportion of women giving birth during COVID-19' lockdown' period and the corresponding period in the preceding year at a facility
Indicator 5	Proportion of newborn giving birth during the COVID-19' lockdown' period and the pre-lockdown period in the preceding year receiving PNC from an MTP within 48 hours of birth
Indicator 6	Proportion of newborns having born during the COVID-19' lockdown' period and the pre-lockdown period in the preceding year received drying
Indicator 7	Proportion of newborns having born during the COVID-19' lockdown' period and the pre-lockdown period in the preceding year had clean cord cutting
Indicator 8	Proportion of newborns having born during the COVID-19' lockdown' period and the pre-lockdown period in the preceding year received 7.1% chlorohexidine
Indicator 9	Proportion of newborns having born during the COVID-19' lockdown' period and the pre-lockdown period in the preceding year had delayed bathing
Indicator 10	Proportion of newborns having born during the COVID-19' lockdown' period and the pre-lockdown period in the preceding year had early initiation of breastfeeding

For peer review only

Supplementary Table 3: Definition of providers and facilities

Type of providers		Definition
Medically trained provider (MTP)	Doctors (General physicians)	This provider has five years of training on medicine, surgery, gynaecology, and obstetrics with one-year internship in medicine, surgery, gynaecology, and obstetrics wards. This provider is qualified to provide ANC, Basic emergency obstetric services (BEOC), PNC, menstrual regulation (MR), abortion care (AC), family planning services (FPS).
	Nurse	This provider completes 3-4 years of nursing course with 6 months dedicated to obstetrician training. This provider is qualified to provide ANC, BEOC, PNC, MR, AC, FPS.
	Midwife	This provider completes one year of midwifery training with 6 months dedicated to obstetrician training. This provider is qualified to provide ANC, BEOC, PNC, MR, AC, FPS.
	Family welfare visitor (FWV)	This provider completes 18 months of training on maternal and child health, family planning, and contraception. This provider is qualified to provide facility-based ANC, BEOC, PNC, MR, AC, FPS.
	Community Skilled Birth Attendant (CSBA)	This provider completes 6 months of training on BEOC and ENC. This provider is qualified to provide community-based ANC, BEOC, PNC, FPS.
Non-medically trained provider (non-MTP)	Family Welfare Assistant (FWA)	This provider completes 30 days of training on family planning. This provider is qualified to provide community-based FPS.
	Health assistant (HA)	This provider completes 3 months of training on limited preventive and curative care, immunization. This provider is qualified to provide community-based immunization of mother and children.
	Sub Assistant Community Medical Officer (SACMO)	This provider does not have any obstetrician training.
	Community Health Care Provider (CHCP)	This provider does not have any obstetrician training.
	Medical assistants (MA)	This provider completes 3 years of training on treatment of common disorders.

For peer review only

Supplementary Figure 1: Proportion of third trimester pregnant women who received ANC from MTP during pre-lockdown and lockdown period (N=226)

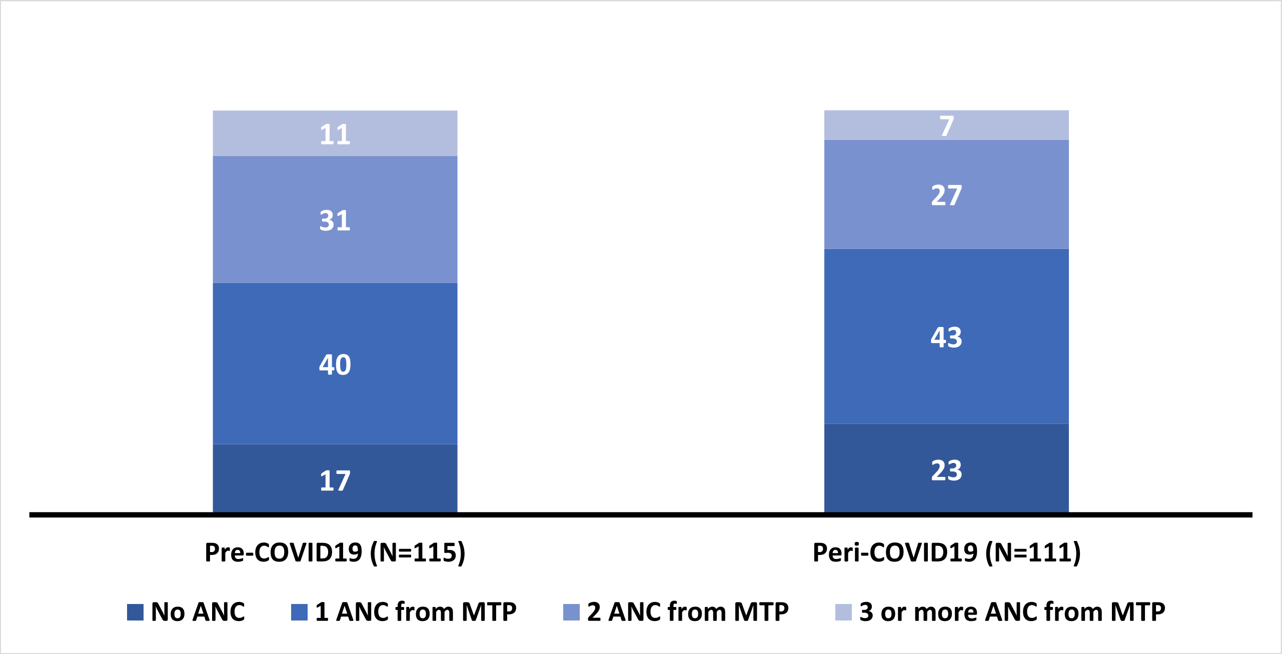

Supplementary Figure 2: Awareness and use of telemedicine services among the respondents (N=555)

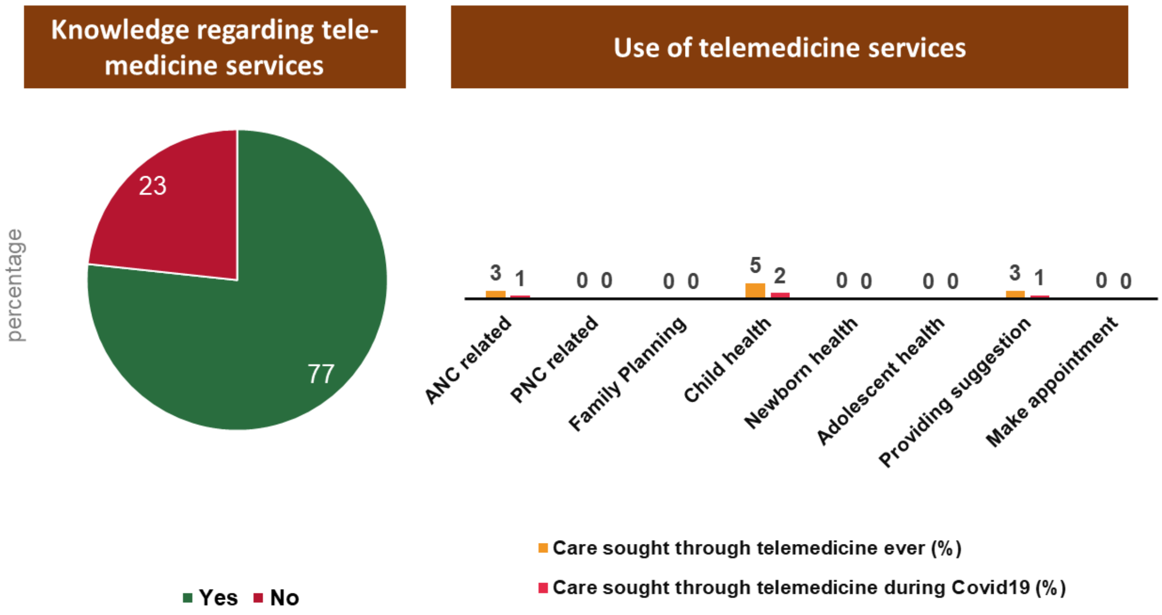

eview only

STROBE Statement—Checklist of items that should be included in reports of ***cross-sectional studies***

	Item No	Recommendation
**Title and abstract**	1	(*a*) Indicate the study's design with a commonly used term in the title or the abstract
		(*b*) Provide in the abstract an informative and balanced summary of what was done and what was found
**Introduction**		
Background/rationale	2	Explain the scientific background and rationale for the investigation being reported
Objectives	3	State specific objectives, including any prespecified hypotheses
**Methods**		
Study design	4	Present key elements of study design early in the paper
Setting	5	Describe the setting, locations, and relevant dates, including periods of recruitment, exposure, follow-up, and data collection
Participants	6	(*a*) Give the eligibility criteria, and the sources and methods of selection of participants
Variables	7	Clearly define all outcomes, exposures, predictors, potential confounders, and effect modifiers. Give diagnostic criteria, if applicable
Data sources/ measurement	8*	For each variable of interest, give sources of data and details of methods of assessment (measurement). Describe comparability of assessment methods if there is more than one group
Bias	9	Describe any efforts to address potential sources of bias
Study size	10	Explain how the study size was arrived at
Quantitative variables	11	Explain how quantitative variables were handled in the analyses. If applicable, describe which groupings were chosen and why
Statistical methods	12	(*a*) Describe all statistical methods, including those used to control for confounding
		(*b*) Describe any methods used to examine subgroups and interactions
		(*c*) Explain how missing data were addressed
		(*d*) If applicable, describe analytical methods taking account of sampling strategy
		(*e*) Describe any sensitivity analyses
**Results**		
Participants	13*	(a) Report numbers of individuals at each stage of study—eg numbers potentially eligible, examined for eligibility, confirmed eligible, included in the study, completing follow-up, and analysed
		(b) Give reasons for non-participation at each stage
		(c) Consider use of a flow diagram
Descriptive data	14*	(a) Give characteristics of study participants (eg demographic, clinical, social) and information on exposures and potential confounders
		(b) Indicate number of participants with missing data for each variable of interest
Outcome data	15*	Report numbers of outcome events or summary measures
Main results	16	(*a*) Give unadjusted estimates and, if applicable, confounder-adjusted estimates and their precision (eg, 95% confidence interval). Make clear which confounders were adjusted for and why they were included
		(*b*) Report category boundaries when continuous variables were categorized
		(*c*) If relevant, consider translating estimates of relative risk into absolute risk for a meaningful time period
Other analyses	17	Report other analyses done—eg analyses of subgroups and interactions, and sensitivity analyses

For peer review only

For peer review only - http://bmjopen.bmj.com/site/about/guidelines.xhtml

**Discussion**		
Key results	18	Summarise key results with reference to study objectives
Limitations	19	Discuss limitations of the study, taking into account sources of potential bias or imprecision. Discuss both direction and magnitude of any potential bias
Interpretation	20	Give a cautious overall interpretation of results considering objectives, limitations, multiplicity of analyses, results from similar studies, and other relevant evidence
Generalisability	21	Discuss the generalisability (external validity) of the study results
**Other information**		
Funding	22	Give the source of funding and the role of the funders for the present study and, if applicable, for the original study on which the present article is based

*Give information separately for exposed and unexposed groups.

Note: An Explanation and Elaboration article discusses each checklist item and gives methodological background and published examples of transparent reporting. The STROBE checklist is best used in conjunction with this article (freely available on the Web sites of PLoS Medicine at http://www.plosmedicine.org/, Annals of Internal Medicine at http://www.annals.org/, and Epidemiology at http://www.epidem.com/). Information on the STROBE Initiative is available at www.strobe-statement.org.

For peer review only

For peer review only - http://bmjopen.bmj.com/site/about/guidelines.xhtml

BMJ Open

Indirect effects of the early phase of the COVID-19 pandemic on the coverage of essential maternal and newborn health services in a rural sub-district in Bangladesh: results from a cross-sectional household survey

Journal:	*BMJ Open*
Manuscript ID	bmjopen-2021-056951.R2
Article Type:	Original research
Date Submitted by the Author:	09-Dec-2021
Complete List of Authors:	Mhajabin, Shema; International Centre for Diarrhoeal Disease Research Bangladesh, Maternal and Child Health<br>Hossain, Aniqa ; International Centre for Diarrhoeal Disease Research Bangladesh, biostatistics<br>Nusrat, Nowrin; International Centre for Diarrhoeal Disease Research Bangladesh<br>Jabeen, Sabrina; International Centre for Diarrhoeal Disease Research Bangladesh<br>Ameen, Shafiqul; International Centre for Diarrhoeal Disease Research Bangladesh<br>Banik, Goutom; International Centre for Diarrhoeal Disease Research Bangladesh, Maternal and Child Health Division ; International Centre for Diarrhoeal Disease Research Bangladesh,<br>Tahsina, Tazeen; International Centre for Diarrhoeal Disease Research Bangladesh, Maternal and Child Health Division<br>Ahmed , Anisuddin; ICDDRB<br>Sadeq-ur Rahman, Qazi; International Centre for Diarrhoeal Disease Research Bangladesh<br>Gurley, Emily; Johns Hopkins University<br>Bari, Sanwarul; International Centre for Diarrhoeal Disease Research Bangladesh<br>Chowdhury, Atique; ICDDRB<br>Arifeen, Shams E.; International Centre for Diarrhoeal Disease Research Bangladesh, Maternal and Child Health Division (MCHD)<br>Mehta, Rajesh; World Health Organization Regional Office for South-East Asia<br>Rahman, Ahmed ; International Centre for Diarrhoeal Disease Research Bangladesh; University of Edinburgh Division of Medical and Radiological Sciences
**Primary Subject Heading**:	Public health
Secondary Subject Heading:	Epidemiology, Evidence based practice, Global health
Keywords:	PUBLIC HEALTH, EPIDEMIOLOGY, COVID-19

SCHOLARONE™
Manuscripts

I, the Submitting Author has the right to grant and does grant on behalf of all authors of the Work (as defined in the below author licence), an exclusive licence and/or a non-exclusive licence for contributions from authors who are: i) UK Crown employees; ii) where BMJ has agreed a CC-BY licence shall apply, and/or iii) in accordance with the terms applicable for US Federal Government officers or employees acting as part of their official duties; on a worldwide, perpetual, irrevocable, royalty-free basis to BMJ Publishing Group Ltd ("BMJ") its licensees and where the relevant Journal is co-owned by BMJ to the co-owners of the Journal, to publish the Work in this journal and any other BMJ products and to exploit all rights, as set out in our licence.

The Submitting Author accepts and understands that any supply made under these terms is made by BMJ to the Submitting Author unless you are acting as an employee on behalf of your employer or a postgraduate student of an affiliated institution which is paying any applicable article publishing charge ("APC") for Open Access articles. Where the Submitting Author wishes to make the Work available on an Open Access basis (and intends to pay the relevant APC), the terms of reuse of such Open Access shall be governed by a Creative Commons licence – details of these licences and which Creative Commons licence will apply to this Work are set out in our licence referred to above.

Other than as permitted in any relevant BMJ Author's Self Archiving Policies, I confirm this Work has not been accepted for publication elsewhere, is not being considered for publication elsewhere and does not duplicate material already published. I confirm all authors consent to publication of this Work and authorise the granting of this licence.

For peer review only

Indirect effects of the early phase of the COVID-19 pandemic on the coverage of essential maternal and newborn health services in a rural sub-district in Bangladesh: results from a cross-sectional household survey

Shema Mhajabin[1], Aniqa Tasnim Hossain[1], Nowrin Nusrat[1], Sabrina Jabeen[1], Shafiqul Ameen[1], Goutom Banik[1], Tazeen Tahsina[1], Anisuddin Ahmed[1], Qazi Sadeq-ur Rahman[1], Emily S. Gurley[3], Sanwarul Bari[1], Md. Atique Iqbal Chowdhury[1], Shams El Arifeen[1≈], Rajesh Mehta[≈4], Ahmed Ehsanur Rahman[1&2≈*]

[≈] Joint co-senior author

[1]- International Centre for Diarrhoeal Disease Research, Bangladesh (icddr,b)

[2]- University of Edinburgh

[3]- Johns Hopkins University

[4]- World Health Organization

*Corresponding Author: Dr Ahmed Ehsanur Rahman, icddr,b, Mohakhali, Dhaka-1212, Bangladesh, email: ehsanur@icddrb.org, Mobile Number: +8801727010050

Keywords: COVID-19 pandemic, Lockdown, rural area, essential services, maternal health, newborn health

Total word count, excluding title page, abstract, references, figures, and tables: 4300

For peer review only

ABSTRACT

Objective: This paper presents the effect of the early phase of COVID-19 on the coverage of essential maternal and newborn health (MNH) services in a rural sub-district of Bangladesh.

Design: Cross-sectional household survey with random sampling

Setting: Baliakandi sub-district, Rajbari district, Bangladesh

Participants: Data were collected from women who were third-trimester pregnant during the early phase of the pandemic (111) and pre-pandemic period (115) to measure antenatal care (ANC) service coverage. To measure birth, postnatal care (PNC), and essential newborn service coverage (ENC), data were collected from women who had a history of delivery during the early phase of pandemic (163) and pre-pandemic period (166).

Exposure: Early phase of pandemic included a strict national lockdown between April-June 2020, and pre-pandemic was defined as August-October 2019.

Outcome of interest: Changes in the coverage of selected MNH services (ANC, birth, PNC, ENC) during the early phase of COVID-19 pandemic compared to the pre-pandemic period, estimated by two-sample proportion tests

Findings: Among women who were the third trimester pregnant during the early phase of the pandemic period, 77% (95% CI: 70-85) received at least one ANC from a medically trained provider (MTP) during the third trimester, compared to 83% (95% CI: 76-90) during the pre-pandemic period (p-value=0.33). Among women who gave birth during the early phase of the pandemic period, 72% (95% CI: 66-79) were attended by an MTP, compared to 63% (95% CI: 56-71) during the pre-pandemic period (p-value=0.08). Early initiation of breastfeeding was practiced among 38% (95% CI: 31-46) of the babies born during the early phase of the pandemic period. It was 37% (95% CI: 29-44) during the pre-pandemic period (p-value=0.81). The coverage of ANC, birth, PNC, and ENC did not differ by months of pandemic and pre-

For peer review only - http://bmjopen.bmj.com/site/about/guidelines.xhtml

pandemic period; only the coverage of at least one ANC from an MTP significantly differed among the women who were seven months pregnant during the early phase of pandemic (35%, 95% CI: 26-44) and pre-pandemic (49%, 95% CI: 39-58) (p-value=0.04).

Conclusion: The effect of the early phase of the pandemic including lockdown, on the selected MNH service coverage was null in the study area. The nature of the lockdown, the availability and accessibility of private-sector health services in that area, and the combating strategies at the rural level made it possible for the women to avail the required MNH services.

Strengths and limitations of this study

- This paper provides population-level service coverage during the early phase of pandemic, which national-level routine information systems cannot capture.
- Additionally, this paper compares the service coverage of the essential MNH services before and during the pandemic to understand the effect of the pandemic in rural areas where usually service coverage is less compared to urban areas.
- This study acknowledges the limitation of the cross-sectional design in inferring any causality and estimating the impact.
- This study adapted the questionnaire based on the standard Bangladesh Demographic Health Survey (BDHS), Bangladesh Maternal Mortality Survey (BMMS), and Multiple Indicator Cluster Surveys (MICS) tools which are validated and globally accepted.
- This study considered only 6 months of recall for assessing the coverage during the early phase of the pandemic period.

For peer review only - http://bmjopen.bmj.com/site/about/guidelines.xhtml

INTRODUCTION

The coronavirus disease 2019 (COVID-19), caused by the severe acute respiratory syndrome coronavirus 2 (SARS-CoV-2), has caused more than two hundred and forty-two million confirmed cases across 219 countries and territories and has taken around five million lives as of October 2021, making it the deadliest pandemic since the Spanish flu in 1918 [1 2]. However, this is only the tip of the iceberg. Any pandemic negatively impacts the social, economic, and political landscape globally, regionally, and nationally [3-6]. The unexpected health burden compels the policymakers to shift focus from routine healthcare and repurpose the existing resources to tackle the more ominous threats posed by the pandemic [7-9]. It is, therefore, likely that the morbidity and mortality due to other causes may arise during a pandemic, especially in countries with limited resources [10 11].

The risks of COVID-19 infection and its adverse clinical consequences are relatively low among children and the younger population [12 13]. A multi-country cohort study found that pregnant individuals with COVID-19 diagnosis had higher rates of adverse outcomes, including maternal mortality, preeclampsia, and preterm birth compared with pregnant individuals without COVID-19 diagnosis [14]. Mothers and newborns are also vulnerable to the indirect effects of the pandemic [15-18]. Focusing on the emergency response may result in the de-prioritization of essential health services, including maternal and newborn health (MNH). The availability of essential MNH services may become severely compromised due to the repurposing of limited resources, overwhelming demand of other emergency services, healthcare workers' absence and deaths, and reduced service hours or closure of health facilities [15 19-21]. Similarly, the access to and utilization of essential MNH services may be adversely affected by the hesitancy to seek care from health facilities due to the fear of contracting the virus and lack of reliable transportation due to the restrictive movement measures such as the lockdown [22 23]. A systematic review and meta-analysis of 40 studies reported that the global

For peer review only - http://bmjopen.bmj.com/site/about/guidelines.xhtml

burden of maternal deaths, stillbirth, and maternal depression have increased during the COVID-19 pandemic, with a high disparity between the high-income and low- and middle-income country settings [24]. Another study reported that the under-5 deaths increased by 45% per month and the maternal deaths have increased by 36% per month, across 118 countries due to the indirect effect of the COVID-19 pandemic using the survey data [15]. According to a United Nations report, COVID-19 may have contributed to an estimated 11,000 additional maternal deaths and 228,000 additional child deaths in South Asia in 2020 using routine health information data such as the District health information system (DHIS2) [16]. Such adverse indirect effects of the COVID-19 pandemic may abrade the major gains in improving MNH achieved over the past few decades.

On March 08, 2020, Bangladesh declared the first confirmed case of COVID-19, and the first death was reported on 18 March 2020. Since then, Bangladesh has reported more than seven hundred and seventy thousand confirmed cases, including more than 11,000 deaths [25]. The Government of Bangladesh declared "general leave" for a week on 26 March 2020 for interrupting the viral transmission chain and flattening the epidemic curve comparable to lockdown [26]. Later the "general leave" was extended for two months. In the beginning, all businesses except medical services were closed, and all kinds of movement except medical emergencies were restricted. The lockdown measures were attenuated in the second month of lockdown. A sharp decline was observed in the utilization of essential MNH services during the lockdown period (March-May, 2020) in Bangladesh using the DHIS2 [27 28]. For example, the study reported that there was a 41%, 52%, and 56% decrease in April 2020 compared to January 2020 for institutional normal deliveries in Upazila Health Complex (UHC), District Hospital (DHs), and tertiary hospitals. However, this was based on the DHIS2, a routine health information system, which collects utilization data from public health facilities and some selected private facilities. Therefore, this might not reflect the change in the coverage of

For peer review only - http://bmjopen.bmj.com/site/about/guidelines.xhtml

essential MNH services estimated with a true population-level denominator. This paper presents the effect of the early phase of the pandemic on the coverage of essential MNH services in Baliakandi, a rural sub-district in Bangladesh. This paper will further observe the differences in selected MNH indicators by month of the early phase of a pandemic.

METHODS

Study design

We conducted a cross-sectional household survey to estimate the coverage of essential MNH services during the early phase of pandemic and pre-pandemic periods. Antenatal care (ANC) from a medically trained provider (MTP), birth attended by an MTP, Essential Newborn Care (ENC), and Postnatal Care (PNC) from an MTP within 72 hours of birth were identified as tracers for the essential MNH services. As the local administration had implemented pandemic measures the April-June 2020 period was operationally defined as the early phase of the pandemic period. For identifying the pre-pandemic period (routine circumstances) we considered the following factors. China reported the first COVID-19 case in December 2019, and several global and national measures were taken between January and March 2020 and there may have been an impact on normal life and routine services. Before this, November 2019 – January 2020 period is the winter season in Bangladesh, and various cultural programs and family events are organized in this season, possibly leading to changes in the service use pattern. Hence, we considered August-October 2019 as the pre-pandemic period for comparison purposes.

Study setting

We conducted the study in a rural sub-district named Baliakandi, under the Rajbari district in Bangladesh. Since September 2017, the International Center of Diarrhoeal Disease Research, Bangladesh (icddr,b) has been conducting health and demographic surveillance in this area [29]. The surveillance covers 261 villages and a population of about 227,540 (in 2019). Through two

For peer review only - http://bmjopen.bmj.com/site/about/guidelines.xhtml

monthly household visits, the surveillance data collectors collect information on births, deaths, marriages, divorces, and migrations. In addition to these vital events, they also collect information on pregnancy and birth outcomes.

Study participants and Outcomes of interest

We used the icddr,b's Baliakandi surveillance data to generate the sampling frame based on specific inclusion criteria. Then, we used simple random sampling to select the required number of participants from each category (Supplementary figure 1). The women were randomly selected if they were into the following four groups:

i. Women who were the third trimester pregnant during April-June 2020 and gave birth in July 2020: 126 women were randomly selected to assess the effect of the early phase of a pandemic on ANC.
ii. Women who were the third trimester pregnant during August-October 2019 and gave birth in November 2019: 126 women were randomly selected to assess the effect of the pre-pandemic on ANC.
iii. Women who gave birth during April-June 2020: 178 women were randomly selected to assess the effect of the early phase of a pandemic on birth, PNC, and ENC.
iv. Women who gave birth in August-October 2019: 178 women were randomly selected to assess the effect of the pre-pandemic on birth, PNC, and ENC.

We calculated the sample size to detect a minimum effect size of 33 percentage points (relative) between the early phase of the pandemic and the pre-pandemic periods at 80% power (two-sided) and 5% error probability. We used the coverage estimates (rural) reported in 2017-18 Bangladesh Demographic and Health Survey (BDHS) for the selected essential MNH services as proxies for the pre-pandemic status [30]. The coverage estimates (rural) for the selected MNH services for the pandemic period were collected using DHIS2 data. The unadjusted sample size

For peer review only - http://bmjopen.bmj.com/site/about/guidelines.xhtml

was adjusted for a non-response rate of 10% and the clustering effect with a 1.10 design effect. Details regarding the sample size calculation for each of the essential MNH services are presented in (Supplementary Table 1).

Data collection tools

We used an interviewer-administered structured questionnaire to interview the eligible respondents. The questions were primarily adopted from the BDHS, Bangladesh Maternal Mortality Survey (BMMS), and Multiple Indicator Cluster Survey (MICS) [30-32]. The questionnaire consisted of seven main sections: household information, socio-economic status, respondent's background, pregnancy, birth, and postnatal care, ENC, care-seeking during emergencies, telemedicine service, and COVID-19 related knowledge and experiences. We translated the questionnaire into Bangla and pre-tested it before finalization.

Training and data collection

We recruited fifteen data collectors and two field supervisors from the local communities who were familiar with the local context, culture, and dialect. The data collection team received 5-days of face-to-face training, which was followed by two days of field testing. The training was conducted by the study investigators and other master trainers with special expertise and experience in household surveys.

Data were collected between 7 November to 25 December 2020. The field supervisors revisited a random sample of 5% of households to ensure quality. In addition, the field supervisors conducted unscheduled visits to observe the interviews and monitor the quality of data collection. Weekly meetings were organized to solve any emerging issues related to data collection. All questionnaires were manually reviewed by the field supervisors and the field research managers before sending for data entry.

For peer review only - http://bmjopen.bmj.com/site/about/guidelines.xhtml

We maintained social distancing and followed the national infection prevention and control guidelines during training and data collection [33]. All interviews were conducted outdoors. The data collection team received appropriate personal protective equipment such as masks, gloves, hand sanitizers, aprons, and face shields. Also, we distributed re-usable facemasks to the respondents to wear during the interviews.

Data analysis plan

We used the statistical software package STATA version 14.2 (StataCorp, College Station, TX) for data analysis [34]. At first, descriptive statistics were used to report the background characteristics of the respondents. We used the principal component analysis to generate an asset score for each household [35]. The asset scores were used to rank the households into quintiles. We reported the coverage of selected essential MNH services for the early phase of the pandemic and pre-pandemic periods separately with 95% confidence intervals (CI). Additionally, the service coverage of selected MNH indicators was presented by months of the early phase of pandemic and pre-pandemic with 95% CI. The operational definitions of each of the indicators are presented in supplementary table 2, and the definition of providers are presented in supplementary table 3. Two sample proportion tests were conducted to explore the difference in the coverage of selected essential MNH services between the early phase of the pandemic and pre-pandemic periods. We reported no significant difference at $p<0.05$.

Ethical approval and consent to participate

We obtained the ethical approval to conduct the study from the Institutional Review Board of icddr,b (PR-20085). Written and informed consent was sought from each study participant before the interview. In the case of respondents who cannot read or write, we obtained audio-recorded verbal informed consent.

For peer review only - http://bmjopen.bmj.com/site/about/guidelines.xhtml

Patient and public involvement

There was no patient or public involvement in the study.

RESULTS

We interviewed 115 women (non-response rate 9%) who were third-trimester pregnant during the pre-pandemic period and 111 women (non-response rate 12%) who were third-trimester pregnant during the early phase of the pandemic period. We also interviewed 166 women (non-response rate 7%) who gave birth during the pre-pandemic period and 163 women (non-response rate 9%) who gave birth during the early phase of the pandemic period. Table-1 describes the background characteristics of each group of respondents. Approximately one-third of the women were aged 20-24 years. Around half of them had incomplete secondary education (6-9 years of schooling), and one-fourth had complete secondary or higher education (10 or more years of schooling). Most of the respondents were Muslim. Approximately 40% of the women were primigravidae (first-time pregnant). Less than 10% of them were engaged in any income-generating activities. No notable difference was observed in characteristics across the groups of respondents.

For peer review only

For peer review only - http://bmjopen.bmj.com/site/about/guidelines.xhtml

Table 1: Background characteristics of the respondents; presented in column percentage

Background characteristics	Women who were third trimester pregnant		Women who had a history of birth outcome	
	Pre-pandemic	Early phase of pandemic	Pre-pandemic	Early phase of pandemic
	N=115	N=111	N=166	N=163
	%	%	%	%
**Age**				
14 and below	0	1	0	0
15-19	23	23	14	22
20-24	31	36	33	37
25-29	21	25	26	22
30-34	19	13	22	12
35+	6	2	5	7
**Education**				
No education	3	1	5	3
Primary incomplete[1]	9	10	9	11
Primary complete[2]	7	9	12	11
Secondary incomplete[3]	56	48	52	52
Secondary complete or higher[4]	25	32	22	23
**Religion**				
Muslim	83	88	89	91
Hinduism/ Buddhism/ Christianity	17	12	11	9
**Parity**				
Primiparous	41	41	32	44
Multiparous	59	59	68	56
**Occupation**				
Housewife	96	95	95	90
Engaged in any income-generating activity	4	5	5	10
**Wealth quintile**				
Lowest	22	14	15	15
Second	23	19	24	24
Middle	25	23	25	23
Fourth	13	23	18	23
Highest	17	21	18	15
**Total**	100	100	100	100

[1]: 1-4 years of schooling
[2]: 5 years of schooling
[3]: 6-9 years of schooling
[4]: 10 or more years of schooling

For peer review only - http://bmjopen.bmj.com/site/about/guidelines.xhtml

Figure -1 summarises the coverage of essential MNH services during pre-pandemic and early phases of pandemic periods. Among women who were third-trimester pregnant during the early phase of the pandemic period, 77% (95% CI: 70-85) received at least one ANC from an MTP during the third trimester, which was 83% (95% CI: 76-90) during the pre-pandemic period (p-value=0.33). Similarly, among women who were third-trimester pregnant during the early phase of the pandemic period, 81% (95% CI: 74-88) received at least one ANC from a health facility during the third trimester, which was 83% (95% CI: 76-90) during the pre-pandemic period (p-value=0.77).

Among women who gave birth during the pandemic period, 72% (95% CI: 66-79) were attended by an MTP whereas only 63% (95% CI: 56-71) women were attended by an MTP during the pre-pandemic period (p-value=0.08). The coverage of facility-birth was 65% (95% CI: 58-72) during the pandemic period and 55% (95% CI: 47-62) during the pre-pandemic period (p-value=0.06). The coverage of at least one PNC from an MTP was 58% (95% CI: 51-60) during the pandemic period and 52% (95% CI: 45-60) during the pre-pandemic period (p-value=0.28).

Among babies born during the pandemic period, the coverage of clean cord cutting practice was 65% (95% CI: 58-72) whereas only 59% (95% CI: 52-67) of babies born during the pre-pandemic period had clean cord-cutting (p-value=0.26). The 7.1% chlorhexidine was applied to 16% (95% CI: 10-22) of babies born during the pandemic period, which was 18% (95% CI: 12-24) during the pre-pandemic period (p-value=0.61). Early initiation of breastfeeding was practiced among 38% (95% CI: 31-46) of the babies born during the pandemic period whereas it was 37% (95% CI: 29-44) during the pre-pandemic period (p-value=0.81).

Figure 1: Coverage of essential MNH services during the pre-pandemic and the pandemic periods, presented in percentage with 95% confidence intervals

For peer review only - http://bmjopen.bmj.com/site/about/guidelines.xhtml

Figure -2 illustrates the coverage of ANC received in the third trimester of pregnancy by months during the pandemic and pre-pandemic period. During the pandemic period, only 35% (95% CI: 26-44) women received at least one ANC from an MTP in their 7th month of pregnancy (April 2020) whereas it was 49% (95% CI: 39-58) for pre-pandemic (August 2019) (p-value=0.04). Similarly, only 36% (95% CI: 27-45) women received at least one ANC from a health facility, and 32% (95% CI: 23-40) women received at least one ANC from a private health facility during the 7th month of pregnancy (April 2020). However, these estimates were significantly lower than that of the pre-pandemic period (August 2019) (p-value=0.05). No notable difference was observed in the ANC coverage during the 8th and 9th months of pregnancy between the pandemic and pre-pandemic periods (p-value>0.05).

Figure 2: Coverage of ANC received in the third trimester during the pre-pandemic and the pandemic periods; presented in percentages by months of pregnancy

Among the women who were third-trimester pregnant, 23% did not receive any ANC from an MTP during the pandemic period whereas only 17% did not receive any ANC from an MTP during the pre-pandemic period (p-value=0.33) (Supplementary figure 2). During the pandemic period, only 43% of third-trimester pregnant women received one ANC from an MTP, but during the pre-pandemic, it was only 40% (p-value=0.62) We did not observe any notable difference (statistically significant at p<0.05) in the above-mentioned distribution of ANC status between the pandemic and the pre-pandemic periods. The care-seeking for ANC using telemedicine services were very low and not different (p-value=0.99) between pandemic and pre-pandemic period (Supplementary figure 3).

Table 2 presents the type of health care provider from who the women received ANC during their third trimester of pregnancy. Among the women who were third-trimester pregnant during the pandemic period, 11% received ANC from a nurse/midwives/paramedic whereas 20% received ANC from these providers during the pre-pandemic period (p-value=0.06). No notable

For peer review only - http://bmjopen.bmj.com/site/about/guidelines.xhtml

difference (statistically significant at p<0.05) was observed in the above-mentioned distribution of ANC providers between the pandemic and the pre-pandemic periods.

Table 2: Type of provider from who ANC was received in the third trimester during the pre-pandemic and early phase of pandemic periods; presented in percentages, multiple responses considered

Type of providers	Pre-pandemic (95% CI)	Early phase of pandemic (95% CI)	P-value
Doctor	80 (73-87)	77 (70-78)	0.64
Nurse/midwives/paramedic	20 (13-27)	11 (5-17)	0.06
FWV	11 (6-17)	7 (2-12)	0.29
CSBA	0	0	
SACMO/MA/CHCP/HA/FWA	8 (3-13)	13 (6-19)	0.24
NGO Worker/ TBA	2 (0-4)	6 (2-11)	0.08

*FWV: Family welfare visitor; CSBA: Community skilled birth attendant; SACMO: Sub-assistant community medical officer; MA: Medical assistant; CHCP: Community health care provider; HA: Health assistant; FWA: Family welfare assistant; TBA: Traditional birth attendant

Figure-3 presents the coverage of birth attended by an MTP, birth in a health facility, birth in a private health facility, and birth through C-sections. Among women who gave birth during the pandemic period, the coverage of birth attended by an MTP was around 70% across all three months. Similarly, the coverage of facility-birth was more than 60% across all three months. Around 50% of birth occurred in a private health facility, and the rates were consistent across the three months. The coverage of birth attended by an MTP was 73% (95% CI: 59-85) in April 2020 whereas it was only 64% (95% CI: 46-79) in August 2019 (p-value=0.46). We did not observe any notable difference (statistically significant at p<0.05) in the coverage of birth attended by an MTP, birth in a health facility, birth in a private health facility, and birth through C-sections during the pandemic and pre-pandemic periods for any of the reporting months.

Figure 3: Coverage of births attended by a medically trained provider, birth in a health facility, birth in a private health facility, and birth through C-sections during the pre-pandemic and pandemic periods; presented in percentages by calendar months

For peer review only - http://bmjopen.bmj.com/site/about/guidelines.xhtml

Table 3 presents the type of health care providers attending the births which were conducted during the pre-pandemic and the pandemic period. Among the women who gave birth during the pandemic period, 53% were attended by a doctor and 71% by a nurse/midwife/paramedic. Among the women who gave birth during the pre-pandemic period, 49% were attended by a doctor and 62% by a nurse/midwife/paramedic. No notable difference (statistically significant at $p<0.05$) was observed in the distribution of providers attending births between the pandemic and the pre-pandemic periods.

Table 3: Type of provider attending the births during the pre-pandemic and early phase of pandemic periods; presented in percentages

Type of providers	Pre-pandemic (95% CI)	Early phase of pandemic (95% CI)	P-value
Doctor	49 (41-56)	53 (46-61)	0.41
Nurse/midwives/paramedic	62 (55-69)	71 (64-78)	0.08
FWV	0	1 (0-2)	0.31
CSBA	2 (-1,4)	0	0.32
SACMO/MA/CHCP/HA/FWA	4 (1-6))	2 (0-4)	0.32
NGO Worker/ TBA	40 (33-48)	35 (28-41)	0.31

*FWV: Family welfare visitor; CSBA: Community skilled birth attendant; SACMO: Sub-assistant community medical officer; MA: Medical assistant; CHCP: Community health care provider; HA: Health assistant; FWA: Family welfare assistant; TBA: Traditional birth attendant

Figure-4 shows the coverage of PNC from an MTP, PNC from a health facility, and PNC from a private health facility during the pre-pandemic and the pandemic periods. Among women who gave birth during the pandemic period, the coverage of PNC by an MTP within 72 hours of birth was around 60% across all three months. Similarly, the coverage of PNC in a health facility facility-birth was around 55% across all three months. The coverage of PNC by an MTP was 57% (95% CI: 42-71) in April 2020 whereas it was only 53% (95% CI: 35-70) in August 2019 (p-value=0.79). We did not observe any notable difference (statistically significant at $p<0.05$) in the coverage of PNC by an MTP, PNC in a health facility, and PNC in a private health facility during the pandemic and pre-pandemic periods for any of the reporting months.

For peer review only - http://bmjopen.bmj.com/site/about/guidelines.xhtml

Figure 4: Coverage of PNC by a medically trained provider, PNC in a health facility, PNC in a private health facility among births occurred during the pre-pandemic and pandemic periods; presented in percentages by calendar months

Figure-5 presents the coverage of different components of ENC among the newborns during the pre-pandemic and the pandemic periods. Among babies born at the early phase of the pandemic (April 2020), the coverage of early initiation of breastfeeding was 24% (95% CI: 13-39), which was much lower than that of the remaining months (May-June 2020) during the pandemic period and the pre-pandemic period (p-value=0.37). No notable difference (statistically significant at $p<0.05$) was observed in the coverage of other components of ENC between the pandemic and the pre-pandemic periods by calendar months.

Figure 5: Coverage of different components of ENC during the pre-pandemic and pandemic periods; presented in percentages by calendar months

DISCUSSION

The healthcare-seeking experience for ANC, birth, PNC, and ENC during the early phase of the COVID-19 pandemic in rural Bangladesh was not found affected in this study. We found that the coverage of selective essential MNH services during the early phase of the pandemic was not different from that of the pre-pandemic period in this rural sub-district in Bangladesh. However, national-level data from the routine health system of Bangladesh (DHIS2) showed a substantial decline in the utilization of essential MNH services [27]. The possible explanations for such contradicting findings are summarized under two broad themes, i.e., how the service utilization was restored (health system response and community coping strategy and resilience) and why we did not see any difference in the service utilization (private-sector coverage, enforcement of lockdown measures, and measurement issues in the routine health information system) are discussed below.

For peer review only - http://bmjopen.bmj.com/site/about/guidelines.xhtml

Health systems responses:

Several health systems initiatives taken by the Government of Bangladesh might have contributed to overcoming the initial shock of the pandemic and preventing a substantial decline in the coverage of key MNH services. In March 2020, the Government developed the Bangladesh Preparedness and Response Plan for COVID-19, which also emphasized the importance of maintaining essential health and nutrition services during pandemic [36]. After the initial focus on COVID-19-specific preparation and responses, several initiatives were taken to promote the availability and readiness of essential MNH services. In May 2020, the Ministry of Health, with technical inputs from the development partners and professional societies, developed the "National Guideline for providing essential Maternal, Newborn and Child Health Services in the context of COVID-19". The prime objective of this guideline was to inform the health care providers and facility- and district-level managers of the importance of providing essential MNCH services in the context of COVID-19 [37]. It also provided specific guidance on continuing the essential services safely by maintaining physical distancing, using personal protective equipment, and promoting infection prevention and control practices. The Ministry of Health organized a series of online sessions to sensitize the facility- and district-level managers. Afterward, face-to-face training was organized for the frontline health care workers responsible for providing MNH services. Then national-level policymakers organized virtual meetings with facility-, district- and regional-level managers to review the MNH utilization data in respective health facilities and discuss strategies for overcoming the bottlenecks, barriers, and operational challenges. Special initiatives were also taken for establishing functional triage, isolation unit for suspected COVID-19 patients, and red zone for confirmed COVID-19 patients at referral facilities, which promoted a safer environment at the facility for receiving routine services and might have contributed to restoring the public confidence for availing essential MNH services. In June 2020, the Government of Bangladesh recruited and

For peer review only - http://bmjopen.bmj.com/site/about/guidelines.xhtml

deployed 2000 doctors and 5000 nurses for COVID-19 response [38]. The influx of additional human resources helped re-deploying the MNH services to their original duties and minimize the impact of COVID-19 on essential services. These national efforts might have positively influenced minimizing the disruption in the availability and provision of essential services in our study settings. According to the national routine health information system, there was little difference in the utilization of essential MNH services from public health facilities in the Baliakandi sub-district during the early phase of pandemic period [27]. This is particularly applicable for the Upazila Health Complex, which is the primary reference point for all health centers in the study site. We also did not observe any notable difference in the utilization of MNH services from the district hospital during that period, which is the primary reference point for all the Upazila Health Complexes in that district [27]. It indicates that most of the public health referral facilities continued providing routine services despite prioritizing the COVID-19 specific services. Since the referral facilities have more human resources than health centers, which have only one to two dedicated providers, it was easier for them to adopt an alternate approach for continuing essential MNH services.

Community coping strategy and resilience:

Bangladesh is one of the most vulnerable countries to climate variability and fluctuation [39 40]. A quarter of the land in Bangladesh is barely above sea level and is susceptible to the effects of seawater rise and increased salinity due to climate change. Bangladesh also experiences other forms of natural disasters like floods and cyclones quite frequently [41]. The people in Bangladesh have learned to cope with the emergencies and show resilience by focusing on rebuilding after the initial shock, mostly through local-level initiatives [42 43]. Awareness regarding the importance of receiving MNH services from appropriate places and providers has increased substantially and consistently over the past couple of decades, even in the rural context. Hence the awareness and the resilient nature of local communities might have

For peer review only - http://bmjopen.bmj.com/site/about/guidelines.xhtml

contributed to finding ingenious ways and coping strategies to avail the essential MNH service during the pandemic overcoming the challenges imposed by the lockdown.

Private sector coverage:

Bangladesh has a pluralistic health system constituted by the public sector, for-profit formal private sector, and informal care providers [44 45]. Although the Government of Bangladesh has a large network of health infrastructure throughout the country, the majority of MNH services are provided by the poorly regulated for-profit private sector and unregulated informal care providers. According to 2017-18 BDHS, more than two-thirds of the ANC and facility-births took place in private health facilities [30]. In the past decade, the coverage of births in private health facilities has increased by fourfolds [30 46]. A similar trend was also observed for other essential MNH services such as ANC, PNC, and ENC. Although almost all the public health facilities report to DHIS2, very few private hospitals and private practitioners report to DHIS2. This disconnect between the larger market share of the private sector and disproportionately minor contribution in the DHIS2-based reporting system may explain our study findings. We did not observe any notable change in the coverage of essential MNH services during the early phase of the pandemic period. In contrast, the DHIS2-based reporting system was indicating a substantial decline in service utilization nationally. In our study site, around 55% of births took place in the health facilities during the pre-pandemic period, of which about 80% were in private health facilities. The condition did not change during the pandemic period as 65% of births took place in health facilities, of which more than 80% were in private health facilities. We observed similar patterns for ANC and PNC services. In the rural sub-district where we conducted the study, there are three for-profit private health facilities that provide MNH services [29]. In addition, there are 16 for-profit private facilities and two non-profit NGO clinics in the district headquarter, and many of them offer MNH services. Therefore, the prioritization of emergency services related to COVID-19 exclusively through the public health facilities

For peer review only - http://bmjopen.bmj.com/site/about/guidelines.xhtml

may explain the initial decline in utilization of essential MNH services in those facilities, but the overwhelming majority in the market share the private sector and the continuation of the routine services may have contributed to sustaining the coverage essential MNH services in the early stage of the pandemic.

Enforcement of lockdown measures:

The Government of Bangladesh imposed a nationwide restriction of movements in late March 2020 to interrupt the pathways of COVID-19 transmission [26 47 48]. However, the Government declared it as 'general leave' instead of lockdown, which undermined the importance of adhering to the recommended measures among the mass population. At the beginning of the lockdown, all the government and non-government offices, factories, and academic institutes were asked to shut down. A large proportion of city dwellers traveled back to their village homes, maintaining very little social distancing and other infection prevention and control measures [47]. After a couple of weeks, some government offices, and factories, including all readymade garment factories, were allowed to resume their operations, forcing many people to return to their workplace. Hence, the restrictions regarding inter-district and within-district movements gradually weaned off, and the public transportation options slowly resumed back with some infection prevention and control regulations [26]. Moreover, the lockdown measures were less coercively imposed in rural settings as the transportation services were reasonably available, allowing people to move within the sub-district and to the district headquarter when required. In our study, less than 5% of the respondents mentioned lack of transportation as a barrier to care-seeking, and less than 10% reported spending extra money for the vehicle rent while receiving essential MNH services during the lockdown period. Based on a national survey, around two-thirds of the pregnant women in rural settings can reach a private health facility or provider within five-kilometer of travel distance [30]. It implies that people could avail

For peer review only - http://bmjopen.bmj.com/site/about/guidelines.xhtml

the essential MNH services from nearby private health facilities when the services were temporarily disrupted in public health facilities in the early stage of the pandemic.

Measurement issues:

As mentioned earlier, there are limitations in estimating the coverage of health services through the routine health information system in Bangladesh. The Government of Bangladesh has made a substantial investment in strengthening the routine health information system by introducing DHIS2 in 2009 [44]. All the public health facilities and a few private health facilities report their health service use data to DHIS2. Although MNH is a priority in the DHIS2-based reporting system in Bangladesh, the system's reach is not enough to accurately capture population-level denominators such as live births, which are essential for measuring the coverage of MNH services. In 2019, DHIS2 reported a total of 931,723 live births in Bangladesh [27]. Based on the crude birth rate reported in 2017-18 BDHS, the size of the annual birth cohort is more than 3 million in Bangladesh [30]. Similarly, DHIS2 reported that around 655,542 women received 4 ANC from the reporting facilities in 2019 [27]. However, an estimated 1,410,000 (47% of 3 million births) women received four or more ANC based on the national survey estimate of [30]. Moreover, there are issues with the accuracy and quality of reporting in the routine health information systems, including DHIS2, particularly in low- and middle-income country settings [49-52]. Due to these gaps in accurately capturing the denominators and numerators through the DHIS2-based reporting system, the decline observed in the utilization of essential MNH services in public health facilities in the early stage of the pandemic may not reflect the true change in the coverage of relevant services.

Secondly, there were gaps and delays in the documentation and reporting of routine services in the initial phase of the pandemic, particularly during the lockdown period due to the reshaping of the health care providers' responsibilities and health systems responses. The Government of Bangladesh prepared the National Preparedness and Response Plan for the pandemic and

For peer review only - http://bmjopen.bmj.com/site/about/guidelines.xhtml

repurposed the existing infrastructure, human resources, and logistics to prevent and treat COVID-19 [36]. Initially, triage was introduced in all public health facilities, and some hospitals were dedicated to treating COVID-19. Additionally, a COVID-19 dashboard has been developed and is updated daily [25]. This portal displays data on the number of laboratories testing for COVID-19 and the number of health facilities treating COVID-19 patients. The dashboard also displays information on the number of tests performed, number of cases identified, number of people in quarantine and isolation, and number of deaths daily. It also provides an overview of the stock availability of COVID-19 related logistics of all health facilities. The frontline health care workers, facility and district level managers, and national policymakers prioritized capturing and reporting of COVID-19 related data to monitor the relevant implementation and response status. Hence, the introduction and emphasis on COVID-19 related services, documentation, and reporting might have overwhelmed the health care providers and the health systems resulting in the initial gaps and delays of reporting other routine services, including MNH. For example, we accessed DHIS2 in June 2020 and observed a sharp decline in the utilization of essential MNH services from public health facilities in March-May 2020 [27]. We accessed DHIS2 in September 2020 and found that the service utilization numbers were updated for the same time March-May 2020, resulting in a much smaller decline than the pre-COVID-19 period. Therefore, the true impact on the coverage of essential MNH services might be less than what was originally observed based on DHIS2 utilization data in the early phase of the pandemic.

Conclusion

Our study findings reported no significant changes in the overall coverage of MNH services in the rural area of Bangladesh. Moreover, the changes were not even significant for monthly comparison during the early phase of pandemic and pre-pandemic periods. It highlights the importance of expanding and reinforcing the routine health information system to cover the

For peer review only - http://bmjopen.bmj.com/site/about/guidelines.xhtml

information from the private health facilities and community services for comprehensive monitoring of essential services, particularly during the emergency response. The results also reveal the strength of local level coping strategy and coping strategy to overcome the immediate shock.

For peer review only

For peer review only - http://bmjopen.bmj.com/site/about/guidelines.xhtml

REFERENCE

1. Worldometers. COVID-19 CORONAVIRUS PANDEMIC 2021 [Available from: https://www.worldometers.info/coronavirus/ accessed April 28 2021.
2. (WHO) WHO. WHO Coronavirus (COVID-19) Dashboard 2021 [Available from: https://covid19.who.int/ accessed April 28 2021.
3. Davies SE. National security and pandemics. *UN Chronicle* 2013;50(2):20-24.
4. Drake TL, Chalabi Z, Coker R. Cost-effectiveness analysis of pandemic influenza preparedness: what's missing? *Bulletin of the World Health Organization* 2012;90:940-41.
5. Tisdell CA. Economic, social and political issues raised by the COVID-19 pandemic. *Economic analysis and policy* 2020;68:17-28.
6. Qiu W, Rutherford S, Mao A, et al. The pandemic and its impacts. *Health, culture and society* 2017;9:1-11.
7. Chowdhury A. Repurposing innovations: Learning from Bangladesh's rapid response to the pandemic', 2020.
8. Ahmed T, Rahman AE, Amole TG, et al. The effect of COVID-19 on maternal newborn and child health (MNCH) services in Bangladesh, Nigeria and South Africa: call for a contextualised pandemic response in LMICs. *International Journal for Equity in Health* 2021;20(1):1-6.
9. Her M. Repurposing and reshaping of hospitals during the COVID-19 outbreak in South Korea. *One Health* 2020;10:100137.
10. Wilhelm JA, Helleringer S. Utilization of non-Ebola health care services during Ebola outbreaks: a systematic review and meta-analysis. *Journal of global health* 2019;9(1)
11. Jones SA, Gopalakrishnan S, Ameh CA, et al. 'Women and babies are dying but not of Ebola': the effect of the Ebola virus epidemic on the availability, uptake and outcomes of maternal and newborn health services in Sierra Leone. *BMJ global health* 2016;1(3):e000065.
12. Sinha IP, Harwood R, Semple MG, et al. COVID-19 infection in children. *The Lancet Respiratory Medicine* 2020
13. zazhi ZlxbxzzZl. The epidemiological characteristics of an outbreak of 2019 novel coronavirus diseases (COVID-19) in China. *Novel, Coronavirus Pneumonia Emergency Response Epidemiology* 2020;41(2):145.
14. Villar J, Ariff S, Gunier RB, et al. Maternal and Neonatal Morbidity and Mortality Among Pregnant Women With and Without COVID-19 Infection: The INTERCOVID Multinational Cohort Study. *JAMA Pediatrics* 2021;175(8):817-26. doi: 10.1001/jamapediatrics.2021.1050
15. Roberton T, Carter ED, Chou VB, et al. Early estimates of the indirect effects of the COVID-19 pandemic on maternal and child mortality in low-income and middle-income countries: a modelling study. *The Lancet Global Health* 2020;8(7):e901-e08.
16. UNICEF. Direct and indirect effects of the COVID-19 pandemic and response in South Asia: UNICEF, 2021.
17. Busch-Hallen J, Walters D, Rowe S, et al. Impact of COVID-19 on maternal and child health. *The Lancet Global Health* 2020;8(10):e1257.
18. Ilerioluwa Oke G, Ebuka Elebesunu E, Ihekweazu V. Impact of COVID-19 Pandemic on Maternal and Child Health. *Modern Care Journal* 2020;17(4)
19. Elston JW, Cartwright C, Ndumbi P, et al. The health impact of the 2014-15 Ebola outbreak. *Public health* 2017;143:60-70. doi: 10.1016/j.puhe.2016.10.020 [published Online First: 2017/02/06]
20. Semaan A, Audet C, Huysmans E, et al. Voices from the frontline: findings from a thematic analysis of a rapid online global survey of maternal and newborn health professionals facing the COVID-19 pandemic. *BMJ Glob Health* 2020;5(6) doi: 10.1136/bmjgh-2020-002967 [published Online First: 2020/06/27]

For peer review only - http://bmjopen.bmj.com/site/about/guidelines.xhtml

21. Hassanian-Moghaddam H, Zamani N, Kolahi A-A. COVID-19 pandemic, healthcare providers' contamination and death: an international view. *Critical Care* 2020;24(1):208. doi: 10.1186/s13054-020-02938-y
22. Pant S, Koirala S, Subedi M. Access to maternal health services during COVID-19. *Europasian Journal of Medical Sciences* 2020;2(2):48-52.
23. Temesgen K, Wakgari N, Debelo BT, et al. Maternal health care services utilization amidstCOVID-19 pandemic in West Shoa zone, central Ethiopia. *PloS one* 2021;16(3):e0249214.
24. Chmielewska B, Barratt I, Townsend R, et al. Effects of the COVID-19 pandemic on maternal and perinatal outcomes: a systematic review and meta-analysis. *The Lancet Global Health* 2021
25. Director Genereal of Health Services B. Coronavirus COVID-19 Dashboard, 2020 2020 [Available from: http://dashboard.dghs.gov.bd/webportal/pages/covid19.php accessed April 28 2021.
26. Shammi M, Bodrud-Doza M, Islam ARMT, et al. Strategic assessment of COVID-19 pandemic in Bangladesh: comparative lockdown scenario analysis, public perception, and management for sustainability. *Environment, Development and Sustainability* 2020:1-44.
27. Director Genereal of Health Services B. DHIS2 Central Database (Upazila level and Above) 2021 [Available from: https://dghs.gov.bd/index.php/en/component/content/article?id=456 accessed April 28 2021.
28. Wangmo S, Sarkar S, Islam T, et al. Maintaining essential health services during the pandemic in Bangladesh: the role of primary health care supported by routine health information system. *WHO South-East Asia Journal of Public Health* 2021;10(3):93.
29. CHAMPS. CHAMPS in Bangladesh 2021 [cited 2021. Available from: https://champshealth.org/sites-bangladesh/ accessed 01 June 2021.
30. National Institute of Population Research and Training (NIPORT), ICF International. Bangladesh Demographic and Health Survey 2017-18: Key indicators. Dhaka, Bangladesh, and Rockville, Maryland, USA, 2019.
31. National Institute of Population Research and Training (NIPORT), International Centre for Diarrhoeal Disease Research Bangladesh (icddr b, MEASURE Evaluation. Bangladesh Maternal Mortality and Health Care Survey 2016: Preliminary Report. Dhaka, Bangladesh and Chapel Hill, NC, USA, 2017.
32. Unicef. MULTIPLE INDICATOR CLUSTER SURVEY 2016: NBS, UNICEF, 2018.
33. Directorate General of Health Services (DGHS) MoHaFWM, Bangladesh. NATIONAL GUIDELINE ON INFECTION PREVENTION AND CONTROL IN HEALTHCARE SETTINGS WITH ADDITIONAL MEASURES FOR COVID-19: Directorate General of Health Services (DGHS), Ministry of Health and Family Welfare (MOHFW), Bangladesh, 2020.
34. StataCorp. STATA version 14.2 2021 [updated 2021; cited 2021. Available from: https://www.stata.com/ accessed 01 June, 2021 2021.
35. Wold S, Esbensen K, Geladi P. Principal component analysis. *Chemometrics and intelligent laboratory systems* 1987;2(1-3):37-52.
36. Ministry of Health and Family Welfare (MoH&FW). National Preparedness and Response Plan for COVID-19 Bangladesh: Directorate General of Health Services, Health Service Division, Ministry of Health and Family Welfare, Bangladesh, 2020.
37. MNCAH D. National Guideline for providing essential Maternal, Newborn and Child Health Services in the context of COVID-19. May 2020
38. bdnews24.com. Bangladesh moves to recruit 2,000 doctors, 5,000 nurses amid virus outbreak. May 2020
39. Naser MM, Swapan MSH, Ahsan R, et al. Climate change, migration and human rights in Bangladesh: Perspectives on governance. *Asia Pacific Viewpoint* 2019;60(2):175-90.
40. Shahid S. Probable impacts of climate change on public health in Bangladesh. *Asia Pacific Journal of Public Health* 2010;22(3):310-19.
41. Rahman R, Salehin M. Flood risks and reduction approaches in Bangladesh. Disaster risk reduction approaches in Bangladesh: Springer 2013:65-90.

For peer review only - http://bmjopen.bmj.com/site/about/guidelines.xhtml

42. Parvin GA, Shimi AC, Shaw R, et al. Flood in a changing climate: The impact on livelihood and how the rural poor cope in Bangladesh. *Climate* 2016;4(4):60.
43. Younus MAF, Kabir MA. Climate change vulnerability assessment and adaptation of Bangladesh: Mechanisms, notions and solutions. *Sustainability* 2018;10(11):4286.
44. Khan MAH, Azad AK, de Oliveira Cruz V. Bangladesh's digital health journey: reflections on a decade of quiet revolution. *WHO South-East Asia journal of public health* 2019;8(2):71-76.
45. Organization WH. Bangladesh health system review: Manila: WHO Regional Office for the Western Pacific 2015.
46. National Institute of Population Research and Training (NIPORT), Mitra and associates, Macro International. Bangladesh Demographic and Health Survey 2007. Dhaka, Bangladesh, and Rockville, Maryland, USA, 2007.
47. Anwar S, Nasrullah M, Hosen MJ. COVID-19 and Bangladesh: Challenges and how to address them. *Frontiers in public health* 2020;8
48. Shammi M, Bodrud-Doza M, Islam ARMT, et al. COVID-19 pandemic, socioeconomic crisis and human stress in resource-limited settings: A case from Bangladesh. *Heliyon* 2020;6(5):e04063.
49. Day LT, Rahman QS-u, Rahman AE, et al. Assessment of the validity of the measurement of newborn and maternal health-care coverage in hospitals (EN-BIRTH): an observational study. *The Lancet Global Health* 2021;9(3):e267-e79.
50. Tahsina T, Hossain AT, Ruysen H, et al. Immediate newborn care and breastfeeding: EN-BIRTH multi-country validation study. *BMC pregnancy and childbirth* 2021;21(1):1-17.
51. Day LT, Gore-Langton GR, Rahman AE, et al. Labour and delivery ward register data availability, quality, and utility-Every Newborn-birth indicators research tracking in hospitals (EN-BIRTH) study baseline analysis in three countries. *BMC health services research* 2020;20(1):1-14.
52. Bhattacharya AA, Umar N, Audu A, et al. Quality of routine facility data for monitoring priority maternal and newborn indicators in DHIS2: A case study from Gombe State, Nigeria. *PLoS One* 2019;14(1):e0211265.

For peer review only

For peer review only - http://bmjopen.bmj.com/site/about/guidelines.xhtml

AUTHORS' CONTRIBUTION

SM and AER are the co-principal investigators of this study, and they conceptualized the study. SM took the lead in developing the first draft of the manuscript. SJ, SA, GB, TT, AA, ESG, SB, and MAIC provided their critical feedbacks on each draft of the manuscript. SM, ATH, NN, QSR, and AER have conducted the data analysis and interpreted the findings. All the authors have contributed significantly to designing the study conducted and preparing the final draft of the manuscript. All authors have read and approved the submission. AER, SEA, and RM have led the process as the senior authors.

Data availability statement

The data will be available upon request as per the organizations' policy.

FUNDING STATEMENT

The study was supported by the World Health Organization (WHO) under the grant of BGT-0031/2020.

COMPETING INTERESTS STATEMENT

There is no competing or conflicting interest.

ACKNOWLEDGEMENT

The authors would like to acknowledge the kind support of the Baliakandi demographic surveillance site which is supported by the CHAMPS project by Gates Foundation by Emory University throughout the data collection. icddr,b is also grateful to the Governments of Bangladesh, Canada, Sweden, and the UK for providing unrestricted/institutional support.

For peer review only - http://bmjopen.bmj.com/site/about/guidelines.xhtml

For peer review only

For peer review only

For peer review only - http://bmjopen.bmj.com/site/about/guidelines.xhtml

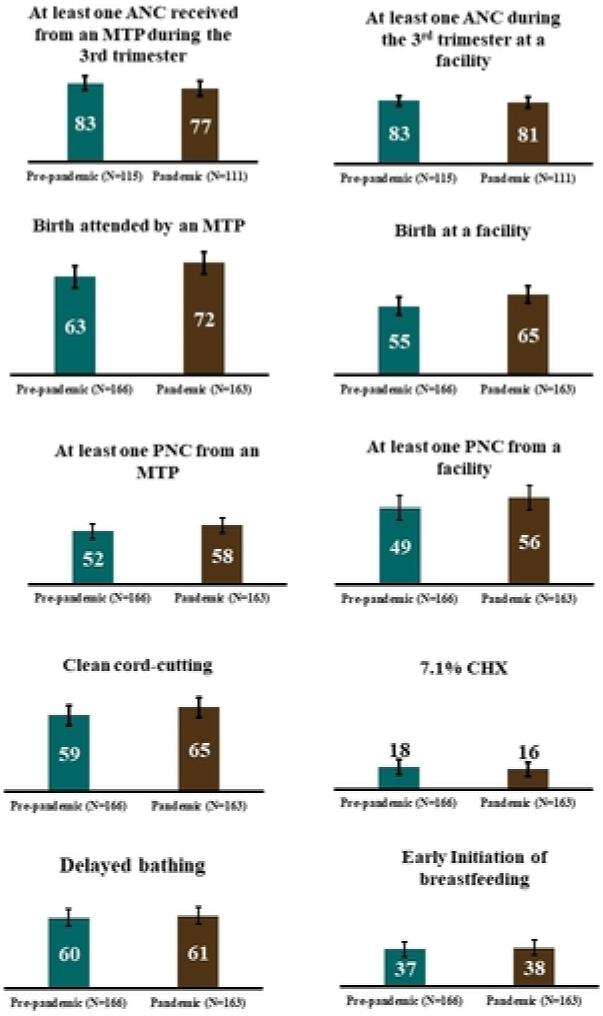

Coverage of essential MNH services during the pre-pandemic and the pandemic periods, presented in percentage with 95% confidence intervals

30x44mm (300 x 300 DPI)

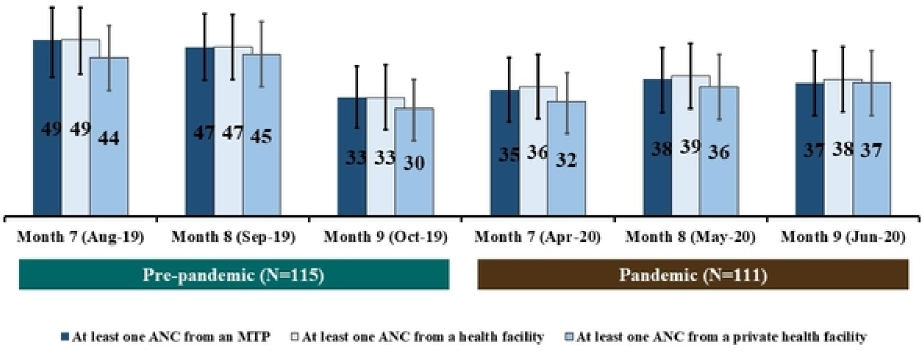

Coverage of ANC received in the third trimester during the pre-pandemic and the pandemic periods; presented in percentages by months of pregnancy

54x30mm (300 x 300 DPI)

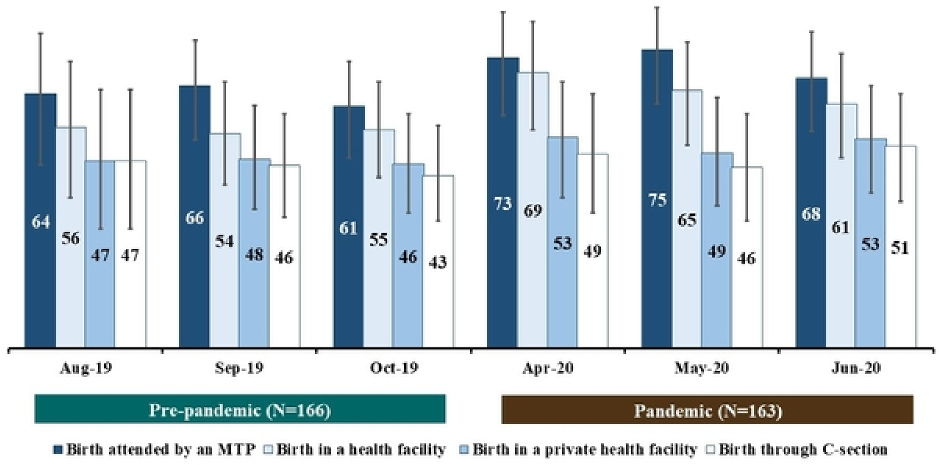

Coverage of births attended by a medically trained provider, birth in a health facility, birth in a private health facility, and birth through C-sections during the pre-pandemic and pandemic periods; presented in percentages by calendar months

54x30mm (300 x 300 DPI)

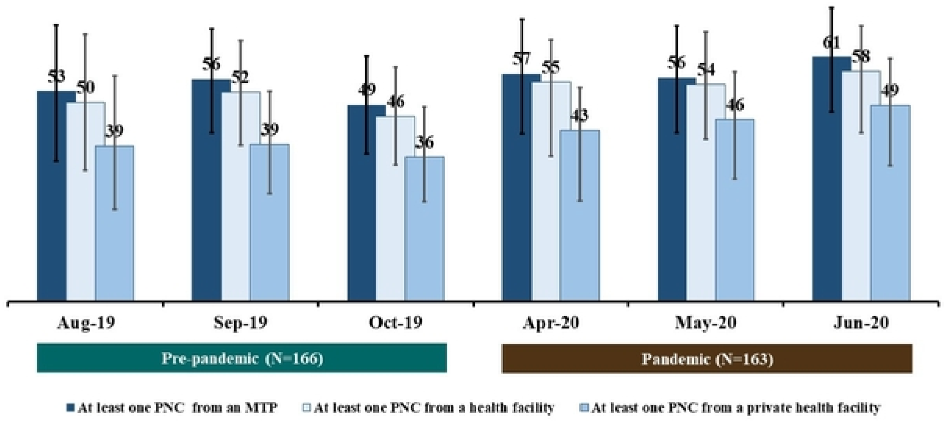

Coverage of PNC by a medically trained provider, PNC in a health facility, PNC in a private health facility among births occurred during the pre-pandemic and pandemic periods; presented in percentages by calendar months

54x30mm (300 x 300 DPI)

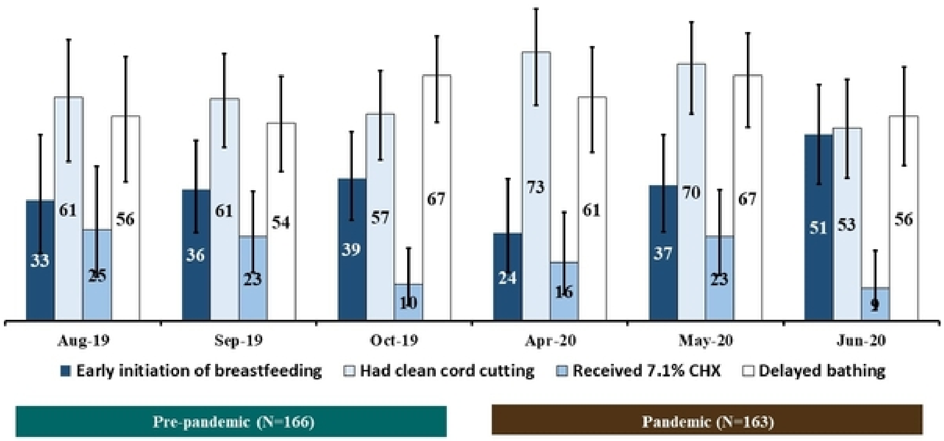

Coverage of different components of ENC during the pre-pandemic and pandemic periods; presented in percentages by calendar months

54x30mm (300 x 300 DPI)

Supplementary Table 1: Summary of indicator selected for each target population with required sample size

Target population	Indicator	Baseline (pre-pandemic period)	Endline (Pandemic period)	Unadjusted sample size	Adjustedd Sample Size
Women who were third trimester pregnant during the COVID-19' pandemic' period and the selected pre-pandemic period in the preceding year	At least one ANC from MTP<br>At least one ANC from facility	60%	30%	113	126
Women who had a history of birth during the COVID-19' pandemic' period and the selected pre-pandemic period in the preceding year	Birth attended by an MTP<br>Birth in a health facility<br>At least one PNC from an MTP within 48 hours of birth<br>Newborns received essential newborn care (ENC)	50%	33%	160	178

For peer review only

Supplementary Table 2: Definition of selected indicator

Selected indicators	Definitions
Indicator 1	Proportion of women who were third trimester pregnant during the COVID-19' pandemic' period and the pre-pandemic period in the preceding year receiving at least one ANC from a medically trained provider (MTP)
Indicator 2	Proportion of women who were third trimester pregnant during the COVID-19' pandemic' period and the pre-pandemic period in the preceding year receiving at least one ANC from facility
Indicator 3	Proportion of women giving birth during COVID-19' pandemic' period and the corresponding period in the preceding year were attended by an MTP
Indicator 4	Proportion of women giving birth during COVID-19' pandemic' period and the corresponding period in the preceding year at a facility
Indicator 5	Proportion of women giving birth during the COVID-19' pandemic' period and the pre-pandemic period in the preceding year receiving PNC from an MTP within 48 hours of birth
Indicator 6	Proportion of newborns having born during the COVID-19' pandemic' period and the pre-pandemic period in the preceding year received drying
Indicator 7	Proportion of newborns having born during the COVID-19' pandemic' period and the pre-pandemic period in the preceding year had clean cord cutting
Indicator 8	Proportion of newborns having born during the COVID-19' pandemic' period and the pre-pandemic period in the preceding year received 7.1% chlorohexidine
Indicator 9	Proportion of newborns having born during the COVID-19' pandemic' period and the pre-pandemic period in the preceding year had delayed bathing
Indicator 10	Proportion of newborns having born during the COVID-19' pandemic' period and the pre-pandemic period in the preceding year had early initiation of breastfeeding

For peer review only

Supplementary Table 3: Definition of providers and facilities

Type of providers		Definition
Medically trained provider (MTP)	Doctors (General physicians)	This provider has five years of training on medicine, surgery, gynaecology, and obstetrics with one-year internship in medicine, surgery, gynaecology, and obstetrics wards. This provider is qualified to provide ANC, Basic emergency obstetric services (BEOC), PNC, menstrual regulation (MR), abortion care (AC), family planning services (FPS).
	Nurse	This provider completes 3-4 years of nursing course with 6 months dedicated to obstetrician training. This provider is qualified to provide ANC, BEOC, PNC, MR, AC, FPS.
	Midwife	This provider completes one year of midwifery training with 6 months dedicated to obstetrician training. This provider is qualified to provide ANC, BEOC, PNC, MR, AC, FPS.
	Family welfare visitor (FWV)	This provider completes 18 months of training on maternal and child health, family planning, and contraception. This provider is qualified to provide facility-based ANC, BEOC, PNC, MR, AC, FPS.
	Community Skilled Birth Attendant (CSBA)	This provider completes 6 months of training on BEOC and ENC. This provider is qualified to provide community-based ANC, BEOC, PNC, FPS.
Non-medically trained provider (non-MTP)	Family Welfare Assistant (FWA)	This provider completes 30 days of training on family planning. This provider is qualified to provide community-based FPS.
	Health assistant (HA)	This provider completes 3 months of training on limited preventive and curative care, immunization. This provider is qualified to provide community-based immunization of mother and children.
	Sub Assistant Community Medical Officer (SACMO)	This provider does not have any obstetrician training.
	Community Health Care Provider (CHCP)	This provider does not have any obstetrician training.
	Medical assistants (MA)	This provider completes 3 years of training on treatment of common disorders.

For peer review only

Supplementary Figure 1: Sampling frames and sample size during pre-pandemic and pandemic period

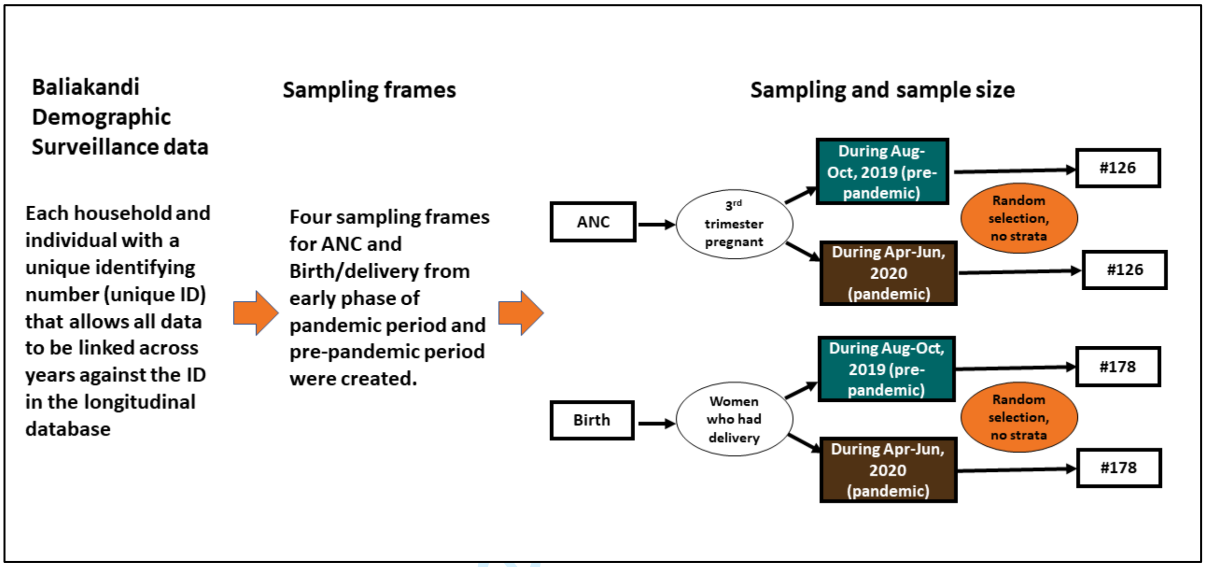

For peer review only

Supplementary Figure 2: Proportion of third trimester pregnant women who received ANC from a medically trained provider during pre-pandemic and pandemic period (N=226)

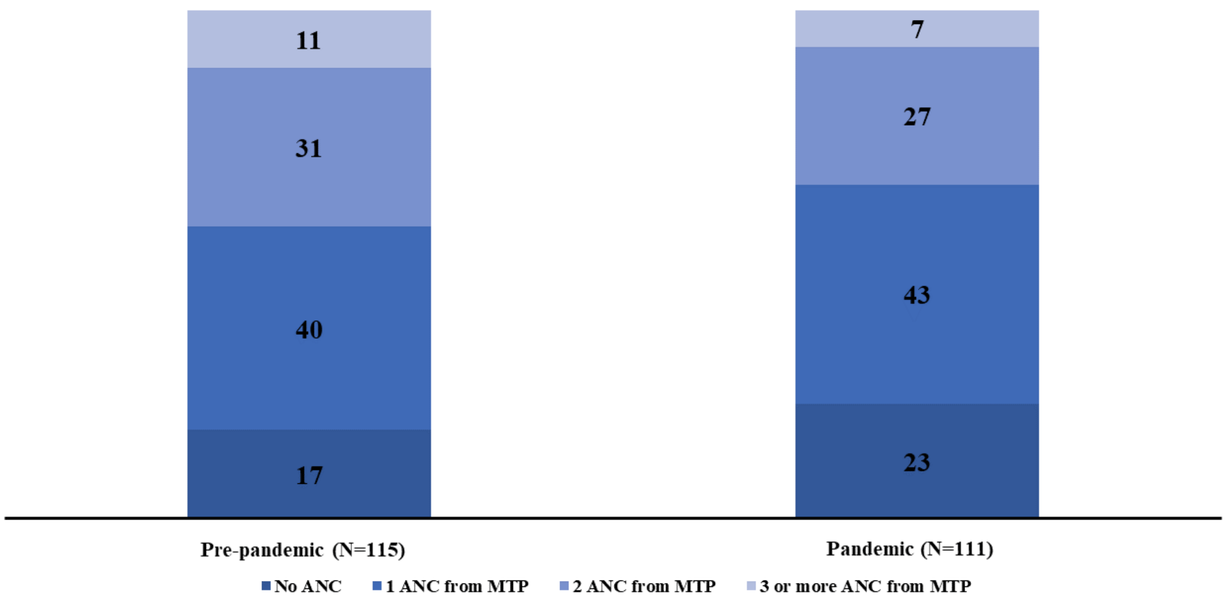

For peer review only - http://bmjopen.bmj.com/site/about/guidelines.xhtml

Supplementary Figure 3: Awareness and use of telemedicine services among the respondents (N=555)

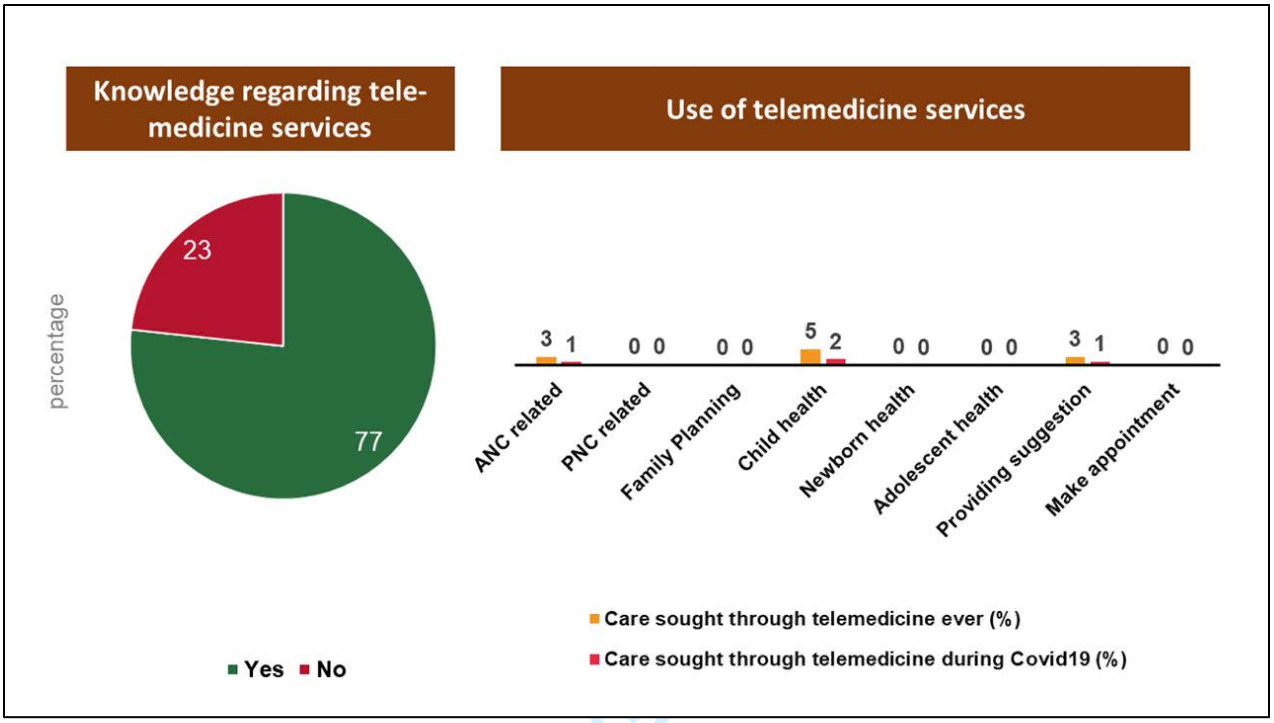

STROBE Statement—Checklist of items that should be included in reports of ***cross-sectional studies***

	Item No	Recommendation
**Title and abstract**	1	(*a*) Indicate the study's design with a commonly used term in the title or the abstract
		(*b*) Provide in the abstract an informative and balanced summary of what was done and what was found
**Introduction**		
Background/rationale	2	Explain the scientific background and rationale for the investigation being reported
Objectives	3	State specific objectives, including any prespecified hypotheses
**Methods**		
Study design	4	Present key elements of study design early in the paper
Setting	5	Describe the setting, locations, and relevant dates, including periods of recruitment, exposure, follow-up, and data collection
Participants	6	(*a*) Give the eligibility criteria, and the sources and methods of selection of participants
Variables	7	Clearly define all outcomes, exposures, predictors, potential confounders, and effect modifiers. Give diagnostic criteria, if applicable
Data sources/ measurement	8*	For each variable of interest, give sources of data and details of methods of assessment (measurement). Describe comparability of assessment methods if there is more than one group
Bias	9	Describe any efforts to address potential sources of bias
Study size	10	Explain how the study size was arrived at
Quantitative variables	11	Explain how quantitative variables were handled in the analyses. If applicable, describe which groupings were chosen and why
Statistical methods	12	(*a*) Describe all statistical methods, including those used to control for confounding
		(*b*) Describe any methods used to examine subgroups and interactions
		(*c*) Explain how missing data were addressed
		(*d*) If applicable, describe analytical methods taking account of sampling strategy
		(*e*) Describe any sensitivity analyses
**Results**		
Participants	13*	(a) Report numbers of individuals at each stage of study—eg numbers potentially eligible, examined for eligibility, confirmed eligible, included in the study, completing follow-up, and analysed
		(b) Give reasons for non-participation at each stage
		(c) Consider use of a flow diagram
Descriptive data	14*	(a) Give characteristics of study participants (eg demographic, clinical, social) and information on exposures and potential confounders
		(b) Indicate number of participants with missing data for each variable of interest
Outcome data	15*	Report numbers of outcome events or summary measures
Main results	16	(*a*) Give unadjusted estimates and, if applicable, confounder-adjusted estimates and their precision (eg, 95% confidence interval). Make clear which confounders were adjusted for and why they were included
		(*b*) Report category boundaries when continuous variables were categorized
		(*c*) If relevant, consider translating estimates of relative risk into absolute risk for a meaningful time period
Other analyses	17	Report other analyses done—eg analyses of subgroups and interactions, and sensitivity analyses

For peer review only

For peer review only - http://bmjopen.bmj.com/site/about/guidelines.xhtml

**Discussion**		
Key results	18	Summarise key results with reference to study objectives
Limitations	19	Discuss limitations of the study, taking into account sources of potential bias or imprecision. Discuss both direction and magnitude of any potential bias
Interpretation	20	Give a cautious overall interpretation of results considering objectives, limitations, multiplicity of analyses, results from similar studies, and other relevant evidence
Generalisability	21	Discuss the generalisability (external validity) of the study results
**Other information**		
Funding	22	Give the source of funding and the role of the funders for the present study and, if applicable, for the original study on which the present article is based

*Give information separately for exposed and unexposed groups.

Note: An Explanation and Elaboration article discusses each checklist item and gives methodological background and published examples of transparent reporting. The STROBE checklist is best used in conjunction with this article (freely available on the Web sites of PLoS Medicine at http://www.plosmedicine.org/, Annals of Internal Medicine at http://www.annals.org/, and Epidemiology at http://www.epidem.com/). Information on the STROBE Initiative is available at www.strobe-statement.org.

For peer review only

For peer review only - http://bmjopen.bmj.com/site/about/guidelines.xhtml
